# Positively Charged Residues Are the Major Determinants of Ribosomal Velocity

**DOI:** 10.1371/journal.pbio.1001508

**Published:** 2013-03-12

**Authors:** Catherine A. Charneski, Laurence D. Hurst

**Affiliations:** Department of Biology and Biochemistry, University of Bath, Bath, United Kingdom; Fred Hutchinson Cancer Research Center, United States of America

## Abstract

Both for understanding mechanisms of disease and for the design of transgenes, it is important to understand the determinants of ribosome velocity, as changes in the rate of translation are important for protein folding, error attenuation, and localization. While there is great variation in ribosomal occupancy along even a single transcript, what determines a ribosome's occupancy is unclear. We examine this issue using data from a ribosomal footprinting assay in yeast. While codon usage is classically considered a major determinant, we find no evidence for this. By contrast, we find that positively charged amino acids greatly retard ribosomes downstream from where they are encoded, consistent with the suggestion that positively charged residues interact with the negatively charged ribosomal exit tunnel. Such slowing is independent of and greater than the average effect owing to mRNA folding. The effect of charged amino acids is additive, with ribosomal occupancy well-predicted by a linear fit to the density of positively charged residues. We thus expect that a translated poly-A tail, encoding for positively charged lysines regardless of the reading frame, would act as a sandtrap for the ribosome, consistent with experimental data.

## Abstract 2

Positively charged amino acid residues in the nascent peptide, not RNA-level features as long thought, slow ribosomes.

## Author Summary

Ribosomes do not synthesize protein at a constant rate along transcripts, and changes in translation speed can have knock-on consequences for the expression of that protein, even altering its folding or subcellular localization. It has long been thought that RNA-level features modulate translation rates, whether by delays incurred through the presence of codons that require relatively rare tRNAs, or by regions of mRNA folding that physically impede ribosomal progression. We find on the contrary that it is not RNA-level features but positive charges in the already translated protein that most retard ribosomes, possibly by interacting with the negatively charged ribosomal exit tunnel. We show that positive charge explains the sites where ribosomes stall most commonly within transcripts. We also show why, if protein charge were not considered, one could be misled into suspecting a role for non-optimal codons. Finally, we observe that the poly-A tail provides a massively positively charged terminus no matter in which frame it is translated. A missed stop codon or frameshifting would then lead to a stalled ribosome, which is consistent with experimental data.

## Introduction

While it is known that there is great variation in ribosomal velocity along even a single transcript [1], what determines how fast a transcript (or part thereof) is processed is unresolved. Resolving this issue is important for understanding causes of disease and for the generation of transgenes, as changes in the local translation rate along mRNAs have been implicated in the regulation of protein folding [2], error attenuation processes such as no-go decay in yeast [3], transcription attenuation in bacterial systems [4], and correct protein localization [5],[6].

For some time it has been hypothesized [7]–[10], and commonly assumed (e.g., [11],[12]), that codons matching rare tRNAs slow ribosomes along transcripts due to differential tRNA availability. The supposition is that codons corresponding to less abundant tRNAs are translated at slower rates as the ribosome must pause while the appropriate tRNA becomes available. This, for example, is held up to explain the usage of codons specified by the most abundant tRNAs in the most highly expressed genes [13],[14]. Although the notion that rare codons must stall ribosomes is commonplace, recent work has started to undermine the supposition that differential usage of synonymous codons will significantly alter the rate of ribosomal translocation within a transcript under normal conditions [15]–[17]. Indeed, much of the evidence cited as support for an effect on translational speed is questionable (see Note S1) and many of the patterns attributed to selection for translational speed are better explained in terms of selection on codon usage for translational accuracy [18]–[21].

Codon usage, however, is not the only potential factor affecting elongation speed. Double-stranded mRNA hairpin or pseudoknot structures are thought to impede progress of the ribosome [22],[23]. The generality of this during elongation, however, is unclear, as other studies [24] suggest that the ribosome can more readily melt moderately stable secondary structures once initiation has taken place.

While the above factors consider ribosomal velocity to be modulated by properties of the mRNA, much less attention has been paid to the possibility that the resultant protein might impact translation rates. However, recent experimental work on recombinant peptides has shown that positive charges on the newly synthesized peptide might slow ribosomes [25],[26]. This is conjectured to be owing to an electrostatic interaction between the cation in the emerging polypeptide and the negatively charged exit tunnel of the ribosome [25],[26]. Following on from this, it has been suggested that positive charges, codon usage bias, and transcript folding play a role in ribosomal stalling at 5′ transcript ends [27],[28].

Here we ask not whether certain features can sometimes modulate translation speed along a transcript (e.g., when grossly overrepresented in transgenes; see Note S1), but if they do as evolved in endogenous genes when expressed at “normal” levels, and to what extent. Ribosomally protected mRNA footprints from an experimental *Saccharomyces cerevisiae* dataset [29] enable us to profile the location of ribosomes across the *S. cerevisiae* transcriptome. Under the assumption that ribosomal densities inversely reflect ribosomal velocity [30],[31], we independently examine the effects of codon usage, mRNA folding, and positive charge on ribosomal speed throughout endogenous yeast genes. We show that positive charges in the nascent peptide slow the ribosome along transcripts in an additive manner in vivo, and that this slowing effect cannot be accounted for by mRNA structure, and even far surpasses that (if any) induced by codon usage bias. Within transcripts, those regions with the highest ribosomal occupancy are those most likely to be just downstream of positively charged residues. The cation sandtrap effect has potential relevance for the evolution of the poly-A tail, specifying as it does a series of positively charged amino acids if translated.

## Results

While some recent work on nucleotide-resolution ribosomal footprint data [29] has claimed that codon usage plays a role in slowing ribosomes [27],[28], another study that examined the same footprint data, filtered for noise, contradicts this claim [16]. Here we reanalyze the same dataset using both stringent mapping to reduce false-positive footprints (see Methods, “Ribosomal Density Data” for further comments on this and previous studies) as well as a novel normalization method to detect any accrual of ribosomal density, on average across transcripts, after putative ribosome-slowing features.

### Neither Clusters of Nor Consecutive Rare Codons Tend to Slow Ribosomes

**Figure 1:**
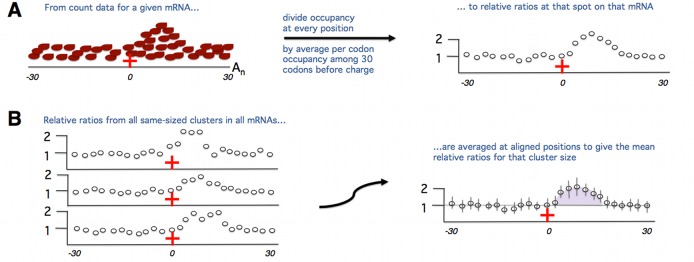
Visual overview of our plotting analyses. A feature of one codon encoding a positive charge as a potential slower of translation elongation is considered as an example. The feature of interest (here the encoded charge) must be surrounded by no other codons encoding positive charges for 30 codons in both directions so as to not interfere with our measurement of slowing due to the single encoded charge we have identified. (A) We start with footprint data, which we have stringently mapped to the codons surrounding the encoded positive charge of interest on the mRNA in which the encoded charge resides. We first count the ribosomal footprints mapping to each codon position in this area. We take the average of the ribosomal footprint counts among the 30 codons preceding (the start of) the feature. We consider the average footprint counts of these preceding 30 codons (*r_prec30_*) to reflect the baseline speed at which ribosomes are translating before they reach the encoded charge. We then divide the ribosomal footprint counts in each of the 61 codon positions in this section of the mRNA by *r_prec30_* to measure whether they are more densely or sparsely covered with ribosomal footprints in a given codon position relative to the density before the feature. Note the ratios prior to *x* = 0 will tend to center around 1 as they will have been normalized by a value likely close to their own. We calculate these relative ratios separately for every feature cluster in every mRNA we identify as suitable for our analysis. (B) To ask whether there is a trend in slowing upon the translation of the feature of interest (the single positive charge in this example), we align all of the mRNAs with the feature of interest by (the start of) the feature. We determine the average relative change in ribosomal density upon translation of the feature by averaging each of the ratios calculated in (A) for each aligned codon surrounding the feature. It is these mean ratios we consider when we calculate the slowing effect (if any) of a given feature. The degree of slowing due to a feature is a function of both the magnitude of the footprint buildup on any one codon as well as the length along the mRNA that the buildup extends. We hence calculate the slowing due to the feature (here the single positive charge) by summing the area between the line *y* = 1, which represents the baseline speed (see A) and the mean relative ratios between the start of the feature at *x* = 0 and the point where the means cross *y* = 1 again (highlighted purple area). If the line does not intersect with *y* = 1 again by the end of the window (*x* = 30), the entire area under the curve from *x* = 0 to *x* = 30 was used. We do not consider codons at *x*>30 as there may be positive charges encoded in this downstream region that we do not wish to interfere with our measurements. In some cases, not slowing but speeding will occur, indicated by ratios that are less than 1 (not shown). In this case, we calculate the degree of speeding similarly, by summing the area between the mean ratios and *y* = 1.

Ribosomal footprint data [29] allow us to examine changes in the rate of translation given the assumption that the slower a ribosome travels along a given portion of a transcript, the more likely it is to be found there at any point in time [30],[31]. In the case of codon usage, we expect to see any possible ribosomal stalling centered over the rare codon(s) while the ribosome awaits a tRNA to enter its A-site. Hence to examine the effect of a sequence feature such as rare codons on the speed of translation, we calculate the relative change in stringently mapped ribosomal densities that occurs within a single transcript as ribosomes begin to translate regions of transcript enriched for rare codons (see Methods and Figure 1). To this end, within each transcript we compared the ribosomal occupancy at codon positions (*r_pos_*) in the vicinity of clusters of rare codons (*r_pos_*) to the average ribosomal occupancy of the 30 codons immediately preceding the first rare codon in the cluster (*r_prec30_*). We then averaged the relative increase or decrease in ribosomal occupancy across transcript sections aligned by rare codon clusters. A mean *r_pos_*/*r_prec30_* after the clusters >1 indicates a denser sampling of ribosomal footprints on average and hence slowing at that codon position, while a mean *r_pos_*/*r_prec30_*<1 denotes sparser ribosomal coverage, consistent with acceleration.

In our main analysis we make use of the tAI (range 0–1) as a measure of codon optimality as this metric uniquely reflects the tRNA pool. The tAI of a sequence is defined as the geometric mean of the relative adaptiveness of its constituent codons to the tRNA pool available in that organism [32]. A higher tAI indicates the codon has a high abundance of decoding isoacceptor tRNAs and, according to the codon usage hypothesis of translational speed, should be translated faster on account of its ready coupling with an aminoacylated tRNA. A lower tAI conversely indicates a codon that is matched by a low number of tRNAs and is therefore putatively slowly translated and nonoptimal. Here we define “rare” codons to be those in the lowest quartile of tAI values (Methods, “The Average Effect of Codon Usage on Ribosomal Densities”) (see also Figures S1, S2, S3 and Table S1 for analysis of rare codons defined according to genomic frequency).

**Figure 2:**
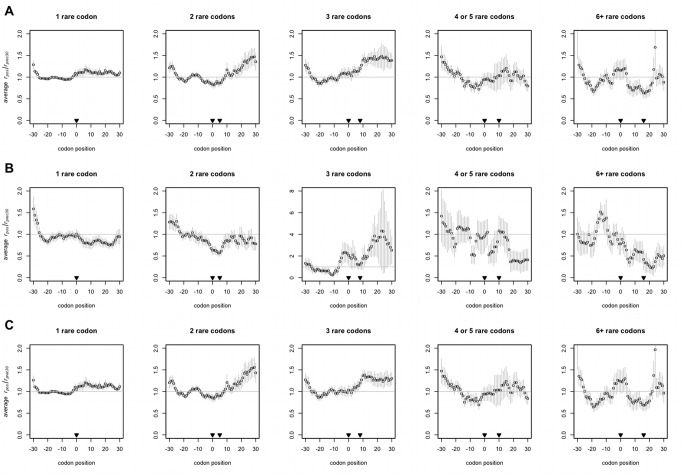
Clusters of rare codons do not tend to slow ribosomes. The first of the number of nonoptimal codons indicated always occurs at *x* = 0, and the rest, if any, may be found at points up to and including the codon indicated by the second arrowhead. The mean *r_pos_/r_prec30_*, or relative change in ribosomal occupancy, at each position across aligned transcripts ± s.e.m. is plotted. The horizontal at *y* = 1 represents the null expectation that positive charges do not alter ribosomal speed—that is, that ribosomes are, on average, as frequently present before the rare codon cluster as after it. The three-rare codon plot in (B) is plotted with different axes as it is an outlier. Some residual slowing is observed near *x* = −30 on all plots due to slowing elements (e.g., positive charges) that may be encoded just upstream (*x*<−30). (A) All genes with rare codon clusters. (B) Genes with rare codon clusters that have 0 or 1 positive charges coded for in the last 30 codon positions plotted. These plots represent the net effect of tAI on ribosomal density, with the bulk of the effect of positive charge removed. (C) Genes with rare codon clusters that have two or more positive charges in the last 30 codon positions plotted.

**Figure 3:**
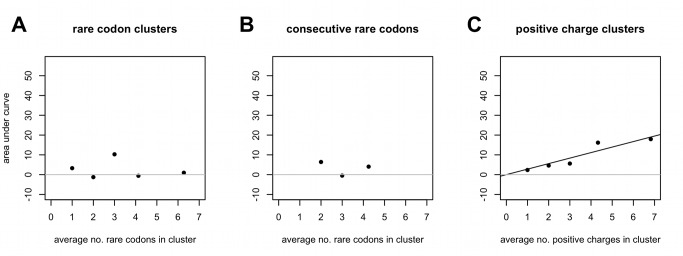
Positive charges show an additive (linear) trend in slowing ribosomes, but rare codons do not. The degree of slowing is a function of both the magnitude of ribosomal density and the length of transcript the slowing covers. Therefore, to measure any trend in the ability of either positive charges or codon clusters to slowing, the area between the curves depicting the average relative change in ribosomal density (*r_pos_/r_prec30_*) and the *y* = 1 null in Figure 2A, Figure S5A, and Figure 5, whether positive or negative, was summed between *x* = 0 (the beginning of the cluster) and the point where the plotted values intersect with *y* = 1 again, regardless of where the last charge in the cluster is (see Figure 1 for further explanation of the area under the curve). A positive value for the area under the curve indicates ribosomal slowing after the feature in question, while a negative value reflects faster movement. (A and B) Regression of *area under curve*∼*size of cluster*, slope *p* = 0.45 and 0.33, respectively. (C) Regression of *area under curve*∼*size of cluster* gives a slope of 2.81 (*p* = 0.020), *r* ^2^ = 0.93. To achieve such a regression slope in the set of genes used is significantly nonrandom (*p* = 0.011, Note S3).

Our results show inconsistent trends in ribosomal occupancy after rare codon clusters when all clusters of a given size are aligned and the average increase in ribosomal density after the cluster (here uncontrolled for covariates) is plotted (Figure 2A). This inconsistency is still apparent when we consider rare codons to be not those with a low tAI but those that are genomically infrequent (Figure S1). If there is any slowing due to rare codons, we should expect an increase in the amount of slowing along the mRNA as the number of rare codons increases. However, no such trend is evident (Figure 3A). This lack of influence of rare codon usage on ribosomal speed is not owing to a covariance between rare codon clusters and expression levels (Table S2). Shifting the location of the “preceding 30 codons” we use to normalize footprint values slightly upstream, to accommodate the 5′ portion of the ribosome potentially slowed over a rare codon, still detects no slowing due to codon usage (Figure S4).

As it has been postulated that tandem nonoptimal codons may more strongly inhibit progression of the ribosome than scattered rare codons [33],[34], we also investigated whether consecutive rare codons (adjacent codons, each from the lowest quartile of tAI values) may be affecting ribosomal velocity. Examining changes in ribosomal densities after pairs, triplets, and so forth of rare codons, however, also indicates that consecutive rare codons do not systematically slow ribosomes (Figure S5 and Figure 3B). We achieve similar findings when defining rare codons according to their genomic frequency (Figure S2).

If the above results are correct, then we should also find that codon usage cannot explain ribosomal slowing when we compare sites within a given mRNA. Upon locating the highest and lowest ribosomal occupancy portions within a given mRNA, we determined whether the denser region was associated with a putative ribosome-slowing feature: lower tAI, or more rare codon pairs or rare 6-mers (two adjacent in-frame codons that, as a pair, come from the lowest 10% of all 6-mers within the genome) (see Methods, “The Relative Contributions of Charge, Folding, and Codon Usage to Extremes of Slowing Within Transcripts”). Considering all transcripts, the most slowly translated region within an mRNA in fact tends to be comprised of more optimal codons or fewer rare pairs, suggesting low codon optimality does not cause slowing (Table 1A,B and Figure S6). These results are not affected if we consider suboptimal codons to be those that are genomically infrequent (Table S1 and Figure S3). Nor do we find that transcript similarity to the yeast Kozak sequence can explain slowing within these regions (Figure S7 and Table S3). Additionally, as the difference in ribosomal occupancy between the two intra-transcript windows increases (and hence the presumed difference in the inferred ribosomal velocities between the two windows grows all the more), the already low proportion of transcripts for which tAI, genomic infrequency, or presence of rare pairs could possibly explain ribosomal pausing in fact decreases (Table 1A,B and Table S1). In other words, in transcripts that have the greatest differences in ribosomal densities along their length (as inferred from the highest and lowest ribosomal occupancy windows), and hence that contain the greatest degree of internal slowing relative to maximum translation speed, the most ribosomally occluded windows are even more likely to be comprised of more optimal codons. This indicates that not only is low codon optimality incapable of explaining ribosomal slowing in general, it is even less capable of explaining the greatest relative slowing within a transcript.

**Table 1:** Only positive charge is systematically capable of explaining ribosomal slowing, including the severest slowing.

Score	Value	q1_Δ*r*_ (Count)	q2_Δ*r*_	q3_Δ*r*_	q4_Δ*r*_	?^2^ Test for Heterogeneity/*p* Value (Bonferroni Correction)
A. tAI score	1	590	597	563	525	0.13
	0	0	0	0	0	—
	−1	656	649	682	721	0.20
	Binomial test on +1 and −1 tAI score counts, *p* value (Bonferroni correction)	0.065 (0.26)	0.15	0.00082 (0.003)	3.1e-08 (1.2e-07)	—
B. rare pair score	1	175	179	144	86	3.0e-08 (8.9e-08)
*rare 6-mer score*		*127*	*106*	*86*	*45*	*9.5e-09 (2.9e-08)*
	0	858	885	905	1,037	0.00013 (3.8e-04)
		*383*	*403*	*424*	*503*	*0.00023 (0.00069)*
	−1	213	182	196	123	1.10e-05 (3.3e-05)
		*199*	*199*	*198*	*161*	*0.13*
	Binomial test on +1 and −1 rare pair score counts, *p* value (Bonferroni correction)	0.060	0.92	0.0056 (0.022)	0.013 (0.050)	—
		*7.9e-05 (3.2e-04)*	*1.1e-07 (4.4e-07)*	*2.5e-11 (1.0e-10)*	*<2.2e-16 (8.8e-16)*	—
C. PARS score	1	86	72	81	55	0.060
*Conservative PARS score*		*302*	*272*	*290*	*294*	*0.64*
	0	469	512	500	546	0.11
		*0*	*0*	*0*	*0*	*—*
	−1	154	124	127	108	0.036 (0.11)
		*407*	*436*	*418*	*415*	*0.78*
	Binomial test on +1 and −1 PARS score counts, *p* value (Bonferroni correction)	1.3e-05 (5.2e-05)	0.00025 (0.001)	0.0017 (0.0068)	4.0e-05 (0.00016)	—
		*9.1e-05 (0.00036)*	*7.6e-10 (3.0e-09)*	*1.7e-06 (6.8e-06)*	*6.3e-06 (2.5e-05)*	*—*
D. charge score	1	573	586	637	717	0.00014 (0.00043)
	0	258	259	236	207	0.0589 (0.18)
	−1	415	401	372	322	0.0038 (0.011)
	Binomial test on +1 and −1 charge score counts, *p* value (Bonferroni correction)	5.6e-07 (2.2e-06)	4.3e-09 (1.7e-08)	<2.2e-16 (8.8e-16)	<2.2e-16 (8.8e-16)	—

Quantiles of the difference in average ribosomal density between the most highly occupied and most lowly occupied windows identified within a transcript are shown, with q1 representing the smallest differences and q4 the largest. A score of 1 indicates the putative retarding feature is more present within the more occluded intra-transcript window; −1, less present; 0, present in both windows in equal amounts. Related yet alternative ways of calculating both the rare pair and PARS scores are given in italics (see Methods, “The Relative Contributions of Charge, Folding, and Codon Usage to Extremes of Slowing Within Transcripts” for details). A low codon optimality, if anything, tends to pair more with the less dense (faster translated) window. Similarly, not only do rare pairs and rare 6 -mers tend to be found more often in the faster translated window, but their presence decreases as the difference in degree of ribosomal slowing grows. Additionally, a greater likelihood of transcript secondary structure at or just before the identified window is associated not with the more occluded windows, but with the less dense (faster translated) ones, and the presence of secondary structure in fact decreases as the difference in ribosomal slowing between the windows increases. Positive charge, however, is consistently associated with the higher density (more slowly translated) window, and increasingly so as the difference in densities between the two windows becomes larger. Window pairs that have the same number of charges each (charge score, 0) do not show such a trend between quantiles.

We note that the decrease in the ability of codon usage to explain slowing in the upper quantiles (Table 1A) is simply a side effect of differential amino acid usage between the two windows. When we control for differential amino acid content between the two windows, we no longer see the decrease in the ability of codon usage to explain slowing, but codon usage still remains unable to explain the slowing that is observed in any of the quantiles (Table S4). Thus, in addition to the above finding that codon usage becomes less able to explain slowing as the degree of slowing grows (as deduced from observed transcripts), this amino-acid-controlled analysis suggests that even if amino acid sequence had evolved in any other way, codon usage would still not be a factor in the slowing of ribosomes.

It is possible that codon usage could have different effects during different times of cell cycle if tRNA levels fluctuate [35]. We do not, however, detect a systematic influence of codon usage on ribosomal speed even under amino acid starvation conditions (Figures S8, S9, S10 and Table S5) when presumably tRNA charging levels are lower, making codon usage potentially more rate-limiting [36],[37].

### RNA Structure on Average Increases Ribosomal Occupancy Marginally

If neither codon usage nor consecutive rare codons can explain variation in ribosomal speed, then what can? As it has been suggested that transcript structure can impede ribosomes along the length of the transcript [28], we next investigated whether RNA structure might be the major contributor to slowing.

We used empirically determined (rather than computationally predicted) RNA structure data (PARS values, see Methods, “The Average Effect of Transcript Structure on Ribosomal Densities”) [38]. *S. cerevisiae* protein-coding sequences were scanned for stretches whose average PARS value was 0 or negative (and hence tending to be single-stranded), which were immediately followed by a block of codons whose average PARS value was positive (i.e., with propensity for double-strandedness). The general contribution of folding to slowing was examined by calculating the relative change in ribosomal density (*r_pos_*/*r_prec30_*) at each position of the identified region of a transcript, where *r_prec30_* is the average ribosomal occupancy in the single-stranded block. We then take the average of this ratio across transcripts aligned by identified blocks of structure.

The method is similar to that used above with codons, but with one complication. In the case of codon usage, we have a prior expectation that any ribosomal pausing should occur while the ribosome is positioned over the “slow” codon. It is not immediately clear, however, where along the transcript we should expect any structure-induced pausing to take place. After translating an unstructured span of mRNA, will the ribosomal active site be able to get very close to the first double-stranded ribonucleotide it meets before it is finally slowed, or might pausing take place more 5′ if the ribosome progression is sterically occluded at some distance upstream? We investigated both hypotheses.

**Figure 4:**
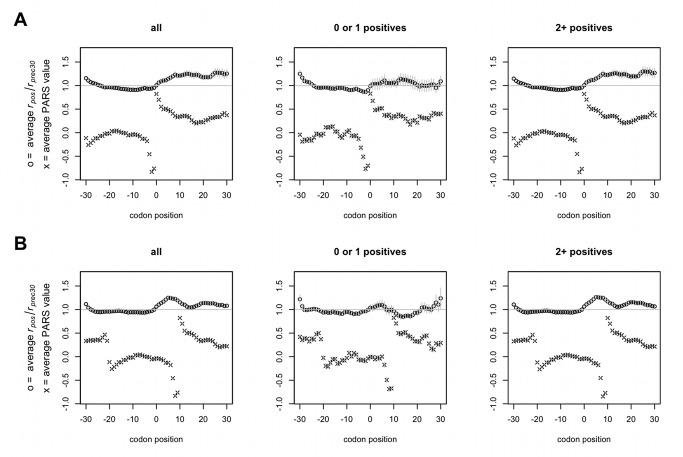
Ribosomes travelling along single-stranded RNA are not greatly retarded upon traversal into double-stranded structures. PARS values >0 denote structured mRNA, <0 single-stranded. All averages plotted (± s.e.m.) are calculated across transcripts aligned by blocks of mRNA structure. The slowing of ribosomes (*r_pos_*/*r_prec30_*>1) relative to the preceding 30 codons starting from both the beginning of double-stranded structure (A) and 10 codons upstream of the same regions of double-stranded structure (B) are shown. In both cases, there is a degree of translational pausing observed upon the transition into folded mRNA, although some of this slowing may be caused by the presence of two or more positive charges encoded in the folded area (0≥*x*≤30).

We cannot immediately distinguish between the possibilities that mRNA folding has an effect on ribosomal progression either upon or upstream of the folded ribonucleotides in question, as some degree of pausing is observed in both cases (Figure 4). But how strong is this slowing effect? Could mRNA folding account for the bulk of the variance in ribosomal speed observed along transcripts? We find, again comparing the slowest and fastest translated regions within a given mRNA, that not only is secondary structure incapable of systematically explaining the slowest regions of translation, but the presence of secondary structure decreases as the difference between the ribosomal density (i.e., difference in translation speed) of the two intra-transcript windows increases (Table 1C). Hence we conclude something other than mRNA folding must be responsible for the greatest slowing within transcripts.

### Positively Charged Amino Acids Additively Slow Ribosomes on Endogenous Yeast Transcripts

We performed a parallel version of the codon cluster analysis to look for changes in ribosomal density after differently sized clusters of encoded positive charges (see Methods, “The Average Effect of Positive Charge on Ribosomal Densities”), calculating the average relative change in ribosomal density within a transcript (*r_pos_*/*r_prec30_*) after positively charged residues (lysine, arginine, or histidine) are added to a nascent peptide chain. The effect, note, should be a stalling after the codon specifying the charged amino acid as the stalling process is hypothesized to be an interaction between the charged amino acid and the charged exit tunnel [26],[39].

**Figure 5:**
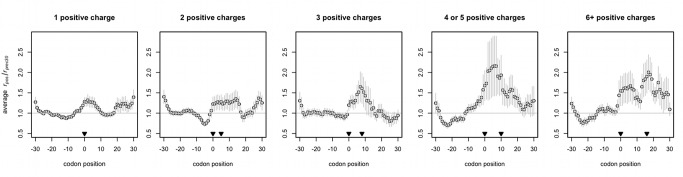
Positive charges slow ribosomes. The first of the positive charges indicated always occurs at *x* = 0, and the rest, if any, may be found at points up to and including the codon indicated by the second arrowhead. *r_pos_/r_prec30_* is the ribosomal occupancy at position *x* normalized by the average occupancy of the 30 codons preceding the encoded positively charged cluster within the same transcript. The mean *r_pos_/r_prec30_*, or average relative change in ribosomal occupancy, at each position across aligned transcripts ± s.e.m. is plotted. The horizontal at *y* = 1 represents the null expectation that positive charges do not alter ribosomal speed; in other words, that ribosomes which translate in positive-charge free peptides are, on the average, as frequently present before the charge cluster as after it.

We find that a single positive charge will slow the ribosome relative to the preceding sequence (Figure 5), regardless of whether the codon encoding the residue is A/G- or C-rich (Figure S11). Our findings show that at maximum (in real transcripts), ribosomes are more than twice as likely to be found at a given region of the transcript as before the addition of the cation to the polypeptide (Figure 5). The higher the density of positive charges in a peptide, the proportionally greater the effect (Figure 3C), in agreement with experimental findings that increasing the number of positive charges locally correspondingly increases ribosomal dwell time [26]. Our estimation of charge-induced pausing is conservative since some ribosomal density after charges is not included in the analysis if the mean ribosomal occupancy of the 30 codons preceding a charged cluster is 0 for a given transcript (our method in this case would require division by 0).

We can also test whether charge is responsible for slowing by noting that the pKa, and hence overall net charge, of histidine is lower than that of either arginine or lysine at physiological pH. Thus we should expect a weaker slowing effect due to histidine residues being added to the polypeptide. When we re-calculate the slowing effect after a single positive charge (as shown in Figure 5, first panel), but separate the single charges according to whether or not they are histidine, we indeed observe that histidine causes weaker slowing (Figure S12). The slowing effect after a single histidine residue, as calculated using the area under the curve method, is anywhere from 25%–78% (95% CI) of the slowing found after a single lysine or arginine. As histidine is used much less frequently than either of the other positively charged residues, we consider slowing after single positive charges to be the best comparator due to the larger sample sizes available. When we separate larger positive charge clusters according to their histidine content (at least one histidine in the two- or three-charge clusters, and at least two histidines in the four- or five-charge clusters), we note that the slowing due to the histidine-enriched group is always lesser than that after the histidine-free group (Figure S12).

If charge is a major determinant of ribosomal slowing, then it should be capable of explaining the regions of greatest translational pausing within transcripts (see Methods, “The Relative Contributions of Charge, Folding, and Codon Usage to Extremes of Slowing within Transcripts”). We find this is indeed the case. Of all the putative slowing features we consider, only positive charge is more often associated with the higher occupancy window within each transcript (Note S2). Breaking the comparisons into quantiles according to the magnitude of difference in ribosomal occupancy between each pair of windows further reveals that positive charge is the feature most often responsible for not just slowing when comparing between transcripts, but the greatest magnitude of slowing within any given mRNA. As the difference in ribosomal occupancy between the two windows increases, the window with the higher ribosomal occupancy tends increasingly to be the one with more positive charges (Table 1D). In fact the only clearly significantly overused amino acid in the higher occupancy windows is lysine, which is positively charged (Figure S13). This increase in ribosomal occupancy cannot be explained by physiochemical properties of other amino acids, namely hydropathy, negative charge, or polarity (Tables S6, S7, S8, S9). We note that even when both windows in a transcript have the same number of charges each, there is no predominant influence of tAI, rare codon pairs, or RNA structure on ribosomal slowing (Tables S10, S11, S12).

### The Effect of Positive Charge Is Not Explained by Covariance with Codon Usage or mRNA Folding

The positive charge effects seen above could potentially be explained as covariate to codon usage bias, were, for example, codons specified by rare tRNAs especially abundant near those specifying positively charged residues. Given the absence of evidence for codon usage bias to affect translation rates, this now seems unlikely. To nonetheless test whether this is the case, we examined patterns of codon usage in the vicinity of positive charges similarly to the manner in which we investigated changes in ribosomal occupancy after positively charged clusters above. Thus if nonoptimal codon usage were causing the slowing patterns after encoded positive charges observed in Figure 1, we should see, on average, a relative decrease in tAI in those sites with elevated ribosomal occupancy. Contrary to this expectation, however, the trend for ribosomal occupancy to increase after positive charges (Figure 5) is independent of patterns of codon usage (Figure S14).

It is also possible the slowing effects observed after positive charge clusters in Figure 5 occur ancillary to mRNA secondary structure, as such structure may have some slowing effect (Figure 4). Again we allow for mRNA folding to impede the flow of ribosomes starting either locally or 10 codons upstream (in the case that local double-strandedness creates a structure within the transcript that sterically occludes ribosomes from progressing further toward codons within the folded structure). We find that patterns of transcript secondary structure near positive charge clusters are unable to explain the pausing after translation of positive charges (Figure S14). Hence we argue that mRNA folding cannot explain the slowing seen in Figure 5, which is perhaps not surprising given its apparently weak effect on the whole (Figure 4).

### Covariance with Positive Charge Does Explain Some of the Slowing Observed After RNA-Level Features

Given that positive charge slows ribosomes, we should expect that some of the (relatively weaker and/or inconsistent) ribosomal slowing at rare codon clusters or transcript secondary structure might in fact be due to the presence of uncontrolled-for positive charge. We find this to be the case. When groups of rare codons that are followed by either a lesser or greater number of positive charges are plotted separately, it is clear that rare codon clusters do not in and of themselves slow ribosomes (Figure 2B) but that the apparent (yet unsystematic) slowing in Figure 2A is in fact due to the presence of positive charge after some of the codon clusters (Figure 2C). Similarly, sorting by the number of positive charges present after a cluster reveals that some of the slowing observed at structured regions of transcript is likely due to previously unaccounted-for positive charge (Figure 4).

## Discussion

We find that codon usage and transcript secondary structure do not substantially affect ribosomal velocities systematically across endogenously occurring transcripts. Although it has been suggested that amino acid starvation might increase the ability of codon usage to modulate ribosomal speed [37], we find no such effect upon examination of ribosomal footprints taken from amino-acid-starved yeast (Figures S8, S9, S10 and Table S5). We do not, however, wish to assert that codon usage and RNA structure can never affect translation rates. Certain secondary structure configurations may substantially impact ribosomal flow. As regards codon usage, if we return to the original logic by which codon usage was thought to affect translation rates, we can both see where the prior logic was misleading and in turn can predict when codon usage should slow ribosomes.

The classical logic supposes that because common codons are specified by abundant tRNAs, the waiting time for the ribosome to capture the necessary tRNA must be lower for “optimal” or common codons. The key parameter, however, to determine waiting time is not the absolute tRNA abundance (as often considered) but the tRNA availability. We note, similarly to Qian et al. [16], that if codons are used in proportion to tRNA availability [40], then this could dampen any pausing effect, since rare codons matching rare tRNAs will not be as rate-limiting as if they were used more often. Put differently, if highly abundant transcripts all require the same tRNA, then this acts as a drain on the availability of that tRNA. This can be described in terms of supply and demand economics. In the case of rare codons in lowly expressed transcripts, the supply (the pool of tRNA) is small and the demand (number of codons requiring that tRNA at any given time) low. For a common codon in an abundant transcript, the supply (tRNA pool) is large but the demand is also large.

We can then imagine an equilibrium situation in which the ribosome waiting time is the same for all codons as the demand (absolute codon abundance in transcripts) and supply of tRNAs are balanced. This is consistent with our observation that, under normal growth conditions, codon usage does not predict ribosome occupancy. However, the same model can predict that under abnormal conditions, we might see an effect as the situation has been forced far out of supply–demand equilibrium. Greatly overexpressing a transcript rich in rarely used codons should slow the ribosome as the demand for the rare tRNAs now exceeds supply. Likewise, we expect that gross modification of tRNA pools should have gross effects on translational speed as the system has been shifted away from the demand–supply equilibrium. This distinction between normal (equilibrium) and experimentally forced (nonequilibrium) conditions makes good sense of the prior literature, where reports of an effect of codon usage on translational velocity involved experimentally forced conditions (for review, see Note S1).

Further evidence that the impact of codon/tRNA abundance is buffered comes from the report that some codons whose aminoacyl-tRNAs are selected either intrinsically rapidly or slowly by the ribosome have either low or high tRNA concentrations within the cell, respectively [41], suggesting that intrinsic differences in the translation speeds of certain codons are not accentuated but rather compensated for. The evidence for codon usage/tRNA buffering indirectly suggests either that some property other than speed causes selection on codon usage (e.g., accuracy of translation [18]–[21]) or that selection for speed occurs when the demand–supply balance is perturbed, for example when selection acts on growth rates and favor duplications of tRNAs. That codon usage also has little or no effect on ribosome velocity in mammals [15] as well as yeast is then, in retrospect, perhaps not so unexpected.

Our results are consistent with the interaction of the cations in the protein with the ribosomal exit tunnel [25],[26], a model supported by the stalling being displaced from the location on the mRNA of the codons specifying the positive charge. Our results also indicate that positive charge, more than other chemical or biophysical properties of amino acids (see Tables S6, S7, S8, S9), is key. While some highly conserved amino acid sequences have been shown to interact with the ribosomal tunnel to stall translation in order to regulate the specific gene product they control (see, e.g., [42]–[44]), our results suggest a fundamental feature of proteins that slows ribosomes regardless of sequence context (either the local amino acid sequence or the gene in which they reside) and without the addition of trans acting factors.

A general slowing of translation due to positive charge has ramifications for the evolution of the poly-A tail. If translated, the poly-A tails results in a long run of positively charged lysines. This is expected to stall run-on ribosomes [39]. This stalling may glue the aberrantly translated peptide to the ribosome, preventing potentially toxic products from diffusing into the cell and/or permit tagging of the peptide in the nascent chain–ribosome complex with a signal for degradation, as observed [26],[39].

Our results are consistent with translation of poly-A tails stalling ribosomes. Extrapolating the linear trend for larger clusters of positive charges to additively slow ribosomes (reported in Figure 3C), we note that a poly-A tail of 80 consecutive adenines (∼27 lysines) in yeast [45] should slow translation at least 4-fold more than that observed in clusters of six or more positive charges (Figure 3C), probably halting it. This is in line with experimental work showing that while nonstop mRNAs without poly-A tails are efficiently translated [46], translation of polyadenylated mRNAs lacking stop codons or full 3′UTRs is repressed after initiation [47]. Similarly, inserting a poly-A tract into a coding sequence represses translation post-initiation, but not on account of rapid mRNA decay [39]; a similar finding was reported for 3′ poly-A tails [48]. Recently, it was shown that translation of 12 consecutive basic amino acids inserted into a reporter gene causes not only translation arrest but degradation of the polypeptide [49].

Why is the tail poly-lysine if any positive charge will do? The reason is likely to be found at the DNA sequence level. Of all codons encoding positive charges, only lysine possesses a codon that is a triplet repeat of a single nucleotide (AAA) and therefore may be added simply and sequentially by a single enzyme. Moreover, the triplet repeats form a homogenous run of adenines, meaning that positive charges will still be added to the nascent chain (and hence stall ribosomes) no matter how the stop codon is missed, be it by failure to interpret the stop when in-frame or owing to frame-shifting. This may have less relevance in species with long 3′UTRs, in which an alternative stop may be found with the UTR, but in the ancestor in which the poly-A tail evolved, if 3′UTRs were short, then this sandtrap for ribosomes may have been of considerable benefit.

It is noteworthy that bacteria, which for the most part lack poly-A tails, have an alternative mechanism (tmRNA) to tag and destroy proteins resulting from frameshifting or stop codon readthrough [50]. Stalling initiated by positive charges resulting from translation of poly-A tails in eukaryotes and tmRNA system in prokaryotes may be functionally equivalent modes of error correction [51].

## Methods

### Ribosomal Density Data

Both sequenced ribosomally protected fragments and sequenced fragmented total mRNA for *S. cerevisiae* dataset GSE13750 [29] were downloaded from the NCBI Gene Expression Omnibus at www.ncbi.nlm.nih.gov/projects/geo. The rich media and amino-acid-starved sets were considered separately. Annotations of the *S. cerevisiae* S288C genome as available on June 22, 2008 (the build used by Ingolia et al. [29]) were obtained from the *Saccharomyces cerevisiae* Genome Database (www.yeastgenome.org). Only protein-coding sequences of nondubious classification were considered, giving 6,262 genes for potential analysis. Any sequences containing nonsense codons or that were not multiples of three were excluded. The sequences were further filtered to only allow the standard or alternative start codons indicated in NCBI genetic code Table 1 from http://www.ncbi.nlm.nih.gov/Taxonomy/taxonomyhome.html/index.cgi?chapter=tgencodes, leaving 6,215 sequences for analysis. The chromosomal location and coordinates of the sequenced fragments given in the original dataset were used in combination with the start and stop coordinates of genes from the annotations to determine which fragments map to which genes, and in the case of the footprint fragments, where along the coding sequence the protected area lies.

Since the probability of sequencing error in a stretch of ∼28 nucleotides is quite low (for runs <50 bp on Genome Analyzer 2, error rates are expected to be around 1%), only one mismatch between the sequenced fragment and reference genome sequence was allowed. All fragment counts were taken as the average value of the two experimental replicates. Fragments that were sequenced at least once in one replicate but not listed in the other were marked as having an expression count of 0 at analogous positions in the latter replicate. In the case of fragments that map to more than one possible genomic location, it is impossible to tell which are the true areas covered by ribosomes. In order to avoid the introduction of false-positive ribosomal occupancies en masse into the dataset, which could systematically bias the types of sequences that are occluded, only footprints that mapped uniquely to one location in the reference genome were considered.

In line with Ingolia et al. [29], we assigned footprints to protein-coding genes of nondubious classification if the first base of the footprint mapped to 16 nt before the first base or 14 nt before the last base of the gene, in order to take account of which area of the footprint is likely in the ribosomal active site. Since the chance of sequencing another fragment from a stretch of coding sequence increases as a function of gene length, mRNA fragment counts were normalized by dividing by gene length for the relevant gene. In addition, the footprint counts were then divided by the normalized mRNA counts mapping to that gene to obtain per-transcript ribosomal densities (indexed by location along the transcript). We performed this normalization by mRNA to ensure that differences in occupancies we calculate (see Methods, “The Average Effect of Positive Charge on Ribosomal Densities”) are not an artefact of mRNA levels. This left us with a final 5,430 filtered genes with footprint coverage mapped per codon pair per transcript.

### The Statistical Approach to Handling the Occupancy Data

We note that there are two previous studies [27],[28] that examined this ribosomal footprint data [29] and found a role for codon usage in modulating ribosomal speeds, mainly by detecting a correlation between the local codon optimality along a transcript and the corresponding local density of ribosomal footprints. We cannot offer a reason why these studies produce such a finding, namely because they do not detail their methodology concerning the ribosomal footprint data, including whether they used all or just a subset of all the sequenced footprints (e.g., dependent on footprint length, the number of mismatches to the genome reference sequence allowed, or the number of places in the genome to which a single footprint could simultaneously map). Another study [16] that examined the same data contradicted the finding that codon usage affects ribosome velocity, highlighting the importance of methodology in the analysis of ribosomal profiling data. This opposing study [16], however, may have mapped footprints to multiple genomic locations and also considered only footprints 28 nt in length in an attempt to precisely map which codon is in the A-site and hence selecting an aminoacylated tRNA. We are not confident that the interpretation of ribosomal footprint data allows for such specificity, as the interpretation of where the A- and P-sites are along the ribosomal footprint seems to be inferred from the average footprint length obtained during initiation and termination [29], whereas the conformation of the ribosome and hence footprint obtained during elongation may differ. Also, it has since been noted that elongation inhibitors, such as cyclohexamide, which was used in the creation of the dataset under consideration [29], alter the conformation of the ribosome, leading to advised caution in determining position-specificity from individual footprints [15]. For these reasons, we consider it optimal to stringently map footprints to a single location in the genome, thus preventing the introduction of a false correlation between certain codons and ribosomal density, and to consider all of the sequence that is occluded by the footprint instead of attempting to pinpoint the location of a structural site in the ribosome from the artefact of the footprint.

To determine what determines occupancy, we could consider some general linear model in which we employ multiple parameters (local codon usage, local RNA stability, and local charge density) to predict occupancy on a codon-by-codon basis. However, such models assume that the data points are independent. Owing to the nature of the data (ribosomes sit over spans of sequence), the occupancy seen at one codon by necessity is nonindependent of that seen at neighboring codons. Thus, such methods are not generally valid. To overcome the nonindependence problem, we do not consider each codon as a separate data point. Rather we consider the dimensions of the spans of increased relative occupancy and consider how trends in the dimensions of these spans correlate with the density of the potentially slowing feature in question, an approach that we outline in Figure 1 and below.

We consider for any feature (e.g., a cluster of codons or positive charges) the start position of this feature (position *x* = 0). We then define for each codon at and after the start of the feature (*x*≥0) how the occupancy is related to the mean occupancy of the 30 codons upstream of *x* = 0 within the same mRNA as the feature. We define the relative occupancy of any given codon (*r_pos_*/*r_prec30_*) as its occupancy (*r_pos_*) divided by the mean occupancy of the 30 codons prior to the considered feature (*r_prec30_*). For plotting purposes, we also normalize the occupancy of all codons 5′ of the focal position at *x* = 0 by the same *r_prec30_* value. Dividing by pre-cluster ribosomal densities to obtain a ratio normalizes for differences between transcripts such as expression level, accommodates and normalizes for differences in ribosomal density that may be caused by characteristics of upstream sequence, and allows for comparisons of the relative change in ribosomal movement across different mRNAs. These relative occupancy ratios, which we calculate surrounding every identified feature, thus represent the speeding (if the ratio is <1) or slowing (if the ratio is >1) of ribosomes after a given feature as they translate that portion of that gene. After calculating the relative ribosomal occupancy ratios surrounding a feature within all available mRNAs, we then align these mRNAs by the start of that feature and calculate the average relative occupancy ratios across transcripts. We plot these averages such that *y* at codon *x* = 1 is the mean ratio of all the observations across multiple RNAs at *x* = 1, codon *x* = 2 is the mean ratio across multiple RNAs at *x* = 2, and so on. These plots then present a span of increased relative occupancy or of decreased relative occupancy following the start of the feature at *x* = 0 across all instances of that feature available to our analysis.

### The Average Effect of Codon Usage on Ribosomal Densities

As tRNA gene copy number has been shown to strongly correlate with tRNA abundance [27],[52], preferential use of codons that base-pair to the anticodons of high-copy tRNAs is taken to reflect adaptation of coding sequence to the tRNA pool and hence optimal codon usage for translational efficiency and/or accuracy. Each codon can then be ascribed an adaptiveness value (*W_i_*) [32]. The tRNA adaptation index (tAI) is the geometric mean of the scores for the constituent codons and is then a measure of the degree to which protein-coding genes use codons corresponding to tRNA isoacceptors with high gene copy numbers within a given genome (although, see also Figures S1, S2, S3 and Table S1 for analyses of rare codons where “rare” is defined as genomically infrequent) [32]. The codonR package to calculate tAI was downloaded from http://people.cryst.bbk.ac.uk/~fdosr01/tAI/index.html on May 7, 2011. Yeast tRNA genes were obtained from the UCSC Table Browser [53] at http://genome.ucsc.edu/cgi-bin/hgTables. Statistical calculations for the tAI (and for other analyses generally) were done in R [54].

As a gene with just one codon would have a tAI value equal to *W_i_* of that codon, we refer in the text to a codon's tAI value. In the main text we define rare codons to be those in the lowest quartile of *W_i_* values as derived for yeast (CGA, ATA, CTT, CTG, CTC, CGG, AGT, CCC, GCG, AGC, CCT, TCG, TGT, ACG, and GTG). We interchangeably use “rare” for “non-optimal” as the frequency of codon usage in yeast is roughly proportional to the numbers of tRNAs that can decode them [40]. Protein-coding sequences were scanned for single rare codons; two rare codons anywhere within a five-codon stretch, three rare codons within eight codons, four or five within 10, and six or more within 16. The cluster specifications outlined here were chosen to maximize cluster sample sizes while incorporating the following caveat: we required that a block of 30 non-rare codons had to precede the identified codon clusters and that no other rare codons could be present in the next 30 codons apart from those in the identified cluster. When investigating consecutive rare codon clusters in a parallel analysis, we required that no consecutive rare codons could be present in the surrounding ∼60 codons apart from those in the identified cluster (single rare codons were permitted as otherwise sample sizes would be far too small). As noted above, the first rare codon in the cluster is always considered to be at position *x* = 0.

As there are not enough rare codon clusters that are isolated from the ribosome-slowing effects of positive charges, we were unable to introduce the requirement that no positive charges be present in the vicinity of the rare codon cluster. Instead, we split the rare codon clusters into two groups—those that had two or more positive charges coded for in the sequence following the rare cluster, and those that had either zero or one positive charge—and plotted the results for these groups separately.

As noted above, we perform a normalization with respect to the local ribosomal occupancy. Within a given mRNA, the relative increase or decrease in ribosomal density (*r_pos_*/*r_prec30_*) at each position surrounding a rare cluster was calculated by dividing the measured ribosomal density at each codon position (*r_pos_*) by the average ribosomal occupancy of the thirty codons preceding the first rare codon in the cluster (at position *x* = 0) within that same mRNA (*r_prec30_*). The average relative change in ribosomal occupancy (mean *r_pos_*/*r_prec30_*) at a given position during/after a cluster was then calculated by aligning all identified regions of a given cluster size according to the first codon present in each cluster and calculating the average ratio (i.e., increase or decrease in measured ribosomal occupancy) in positions increasingly distant from the aligned clusters (a schematic of this approach is contained in Figure 1).

### The Average Effect of Transcript Structure on Ribosomal Densities

We used experimentally and not computationally determined RNA structure data. By exposing transcripts independently to endonucleases specific for single- and double-stranded RNA, the degree to which individual nucleotides of an mRNA are involved in intramolecular secondary structure has been experimentally quantified [38]. The resulting metric is “parallel analysis of RNA structure” (PARS) values, with higher values (positive) indicating a propensity for secondary structure and lower (negative) values signifying lack thereof. The authors show the PARS metrics along transcripts in yeast globally correlate with the degree of single- versus double-strandedness predicted by the Vienna Package. PARS values for yeast transcripts were downloaded at http://genie.weizmann.ac.il/pubs/PARS10/pars10_catalogs.html. PARS values corresponding to CDS regions were determined relative to the local coordinate file from the same website.

*S. cerevisiae* protein-coding sequences were scanned for stretches 30 codons in length whose average PARS value was 0 or negative (and hence tending to be single-stranded), which were immediately followed by a block 31 codons in length whose average PARS value was positive (i.e., with propensity for double-strandedness). To ensure a clear transition from single- to double-stranded structure upon averaging across transcripts, we added the requirement that the last codon in the first 30-block have a negative PARS value and that the first codon in the subsequent 31-block have a positive PARS value. Only nonoverlapping blocks (61 codons in length) were retained, with priority given to those with the highest combined number of negative PARS value in the first 30 codons and positive PARS value in the latter 31 codons.

The general contribution of folding to slowing was then examined by calculating *r_pos_*/*r_prec30_* (as described above) at each position and then taking the average across aligned single-stranded into double-stranded blocks. In the first instance of such a test, we investigated the hypothesis that the ribosome closely approaches the base of the double-stranded structure such that the ribosome is positioned closely over the first double-stranded ribonucleotide (i.e., at the beginning of the 31st codon out of 61) by the time slowing occurs. Here the first 30 codons in the identified block are classed as the preceding 30 codons before slowing might occur. We then repeated the analysis examining whether pausing of the ribosome might occur somewhat further upstream—for example, if the mass of the ribosome sterically hinders it from progressing at its normal rate even before the double-stranded ribonucleotide approaches the active site. In this second analysis, we used the same identified blocks as above, but moved the potential point of slowing to 10 codons upstream of the first codon with a positive PARS score. Hence the preceding 30 codons used in this case to normalize nearby ribosomal densities were also shifted 10 codons upstream as well.

### The Average Effect of Positive Charge on Ribosomal Densities

Changes in rates of translation were measured by calculating the relative change in ribosomal densities that occurs within a transcript, on average, after positively charged residues (lysine, arginine, or histidine) are added to the nascent peptide chain. Such an effect should be observed at or after the encoded charge(s) in the mRNA as the positively charged amino acid travels down the exit tunnel. To test for an additive effect of charge on ribosomal density, *S. cerevisiae* protein-coding sequences were scanned for single positively charged amino acids, two positively charged residues anywhere within five amino acids, three positively charged residues within eight amino acids, four or five positively charged amino acids within 10 amino acids, and six or more positive charges within 16 amino acids, with the first positively charged residue always considered to be at *x* = 0. As in the case of the rare codon cluster analyses, these loosely defined cluster specifications were chosen to maximize the sample sizes available of clusters containing different numbers of positive charges.

To eliminate interference from charged amino acids outside these charged clusters, we required that a block of 30 non-positively charged amino acids precede the identified positive-charge clusters, and that no other positively charged amino acids be present in the next 30 amino acids apart from those in the identified cluster. Thirty residues were chosen as this is approximately the length of extended peptide that the ribosomal exit tunnel can accommodate [55],[56]. Thirty non-basic residues therefore should provide a baseline ribosomal occupancy reading, and hence inference of the speed of translation, before the positively charged residues are added to the peptide chain and enter the exit tunnel.

The relative increase or decrease in ribosomal density at each position (*r_pos_*/*r_prec30_*) was calculated for each transcript with an encoded positive-charge cluster. The average relative change in ribosomal occupancy (mean *r_pos_*/*r_prec30_*) at a given position during/after a cluster was then calculated across regions aligned by similar-sized clusters (see also Figure 1 for a visual of this approach). Regarding our methodology, we find that noise in footprint density is not a problem for our analysis as we see similar findings when we consider genes with either low or high footprint coverage (Figure S15).

### The Relative Contributions of Charge, Folding, and Codon Usage to Extremes of Slowing Within Transcripts

The above methods start by locating the appropriate putative ribosome-slowing feature within transcripts and then measures changes in ribosomal occupancy surrounding them. A complementary approach is to look to large changes in ribosomal density and then ask whether positive charges or rare codons are more often associated with the denser, putatively more slowly translated regions. Such an approach is best carried out on a within-mRNA level, as this normalizes for differences in overall expression levels across genes. Within each gene for which we retained ribosomal protection data (see Methods, “Ribosomal Density Data”), we located the two nonoverlapping 10-codon windows (approximately the length of RNA a ribosome footprint spans [29]) with the highest and lowest average ribosomal occupancy in that transcript. To circumvent the arbitrariness of choosing the location of the low-occupancy 10-codon window in a transcript for which there may be multiple possible windows with no footprint data available (i.e., a footprint count of 0), we added the requirement that experimental protection data exist for each codon in the window.

For each window in the pair, we recorded the average ribosomal occupancy as well as (1) the tAI; (2) the number of adjacent, nonoverlapping pairs of rare codons; (3) the number of positive charges encoded within and up to five codons upstream of the window (since a charge added while the ribosome was a few codons upstream should still be present within the exit tunnel); (4) the number of rare 6-mers (defined to be the lowest 10% of all possible in-frame 6-nt sequences within open reading frames); and (5) propensity for transcript secondary structure. We included 6-mers here, as while individual codons may be rare, it does not necessarily follow that two adjacent rare codons are just as rare of a combination, and thus examining the contribution of rare 6-mers provides an extra level of stringency in assessing the role of codon usage. As secondary structure either at the codon in question or downstream of the codon in question might pause ribosomes (see Results), we considered the PARS values not only within but also for an additional 10 codons downstream of each originally identified 10-codon window.

If codon usage bias is modulating ribosomal speed, we expect to observe the main effect over and locally surrounding the codon in question, whereas we expect, if charge indeed is influencing ribosomal velocity, to observe a downstream effect of positive charge on ribosomal density as the cation travels further down the negatively charged exit tunnel. Thus we also counted any positive charges encoded in the five codons immediately preceding the identified windows since a charge added while the ribosome was a few codons upstream should still be present within the exit tunnel. Conversely, as secondary structure either at the codon in question or downstream of the codon in question might pause ribosomes (see Results), we considered the PARS values not only within but also for an additional 10 codons downstream of each originally identified 10-codon window. Since the interpretation of PARS values may be somewhat more labile (as not only the magnitude but the sign of the values may have meaning), we tested whether double-stranded structure might associate with the more dense window in two different ways. We used two methods of measuring propensity for transcript structure. In the first method (“PARS score”), an average PARS value for a window ≤0 means the window is single-stranded, and an average PARS value >0 means the window is double-stranded; the exact magnitude of the PARS value is disregarded beyond this. In the second method (“conservative PARS score”), smaller changes in the magnitude of PARS values count more: the mean PARS value is calculated for each extended (20-codon) window, and the means are then compared.

The ability of each metric to explain the difference in average ribosomal occupancies between the two windows was then assessed by asking how often the window with more of a potentially ribosome-slowing feature was also the window with greater occupancy. For example, if the window with the higher ribosomal occupancy paired with the less optimal (lower) tAI—which would be expected if less optimal codons do in fact slow ribosomes—then the gene was assigned a tAI score of 1; if the higher occupancy window paired with the more optimal tAI, indicating tAI is not a good predictor of increased occupancy, the a tAI score of −1 was assigned; and if the tAI was the same in the two windows, a score of 0 was given. Similar tests were performed independently on the number of rare codon pairs, PARS metrics, and number of positive charges associated with each window, with more rare pairs/more positive charges in the more occupied window—and hence potentially capable of explaining the elevated ribosomal density—each being scored 1, fewer being scored −1, and the same number in each window scored 0.

There are two potential complications of this method that we are able to address and dismiss. Firstly, although there is a tendency for ribosomal occupancy to decrease, on average, along the length of transcript [29], the correlations we report hold when we test only transcripts in which the high ribosomal occupancy windows are downstream of the low ribosomal occupancy windows (higher occupancy and increased positive charge, Spearman rho 0.12, *p* = 0.00031; higher occupancy and an excess of rare pairs, Spearman *p* = 0.62; an excess of rare 6-mers, rho = −0.06, *p* = 0.07; higher occupancy and lower tAI, Spearman rho −0.15, *p* = 2.4e-05). This, along with the additive pattern in Figure 3C, shows that the correlation between positive charge and increased ribosomal density is not methodological artefact.

Secondly, a window might have an apparently low average ribosomal occupancy if in fact there were ribosomal footprints that should have been assigned to that region of the transcript, but which was ultimately excluded from the analysis if the footprint mapped to multiple genomic locations. To this we note that the same analysis, when redone allowing the low-occupancy window to have a footprint count of zero, still gives similar results (Table S13). To further test this possibility, we created a list of nonredundant locations. These are sites in each transcript for which all mapping footprints were uniquely mapping to that location. In other words, no footprint data were excluded from being mapped to these sites because it also mapped somewhere else in the genome. Redoing the window comparisons analysis using the nonredundant locations, we find the results (Figures S16, S17 and Table S14) qualitatively match our original results using the dataset described above (see Methods, “Ribosomal Density Data”). Hence, we consider that our method fairly infers the contribution of different sequence features to ribosomal slowing.

## Supporting Information

Figure S1 Figure 2 redone using rare codons defined according to genomic frequency shows rare codons do not slow ribosomes. In the main text, we investigate whether nonoptimal codons—that is, those with low tAI scores—might slow codons and find that they do not. To ensure that our finding that these “rare” codons do not slow ribosomes does not simply hinge on our definition of “rare,” we have repeated the analysis using an alternative definition. Here, we define “rare” codons according to their actual frequency in the genome as measured from our set of filtered genes. This rare set, of equal size to the rare tAI set, comprises the following codons: CGG, CGC, CGA, TGC, CCG, CTC, GGG, GCG, CGT, CCC, CAC, TGT, ACG, TCG, and AGG. We find that rare codons, where rare means genomically rare, do not slow ribosomes when in clusters (single rare codons; two rare codons anywhere within a five-codon stretch; three rare codons within eight codons; four or five within 10; and six or more within 16). Note slowing should be observed over, not after, the rare codon(s). (A) All genes with rare codon clusters. Regression of *area under curve*∼*number of rare codons in cluster*, slope = −0.79, *p* = 0.080. Regressions were performed as detailed in the main text (see Figure 1 for a description of the calculation of area under the curve). We note even if *p* were significant, the slope would be negative, whereas if rare codons did slow ribosomes, we should expect to see a positive slope. (B) Genes with rare codon clusters that have 0 or 1 positive charge coded for in the last 30 codon positions plotted. These plots represent the net effect of tAI on ribosomal density, with the bulk of the effect of positive charge removed. (C) Genes with rare codon clusters that have two or more positive charges in the last 30 codon positions plotted.
(PDF)

Figure S2 Consecutive rare codons, where rare is with reference to genomic frequency, do not slow ribosomes. In the main text, we investigate whether nonoptimal codons—that is, those with low tAI scores—might slow codons and find that they do not. To ensure that our finding that these “rare” codons do not slow ribosomes does not simply hinge on our definition of “rare,” we have repeated the analysis using an alternative definition. Here, we define “rare” codons according to their actual frequency in the genome as measured from our set of filtered genes. This rare set, of equal size to the rare tAI set, comprises the following codons: CGG, CGC, CGA, TGC, CCG, CTC, GGG, GCG, CGT, CCC, CAC, TGT, ACG, TCG, and AGG. The consecutive rare codons in considered codons are present between the first and second arrowheads. See Figure 1 for a description of the calculation of the area under the curve. (A) All genes with rare codon clusters. Regression of *area under curve*∼*number of rare codons in cluster*, slope = −9.8, *p* = 0.32. (B) Genes with rare codon clusters that have 0 or 1 positive charge coded for in the last 30 codon positions plotted. These plots represent the net effect of tAI on ribosomal density, with the bulk of the effect of positive charge removed. (C) Genes with rare codon clusters that have two or more positive charges in the last 30 plotted codon positions.
(PDF)

Figure S3 Codons that are overused in high-ribosomal occupancy windows are not “rare” according to genomic frequency. In some supplemental analyses, we examine whether “rare” codons slow ribosomes and define “rare” as the quartile of those most infrequent codons in the genome. To ensure there is not a problem with this definition, we have examined the difference in trends of codon usage at large between the two windows. (A) Tallies of all the codons used among the high-occupancy and low-occupancy windows within each gene (including the preceding five codons before each window) were kept separately. We plotted the counts for each codon in the high ribosomal occupancy window versus the counts in the low occupancy window and have color-coded the codons according to their frequency (see also Figure S6 for rare codons defined according to their tAI). If all codons are used equally among the slowly translated and quickly translated windows, then the regression should give a slope of 1, with all data points falling precisely upon the regression line. Since we have no prior expectation as to which variable should be on the *x-* versus *y*-axis—we are simply testing for a slope of 1—we used standardized major axis regression using the “smatr” package in R. We performed standardized major axis regressions of *usage count(codon)*, *high occupancy windows*∼*usage count(codon)*, *low occupancy windows* along with package tests that the slope of the line is 1 and that the intercept falls through 0. When we consider only those codons within the lowest quartile of frequency values, we find that the resulting regression has a slope not significantly different from 1 (*p* = 0.51) and an intercept not significantly different from 0 (*p* = 0.68), indicating that on the whole the rarest (tAI) quartile of codons are used equally between the slow and quickly translated windows. Considering all codons, however, gives a regression with both a slope different from 1 (*p* = 2.9e-04) and an intercept different from 0 (*p* = 4.4e-04), corroborating that not rarer but more common codons are used more in the high-occupancy windows. The line *x = y* is plotted just as a visual aid. (B) An examination of the residuals from (A). Those codons that lie more than ∼2 standard deviations away from the regression line are not from the rare end of the frequency spectrum but do tend to encode positively charged residues. Horizontals at *y* = −1.96, +1.96 are plotted. (C) Given that there will of course be constraints on amino acid sequence, we also desire to investigate the differences in codon usage between the two windows given the protein-coding composition of each. All of the total codon counts for each low-occupancy window (as described above) were divided by the total amino acid count encoded by that codon for the low-occupancy window. The same normalization was performed for the high-occupancy windows, and the normalized codon counts were then plotted against one another. Performing a standard major axis regression on the amino acid-adjusted codon counts shows that codons, given the protein coding sequence, are on the whole used proportionally between the quickly and slowly translated windows. When we consider only those codons within the lowest quartile of frequency values, we find that the resulting regression has a slope not significantly different from 1 (*p* = 0.74) and an intercept not significantly different from 0 (*p* = 0.25), indicating that on the whole the rarest (frequency) quartile of codons are used equally between the slow and quickly translated windows. Considering all codons, we find a slope significantly different from, but very close to, 1 (*p* = 0.049; slope 95% CI of 1.00, 1.08) and an intercept not different from 0 (*p* = 0.10). The line *x = y* is plotted as a visual aid. (D) The finding in (C) that codons, on the whole, are not used significantly different between the slowly and quickly translated windows (given their respective amino acid compositions) is confirmed by an analysis of the residuals. The one codon that is possibly significantly overused does not have a low genomic frequency. Horizontals at *y* = −1.96, +1.96 are plotted.
(PDF)

Figure S4 Shifting the “preceding 30 codons” window 4 codons upstream to accommodate the “back” of the ribosome still shows rare codons do not slow ribosomes. Imagining ribosomes did stop at rare (tAI) codons, the A-site would still be ∼10–12 nucleotides from the end of the ribosomal footprint. To make sure we are not in fact improperly normalizing footprint counts around rare clusters by a “preceding 30” sequence that contains part of the footprints, we moved the “preceding 30 codons” window upstream by four codons (i.e., 12 nt). We achieve very similar results to those presented in the main text (see Figure 2). (A) All genes with rare codon clusters. (B) Genes with rare codon clusters that have 0 or 1 positive charge coded for in the last 30 codon positions plotted. These plots represent the net effect of tAI on ribosomal density, with the bulk of the effect of positive charge removed. (C) Genes with rare codon clusters that have two or more positive charges in the last 30 codon positions plotted.
(PDF)

Figure S5 Pairs, triplets, etc. of rare (low tAI) codons do not tend to slow ribosomes. The consecutive rare codons in considered codons are present between the first and second arrowheads. The mean *r_pos_/r_prec30_*, or relative change in ribosomal occupancy, at each position across aligned transcripts ± s.e.m. is plotted. The horizontal at *y* = 1 represents the null expectation that positive charges do not alter ribosomal speed—that is, that ribosomes are, on average, as frequently present before the rare codon cluster as after it. (A) All genes with rare codon clusters. (B) Genes with rare codon clusters that have 0 or 1 positive charge coded for in the last 30 codon positions plotted. These plots represent the net effect of tAI on ribosomal density, with the bulk of the effect of positive charges removed. (C) Genes with rare codon clusters that have two or more positive charges in the last 30 plotted codon positions.
(PDF)

Figure S6 Codons that are overused in high-ribosomal occupancy windows are not “rare” according to tAI. In the main text, we examine whether “rare” codons slow ribosomes and define “rare” as the lowest quartile of tAI values within the genome. To ensure there is not a problem with this definition, we have examined the difference in trends of codon usage at large between the two windows. (A) Tallies of all the codons used among the high-occupancy and low-occupancy windows within each gene (including the preceding five codons before each window) were kept separately. We plotted the natural log of counts for each codon in the high ribosomal occupancy window versus the natural log of counts in the low occupancy window and have color coded the codons according to their tAI (see also Figure S3 for rare codons defined according to their genomic frequency). If all codons are used equally among the slowly translated and quickly translated windows, then the regression should give a slope of 1, with all data points falling precisely upon the regression line. Since we have no prior expectation as to which variable should be on the *x-* vs. *y*-axis—we are simply testing for a slope of 1—we used standardized major axis regression using the “smatr” package in R. We performed standardized major axis regressions of *usage count(codon)*, *high occupancy windows*∼*usage count(codon)*, *low occupancy windows* along with package tests that the slope of the line is 1 and that the intercept falls through 0. When we consider only those codons within the lowest quartile of tAI values, we find that the resulting regression has a slope not significantly different from 1 (*p* = 0.93) and an intercept not significantly different from 0 (*p* = 0.82), indicating that on the whole the rarest (tAI) quartile of codons are used equally between the slow and quickly translated windows. Considering all codons, however, gives a regression with both a slope different from 1 (*p* = 4.0e-04) and an intercept different from 0 (*p* = 5.5e-04), corroborating that not rarer but more common codons are used more in the high-occupancy windows. The line *x = y* is plotted just as a visual aid. (B) An examination of the residuals from (A). Those codons that lie closest to ∼2 standard deviations away from the regression line tend to encode positively charged amino acids. Horizontals at *y* = −1.96, +1.96 are plotted. (C) Given that there will of course be constraints on amino acid sequence, we also desire to investigate the differences in codon usage between the two windows given the protein-coding composition of each. All of the total codon counts for each low-occupancy window (as described above) were divided by the total amino acid count encoded by that codon for the low-occupancy window. The same normalization was performed for the high-occupancy windows, and the normalized codon counts were then plotted against one another. Performing a standard major axis regression on the amino-acid-adjusted codon counts shows that codons, given the protein coding sequence, are on the whole used proportionally between the quickly and slowly translated windows. When we consider only those codons within the lowest quartile of tAI values, we find that the resulting regression has a slope not significantly different from 1 (*p* = 0.45) and an intercept not significantly different from 0 (*p* = 0.89), indicating that on the whole the rarest (tAI) quartile of codons are used equally between the slow and quickly translated windows. Considering all codons, we find a slope significantly different from, yet very close to 1 (*p* = 0.032; slope 95% CI of 1.00, 1.10) and an intercept again not different from 0 (*p* = 0.07; intercept 95% CI of −0.034, 0.0015). The line *x = y* is plotted as a visual aid. (D) The finding in (C) that codons, on the whole, are not used significantly differently between the slowly and quickly translated windows (given their respective amino acid compositions) is confirmed by an analysis of the residuals. The one codon that is possibly significantly overused does not have a low tAI value. Horizontals at *y* = −1.96, +1.96 are plotted.
(PDF)

Figure S7 Similarity to Kozak sequence is not the primary cause of ribosomal slowing. Given that transcript similarity to the Shine-Dalgarno sequence has been shown to slow ribosomes in bacteria due to interactions of the sequence with components of the ribosomal RNA [17], we wondered whether translation speed in yeast might not be modulated by codon usage per se but by the ability of ribosomes to bind to transcript sequence that mirrors the eukaryotic Kozak sequence. Specifically, we wanted to determine whether codons that are in high-ribosomal occupancy windows within a gene might be more likely to correspond to the Kozak sequence (as compared to codons in low-occupancy windows within the same genes) and hence bind ribosomes, slowing translation. We first determined which codons were enriched in the Kozak sequence relative to the codon frequencies seen throughout the yeast genome at large using a simple randomization. Nucleotide frequencies at each position of the Kozak sequence in yeast were taken from Cavener and Ray 1991 [57]. To determine the frequencies of all the possible “codons” among the Kozak sequence space, we randomly created 20,000 possible Kozak sequences from the delineated nucleotide frequencies at each site in the consensus sequence. We then counted all possible triplet “codons” within each sequence, regardless of reading frame (since we assume that as the ribosome traverses RNA, it may bind the Kozak sequence regardless of the surrounding reading frame). The counts of all possible RNA triplets that we observe within our simulated sequences are the observed “codons” within the Kozak sequence. In order to determine whether or not certain codons are over- or underused in the Kozak sequence, we compare them to the counts of codons observed (again in any reading frame) across 20,000 randomized sequences derived from the basal codon frequencies in the *S. cerevisiae* genome and of the same length as the Kozak sequence. We calculate *Z*, a measure of the over- or underusage of a particular codon within the Kozak sequence (as compared to the rest of the genome) as *Z*_codon_ = [Observed codon count (in Kozak sequence) – Expected count (from genome frequencies)]/Expected SD of codon. We can then examine which codons are overused (i.e., with a positive *Z*-score) in slowly translated windows relative to quickly translated windows in the same genes and ask if these codons are overrepresented among the Kozak sequence(s). If so, this would suggest that RNA sequence may be slowing ribosomes not through codon–anticodon interactions but by Kozak-similar sequences binding the ribosome. (A) Tallies of all the codons used among the high-occupancy and low-occupancy windows were kept separately. We then performed a regression of count(codon) in high occupancy windows∼count(codon) in low occupancy windows. The line y = x is plotted as a visual aid. (B) Standardized residuals from the analysis in (A) are plotted against the original x values in (A). No codons that are overrepresented in the Kozak sequence (i.e., have positive *Z*-scores) have standardized residuals greater than +1.96, implying they may be overused. The high-*Z* codon AAA comes close to the +1.96 mark, however we note that AAA encodes a positively charged amino acid, lysine, as do AAG and CGA, which also fall near the +1.96 mark and are not overused in the Kozak sequence. Horizontals are plotted at y = 1.96, +1.96. (C) Here the codon counts used in (A) were normalized by the usage of the corresponding amino acid to investigate fluctuations in synonymous codon choice given the amino acid in the protein. We then performed a regression of count(codon)/count(corresponding amino acid) in high occupancy windows∼count(codon)/count(corresponding amino acid) in low occupancy windows. The line y = x is plotted as a visual aid. (D) Standardized residuals from (C) are plotted against the original x values. We observe that those codons that are significantly overrepresented (i.e., over +1.96 standard deviations) in the high occupancy windows (given the amino acid content) are in fact underrepresented in the Kozak sequence (with a negative *Z*-score) compared to the genome at large. Even the AAA codon, above the +1.96 standard deviation mark in (B), is not overused when factoring in amino acid choice as shown here. We consider this confirmation of our inference that the AAA codon has a high residual in (B) on account of the amino acid it encodes, and not merely because of its similarity to Kozak sequence. For these reasons, although we cannot rule out a potential contribution to slowing, we consider that transcript similarity to the Kozak sequence cannot explain the bulk of ribosomal pausing in yeast.
(PDF)

Figure S8 Ribosomal slowing after positive charge clusters in the ribosomal footprint set taken from amino acid-starved yeast [29].
(PDF)

Figure S9 Changes in relative translation rates after rare codon clusters calculated from amino-acid-starved data [29]. Three rare codon clusters are plotted with outlier axes. (A) All genes with rare codon clusters. (B) Genes with rare codon clusters that have 0 or 1 positive charge coded for in the last 30 codon positions plotted. These plots represent the net effect of tAI on ribosomal density with the bulk of the effect of positive charge removed. (C) Genes with rare codon clusters that have two or more positive charges in the last 30 codon positions plotted.
(PDF)

Figure S10 Positive charges show an additive (linear) trend in slowing ribosomes in the amino-acid-starved dataset [29], but rare codons do not. The degree of slowing is a function of both the magnitude of ribosomal density and the length of transcript the slowing covers. Therefore to measure any trend in the ability of either positive charges or codon clusters to slowing, the area between the curves depicting the average relative change in ribosomal density (*r_pos_/r_prec30_*) and the *y* = 1 null in Figures S8 and S9, whether positive or negative, was summed between *x* = 0 (the beginning of the cluster) and the point where the plotted values intersect with *y* = 1 again (see Figure 1). A positive value for the area under the curve indicates ribosomal slowing, while a negative value reflects faster movement. (A) Regression of *area under curve*∼*size of cluster* +*0* gives a slope of 5.15 (*p* = 0.0122, *r* ^2^ = 0.7815). A linear model (not shown) that does not force the regression through the origin gives an insignificant intercept (*p* = 0.64). (B–D) Regression of *area under curve*∼*size of cluster + 0*, slope *p* = 0.56, 0.93, and 0.55, respectively.
(PDF)

Figure S11 Positive charges encoded by A/G- and C-rich codons both slow ribosomes. If positive charges indeed slow codons, we should detect slowing regardless of the codon encoding the charge. Since we are now considering specific subgroups among the positive charge clusters depending on the corresponding codon composition, sample size quickly becomes an issue. The 1-positive charge clusters give not only the best sample size, but also the fairest comparison since the composition of the “cluster” must be binary (either A/G- or C-rich) and not mixed. Our results show that positive charge slows ribosomes regardless of the nature of the codon encoding the charge. The C-rich codons (encoding Arg and His) may slow translation slightly less than the A-rich codons (Lys and Arg). This is to be expected, as histidine has a lesser tendency to be charged at physiological pH (see also Results).
(PDF)

Figure S12 Histidine-enriched clusters slow less than histidine-free clusters. As we note in the main text, histidine is less likely to be charged at physiological pH than lysine or arginine. Here we divide positive charge clusters according to whether or not they contain a minimal number of histidine residues versus no histidines at all and observe that greater slowing is observed after histidine-free clusters, in line with expectations if charge does slow ribosomes.
(PDF)

Figure S13 The only significantly overused amino acid in the high-ribosomal occupancy windows across genes (relative to the amino acid content in the paired low-occupancy windows in the same genes) is lysine, which is positively charged. In our main analysis we identified amino acids we expect to slow ribosomes (e.g., basic amino acids) and then examining the change in ribosomal occupancy upon their addition to the peptide chain. An alternative approach is to ask which amino acids are statistically overrepresented within the most slowly translated (i.e., most footprint-dense) regions within a gene. As different genes have their own expression levels, nucleotide contents, and functions, we would ideally like to control for these differences among genes when examining which amino acids are overused on the whole. For this reason, we re-employed a two-window analysis in which the highest ribosomal occupancy window and the lowest occupancy window (each of 10 codons) were identified in every gene for which we had ribosomal occupancy data. Tallies of all the amino acids used among the high-occupancy and low-occupancy windows (and including the preceding five codons before each window, as these amino acids may have just entered the tunnel when slowing occurs) were kept separately. We then performed a regression of *usage count(aa)*, *high occupancy windows*∼*usage count(aa)*, *low occupancy windows*: if all amino acids are used equally among the slowly translated and quickly translated windows, then the regression should give a slope of 1, with all data points falling precisely upon the regression line. We plotted the residuals of this regression against the low window count, such that amino acids that are significantly overused in the high-occupancy window will have standardized residuals of greater than +1.96. Only a positively charged amino acid (lysine) is significantly overused in the higher ribosomal occupancy window.
(PDF)

Figure S14 The effect of positive charge is not explained by covariance with codon usage or mRNA folding. In order to determine if global patterns of codon usage or mRNA secondary structure might in fact be contributing to patterns in ribosomal slowing we observe after clusters of positive charges, we also examined the relative changes in tAI and PARS values after the clusters. Within a given transcript, the relative increase or decrease in codon optimality at each position surrounding the charged cluster was calculated by dividing the measured ribosomal density at some codon position (tAI*_pos_*) (i.e., at some position before/after the charged residue is added) by the average tAI of the 30 codons preceding the first coded-for charge in the cluster within that transcript (tAI*_prec30_*). The mean relative change in tAI after a cluster positive charges was then calculated by aligning all transcripts with a given cluster size by the first charge in each cluster and calculating the average ratio (tAI*_pos_*/tAI*_prec30_*) in each codon site surrounding the cluster. We similarly calculated the relative increase or decrease in propensity for double-stranded structure, as quantified by PARS values, at each position surrounding the charged cluster. As PARS values as originally published [38] are logged ratios, we first took the antilog of all PARS values (making all of them positive) in order to be able to calculate relative increases or decreases in the values along transcripts by dividing the antilogged PARS value at some codon position surrounding the encoded charge cluster (PARS*_pos_*) by the average PARS of the 30 codons (all previously antilogged) preceding the first coded-for charge in the cluster within that transcript (PARS*_prec30_*). This method is conservative, as taking the antilog will result in PARS values indicating single-strandedness being sandwiched between 0 and 1, but with PARS values indicating double-strandedness spread above 1. Hence increases in double-stranded propensity will be exaggerated. The average relative change in either tAI or PARS (mean tAI*_pos_*/tAI*_prec30_* or PARS*_pos_*/PARS*_prec30_*) at a given position after a cluster was then calculated by aligning all identified regions of a given cluster size according to the first charge present in each cluster and calculating the average ratio in positions increasingly distant from the first positive charge of the aligned clusters. Positive charges in a cluster may be coded for anywhere between the two downturned triangles. An average *r_pos_/r_prec30_* above 1 indicates a relative local increase in ribosomal density in that position across transcripts (as in Figure 1). (A) An average tAI*_pos_*/tAI*_prec30_* below 1 indicates the codons in that position across transcripts tend to decrease in optimality on average relative to the average tAI of the preceding 30 codons across transcripts, while a ratio above 1 signifies an increase in optimality. We find that differential codon use in the vicinity of positive charges cannot explain the charge slowing effect. We observe no correlation between relative changes in ribosomal density and tAI after the first charge in the cluster (0≥*x*≤30 in this figure, panel A; Spearman P, left to right: 0.93, 0.73, 0.22, 0.17, and 0.65). For a more relaxed test, we then compared, for each plot in Figure 5, the relative changes in codon optimality (tAI*_pos_*/tAI*_prec30_*) seen after the start of each cluster at *x* = 0 until the point where relative change in ribosomal density (*r_pos_/r_prec30_*) drops back to previous levels (*y* = 1) to the tAI*_pos_*/tAI*_prec30_* values seen in all other surrounding plotted sites (i.e., those sites lacking charge-induced pausing). If anything, relatively more optimal (tAI*_pos_*/tAI*_prec30_*>1) codons are coded for during periods of elevated ribosomal occupancy for clusters comprising six or more encoded cations, while no difference in optimality is detected in codon usage during elevated ribosomal occupancy compared to surrounding codon usage for other-sized charge clusters (Mann-Whitney U test *p* values, left to right in this figure, panel A: 0.96, 0.20, 0.07, 0.07, and 0.003). Hence we conclude that changes in codon bias are not responsible for the slowing patterns associated with positively charged residues (Figure 5), as expected if rare codons do not slow ribosomes (Figure 3A,B). (B) An average relative change in (here antilogged, see Methods) PARS values (i.e., PARS*_pos_*/PARS*_prec30_*) plotted above 1 indicates a greater likelihood of double-stranded structure in that position on average relative to preceding sequence, while a ratio less than 1 indicates a decrease in propensity for double-strandedness relative to the preceding 30 codons. We find that the slowing effect of positive charge cannot be explained by mRNA folding in the vicinity of positive charges. There is no correlation between the relative change in PARS values (PARS*_pos_*/PARS*_prec30_*) after the first charge in the cluster (this Figure, panel B, 0≥*x*≤30) and relative changes in ribosomal density (Spearman P, left to right: 0.44, 0.68, 0.97, 0.99, and 0.15), which we may have expected to observe if RNA structure has a local effect on ribosomal slowing. Likewise, under such a local-slowing hypothesis, we should expect to see a significant difference in the average PARS ratios seen among the sequence between *x* = 0 and the point at which elevated ribosomal density curve (*r_pos_/r_prec30_*) drops back to *y* = 1 versus PARS ratios in surrounding plotted sites. Such a difference, however, is seen only in the two-charge plot (this figure, panel B; Mann-Whitney U test *p* values, left to right: 0.17, 0.0006, 0.24, 0.08, and 0.60). If we instead assume that downstream structure has a pausing effect observable more upstream, a more appropriate test is to compare the PARS ratios from −30≥*x*<0 to those from 0≥*x*≤30. In this case, we observe no significant difference in relative propensity for double-strandedness before or after positive charges apart from in the case of a single positive charge alone [this figure, panel B; Mann-Whitney U test, left to right: 0.004, 0.07, 0.12, 0.08 (with the mean PARS*_pos_*/PARS*_prec30_* decreasing on average after the start of the cluster), and 0.60]. We note that this version of the test is exceedingly conservative as PARS values had to be antilogged before informative ratios could be calculated. This means that previously negative values (indicating single-strandedness) will now be sandwiched in between 0 and 1, while formerly positive values (indicating double-strandedness) now span a range of values above 1. Hence normalizing the PARS score at a given position by the average PARS value of the preceding 30 codons will exaggerate not only the importance of structured versus free-form RNA, but will also exaggerate small differences in the magnitude of PARS values already denoting double-strandedness. (C) An alternative calculation showing that RNA structure does not account for the pausing observed near positive charges. Note this figure does not show the change in PARS values relative to the preceding sequence (as in B), but the average magnitude of the PARS value in that position across aligned transcripts. An average of PARS values plotted above 0 indicates a greater likelihood of double-stranded structure in that position on average, while a mean value of less than 1 indicates a propensity for single-strandedness. We find no correlation between the average PARS values after the first charge in the cluster (0≥x≤30) and relative changes in ribosomal density (this figure, panel C; Spearman P, left to right: 0.77, 0.95, 0.87, 0.34, and 0.09), as we might have observed if RNA structure has a local effect on ribosomal slowing. Likewise, if structure causes local slowing, we should see a significant difference in the average PARS values between *x* = 0 and the point at which elevated ribosomal density curve (*r_pos_/r_prec30_*) drops back to *y* = 1 versus PARS values in surrounding plotted sites. We do not, however, detect such a difference (this figure, panel C; Mann-Whitney U test *p* values, left to right: 0.66, 0.17, 0.30, 0.27, and 0.90). Examining whether downstream structure has a pausing effect observable further upstream, we then compare the PARS ratios from −30≥*x*<0 to those from 0≥*x*≤30. In this case, we observe no significant difference in relative propensity for double-strandedness before or after positive charges (this figure, panel C; Mann-Whitney U test, left to right: 0.98, 0.98, 0.97, 0.27, and 0.90).
(PDF)

Figure S15 Genes with either high or low footprint coverage both produce consistent slowing patterns after positive charge clusters. To ensure that noise in the location of footprints among genes with fewer overall footprints is not an issue for analysis, we redrew our *r_pos_/r_prec30_* plots surrounding positive charge clusters using both the bottom half and top half of all genes according to their footprint saturation. Note that in this analysis we do not normalize the footprint counts per codon per gene by mRNA levels. This is because we are not interested in footprint coverage per transcript (as we might be if considering rates or mechanistic issues), but in the statistical power that the total footprint coverage per gene gives us, regardless of the number of transcripts that the footprints were captured from. Areas under the curve were measured as in the main text (see Figure 1). In each case we find similar results to those presented in the main analysis (Figure 5), namely that positive charges additively slow ribosomes. (A) Bottom half of genes: Regression of *area under curve*∼*cluster size*, slope = 4.8, *r^2^* = 0.79, *p* = 0.027. (B) Top half of genes: Regression of *area under curve*∼*cluster size*, slope = 0.96, *r^2^* = 0.74, *p* = 0.039.
(PDF)

Figure S16 Figure 5 redone on the nonredundant footprint set. We wanted to confirm that the exclusion of footprints that map to two or more potential locations in the genome was not systematically biasing our estimates of ribosomal density. For this reason we replotted the average relative change in ribosomal density within a gene upon translation of encoded positive charge clusters using our nonredundant footprint set (see the end of the Methods section), in effect only considering those locations in the genome to which footprints uniquely map. Considering solely these regions in the transcriptome to which footprints can only ever be mapped unambiguously still shows positive charges additively slow translation.
(PDF)

Figure S17 Figure 2 redone on the nonredundant footprint set. We wanted to confirm that the exclusion of footprints that map to two or more potential locations in the genome was not systematically biasing our estimates of ribosomal density. For this reason we replotted the average relative change in ribosomal density within a gene upon translation of rare codon clusters using our nonredundant footprint set (see the end of the Methods section), in effect only considering those locations in the genome to which footprints uniquely map. Considering solely these regions in the transcriptome to which footprints can only ever be mapped unambiguously still shows rare codons do not slow translation. (A) All genes with rare codon clusters. (B) Genes with rare codon clusters that have 0 or 1 positive charge coded for in the last 30 codon positions plotted. These plots represent the net effect of tAI on ribosomal density with the bulk of the effect of positive charge removed. (C) Genes with rare codon clusters that have two or more positive charges in the last 30 codon positions plotted.
(PDF)

Note S1 Codon usage and translation rates: how can codon usage not predict ribosome occupancy but be commonly assumed to be associated with faster translation?
(PDF)

Note S2 Only positive charge is capable of explaining the region of strongest translational pausing within transcripts.
(PDF)

Note S3 Trend of slowing increasing with charge is not random.
(PDF)

Table S1 Table 1 of the main text redone using rare codons that are defined to occur with genomic infrequency shows rare codons do not slow ribosomes. In the main text, we investigate whether nonoptimal codons—that is, those with low tAI scores—might slow codons and find that they do not. To ensure that our finding that these “rare” codons do not slow ribosomes does not simply hinge on our definition of “rare,” we have repeated the analysis using an alternative definition. Here, we define “rare” codons according to their actual frequency in the genome as measured from our set of filtered genes. This rare set, of equal size to the rare tAI set, comprises the following codons: CGG, CGC, CGA, TGC, CCG, CTC, GGG, GCG, CGT, CCC, CAC, TGT, ACG, TCG, and AGG. Quantiles of the difference in average ribosomal density between the two windows identified within a transcript are shown, with q1 representing the smallest differences and q4 the largest. A score of 1 indicates the putative retarding feature is more present within the more occluded intra-transcript window; −1, less present; 0, present in both windows in equal amounts. Rare (infrequent) codons and codon pairs tend to be found more in the less dense (faster translated) window. Similarly, the presence of rare pairs and rare codons decreases in the slowly translated windows as the difference in degree of ribosomal slowing grows.
(PDF)

Table S2 Genes with identified rare codon clusters are not disproportionately sampled from lowly expressed genes. Could it be that large changes in ribosomal occupancy are not observed after rare clusters (Figure 2A and Figure 3A) because the clusters we identify are more likely to come from lowly expressed genes—that is, genes that do not have high translation levels and for which it may be less likely that ribosomal footprints will be sampled? We used the average footprint count of a gene (total number of footprints within the coding sequence divided by gene length) as a proxy for protein expression levels. If anything, there are more genes with nonoptimal codon clusters from genes that have more footprint reads (χ^2^, *p*<2.2e-16) so we do not consider this an issue.
(PDF)

Table S3 Sequence similarity to the yeast Kozak sequence cannot explain the greatest slowing within transcripts. Given that transcript similarity to the Shine-Dalgarno sequence has been shown to slow ribosomes in bacteria due to interactions of the sequence with components of the ribosomal RNA [17], we wondered whether translation speed in yeast might not be modulated by codon usage per se but by the ability of ribosomes to bind to transcript sequence that mirrors the eukaryotic Kozak sequence. Specifically, we wanted to determine whether codons that are in high-ribosomal occupancy windows within a gene might be more likely to correspond to the Kozak sequence (as compared to codons in low-occupancy windows within the same genes) and hence bind ribosomes, slowing translation. We first determined which codons were enriched in the Kozak sequence relative to the codon frequencies seen throughout the yeast genome at large using a simple randomization. Nucleotide frequencies at each position of the Kozak sequence in yeast were taken from Cavener and Ray 1991 [57]. To determine the frequencies of all the possible “codons” among the Kozak sequence space, we randomly created 20,000 possible Kozak sequences from the delineated nucleotide frequencies at each site in the consensus sequence. We then counted all possible triplet “codons” within each sequence, regardless of reading frame (since we assume that as the ribosome traverses RNA, it may bind the Kozak sequence regardless of the surrounding reading frame). The counts of all possible RNA triplets that we observe within our simulated sequences are the observed “codons” within the Kozak sequence. In order to determine whether or not certain codons are over- or underused in the Kozak sequence, we compare them to the counts of codons observed (again in any reading frame) across 20,000 randomized sequences derived from the basal codon frequencies in the *S. cerevisiae* genome and of the same length as the Kozak sequence. We calculate *Z*, a measure of the over- or underusage of a particular codon within the Kozak sequence (as compared to the rest of the genome) as *Z*_codon_ = [Observed codon count (in Kozak sequence) – Expected count (from genome frequencies)]/Expected SD of codon. We can then perform a test similar to the one in Methods, “The Relative Contributions of Charge, Folding, and Codon Usage to Extremes of Slowing Within Transcripts,” but where we consider possible slowing codons to be those with a positive *Z* (GAT GAC AAC TGC CAA GGC GTA GTC TAT ACA TGG ATA CAT AAA TGT AAT ATG). A score of 1 indicates there are more codons with positive *Z* within the more occluded intra-transcript window; −1, less present; 0, present in both windows in equal amounts. (A) Similarity to Kozak sequence cannot explain slowing in several quantiles (binomial tests), nor can it explain increased slowing (χ^2^ tests). (B) Even when the number of positive charges is the same between the two windows, we do not detect a significant contribution of similarity to Kozak sequence to slowing. (C) Controlling for amino acid usage in two different ways, we detect no contribution of similarity to Kozak sequence to slowing; in fact, as the degree of slowing increases, the ability of Kozak similarity to explain slowing decreases (χ^2^ tests). Method 1 (in bold): a gene is scored “1” if the slow window contains more codons with positive *Z*, “−1” if it contains fewer. Method 2 (in italics): the magnitude of all the positive *Z* values is averaged in each window, and the gene is scored “1” if the slower window has a higher average *Z*, “−1” if its average *Z* is lower.
(PDF)

Table S4 Table 1 tAI score tests controlled for amino acid content. Could differences in amino acid usage between the two windows be biasing our result that neither codon usage nor rare pairs slow ribosomes (Table 1)? It could be that certain amino acids only have relatively high or low tAIs, and a preponderance of such amino acids in one window over the other could cause an apparent preference for (non-)optimal codons, which is in fact a preference for a certain amino acid. For this reason we tested whether differences in amino acid usage between the high and low ribosomal occupancy windows within a transcript systematically alter the tAI scores (and hence the resulting interpretation of the contribution of codon usage to ribosomal density) in our window comparison analysis. To do this, we identified the same high and low ribosomal occupancy windows within a transcript as above. This time, however, we considered only amino acids that are coded for at least once within each window. Within each intra-transcript window, we identified all codons that code for amino acid *x* and quantified the contribution of tAI to ribosomal occupancy using two approaches: (Method 1) The average tAI of all the codons coding for amino acid (aa) *x* was calculated for each window, and that amino acid was assigned an aa-tAIscore of 1, 0, or −1, depending on whether the tAI in the higher ribosomal occupancy window was lower (and hence capable of explaining the increased ribosomal density), the same, or higher than that in the other window, respectively. All of the aa-tAI scores for a given gene were counted independently—in other words, for a given gene, it was possible to calculate more than one aa-tAI score, and all these aa-tAI scores contributed to the final matrix. (Method 2) The average tAI of all the codons coding for amino acid *x* in each window was calculated, similarly to Method 1, but a tAI score is not yet assigned. Instead, the average tAI is first determined for each amino acid present in both windows, and then average tAIs (each the average for a particular amino acid) are themselves averaged to come up with a single aa-tAI for each window. Then, a single tAI score is assigned to that gene by comparing the average aa-tAIs in each window similarly to above. Bold, Method 1; italic, Method 2. Original Δ*r* quantiles means the same quantile boundaries used in the main analysis were used, whereas recalculated Δ*r* quantiles are drawn from only those genes considered in this amino-acid-adjusted analysis. The *p* value for χ^2^ tests with fewer than five observations in any square was calculated by resampling the observations without replacement and noting how many times (*r*) the χ^2^ value of the resampled set was greater than or equal to the observed. The *p* was then calculated as (*r*+1)/(*n*+1), where *n* is the number of iterations performed (1,000). (A) Upon controlling for differential amino acid content in the two windows as detailed above, the result that tAI cannot explain patterns of slowing is still robust. Additionally we no longer detect a decrease in the ability of tAI to explain pausing in the upper quantiles as observed in Table 1A. (B) and (C) show the effect of tAI (adjusted for amino acid use) in only those pairs of intra-transcript windows that have the same number of positive charges between them.
(PDF)

Table S5 Table 1 done again on the amino-acid-starved footprint set [29]. Only positive charge is systematically capable of explaining ribosomal slowing, including the severest slowing. Quantiles of the difference in average ribosomal density between the two windows identified within a transcript are shown, with q1 representing the smallest differences and q4 the largest. A score of 1 indicates the putative retarding feature is more present within the more occluded intra-transcript window; −1, less present; 0, present in both windows in equal amounts. A low codon optimality, if anything, tends to pair more with the less dense (faster translated) window. Similarly, not only do rare pairs and rare 6-mers tend to be found more often in the faster-translated window, but their presence decreases as the difference in degree of ribosomal slowing grows. Additionally, a greater likelihood of transcript secondary structure at or just before the identified window is associated not with the more occluded windows, but with the less dense (faster translated) ones, and the presence of secondary structure in fact decreases as the difference in ribosomal slowing between the windows increases. Positive charge, however, is consistently associated with the higher density (more slowly translated) window.
(PDF)

Table S6 Positive charge best explains the slowest translated regions within transcripts compared to other physiochemical properties of amino acids. While we find that positive charges slow ribosomes, we wanted to control for the effects of other physiochemical properties of amino acids, specifically hydropathy (Phe, Val, Leu, Ile, Met), polarity (Asn, Gln, Ser, Thr, Cys, Tyr), and negative charge (Asp, Glu). These groups of amino acids, however, do not lend themselves to the *r_pos_/r_prec30_* analysis we carry out in the main text (see Figures 1–5) in the same way that positive charge does. The *r_pos_/r_prec30_* plotting analysis is suited to positive charges because they cluster in a way that gives us reasonable sample sizes given our constraints—that is, the number of positive charges we require in the cluster and the additional requirement that there be no surrounding positive charges outside of the cluster. In the case of the other amino acid groups, there are either too many constituent members of the group and which are used too frequently (e.g., hydropathy) to define isolated “clusters” for investigation, or the amino acids are used too rarely as clusters away from positive charges, and are of insufficient cluster sizes to establish any slowing trends (e.g., negative charges). We therefore compared the effects of these other physiochemical properties of amino acids by comparing the amino acids encoded by the highest ribosomally occupied versus lowest occupied windows within genes. The analysis was carried out similarly to the way Table 1 was created in the main text, only this time counting different amino acids depending on the physiochemical property being investigated. We find that, on the whole, only positive charge can robustly explain the slowing patterns we observe. Quantiles of the difference in average ribosomal density between the two windows identified within a transcript are shown, with q1 representing the smallest differences and q4 the largest. A score of 1 indicates the putative retarding feature is more present within the more occluded intra-transcript window; −1, less present; 0, present in both windows in equal amounts. (A) Hydrophobic residues (Phe, Val, Leu, Ile, Met) cannot explain increased slowing as the difference in translation speed between the two windows increases (χ^2^ *p* = 0.98). Additionally the proportion of genes that pass the hydrophobicity test compared to failing it is only significant in the fourth quantile (q4) (binomial *p* = 0.023). (B) Polar residues (Asn, Gln, Ser, Thr, Cys, Tyr) cannot explain increased slowing as the difference in translation speed between the two windows increases (χ^2^ *p* = 0.21). Additionally the proportion of genes that pass the polarity test compared to failing it is only significant in the fourth quantile (q4) (binomial *p* = 3.7e-08). (C) Negative charges (Asp, Glu) cannot explain increased slowing as the difference in translation speed between the two windows increases (χ^2^ *p* = 0.14). Additionally the number of genes that pass or fail the negative charge score test in the third quantile (q3) is not significantly different (binomial *p* = 0.83). (D) Positive charge score, from Table 1, is shown for purposes of comparison.
(PDF)

Table S7 Positive charge explains slowing better than amino acid hydrophobicity. Quantiles of the difference in average ribosomal density between the two windows identified within a transcript are shown, with q1 representing the smallest differences and q4 the largest. A score of 1 indicates the putative retarding feature is more present within the more occluded intra-transcript window; −1, less present; 0, present in both windows in equal amounts. (A–C) In those genes that fail the positive charge test (charge score = 0 or −1), we find that hydrophobicity cannot explain the increased slowing in these windows either (this table, χ^2^ tests). For this reason we consider that while amino acids with hydrophobic side chains may be used more often in the vicinity of positive charge (this table, binomial tests), perhaps for certain structural motifs or because of the types of genes under consideration, they cannot be responsible for the major slowing effect. (D–F) Positive charge can explain the slowing in genes where hydrophobicity cannot.
(PDF)

Table S8 Positive charge explains slowing better than amino acid polarity. Quantiles of the difference in average ribosomal density between the two windows identified within a transcript are shown, with q1 representing the smallest differences and q4 the largest. A score of 1 indicates the putative retarding feature is more present within the more occluded intra-transcript window; −1, less present; 0, present in both windows in equal amounts. (A–C) In those genes that fail the positive charge test (charge score = 0 or −1), we find that polarity cannot explain the increased slowing in these windows either (this table, χ^2^ tests). For this reason we consider that while amino acids with polar side chains may be used more often in the vicinity of positive charge (this table, binomial tests), perhaps for certain structural motifs or because of the types of genes under consideration, they cannot be responsible for the major slowing effect. (D–F) Positive charge can explain the slowing in some genes where polarity cannot.
(PDF)

Table S9 Positive charge explains slowing better than negative charge. Quantiles of the difference in average ribosomal density between the two windows identified within a transcript are shown, with q1 representing the smallest differences and q4 the largest. A score of 1 indicates the putative retarding feature is more present within the more occluded intra-transcript window; −1, less present; 0, present in both windows in equal amounts. (A–C) In those genes that fail the positive charge test (charge score = 0 or −1), we find that negatively charged amino acids cannot explain the increased slowing in these windows either (this table, χ^2^ tests). For this reason we consider that while amino acids with negatively charged side chains may be used more often in the vicinity of positive charge (this table, binomial tests), perhaps for certain structural motifs or because of the types of genes under consideration, they cannot be responsible for the major slowing effect. (D–F) Positive charge can explain the slowing in genes where negative charge cannot.
(PDF)

Table S10 The relationship of charge score to tAI score. Quantiles of the difference in average ribosomal occlusion between the two windows identified within a transcript are shown, with q1 representing the smallest differences and q4 the largest. A score of 1 indicates the putative retarding feature is more present within the more occluded intra-transcript window; −1, less present; 0, present in both windows in equal amounts. (A) The ability of charge to explain slowing (charge score of 1) cannot be explained by concomitant use of suboptimal codons. A charge score of 1 more commonly pairs with a tAI score, which cannot explain slowing (tAI score of −1), and increasingly so as the difference in ribosomal speeds between the two windows grows. (B) These tAI scores are drawn from transcripts for which both intra-transcript windows have the same number of charges (charge score = 0) and hence such comparisons should be controlled for the effect of positive charge on ribosomal speed. Different tAI scores are equally distributed among quantiles, indicating the inability of tAI to predict either ribosomal slowing or the degree of ribosomal slowing even in the absence of an effect of charge on ribosomal speed. (C) tAI does not systematically account for slowing in windows for which increased charge pairs with the faster window.
(PDF)

Table S11 The relationship of rare pair score to charge score. Quantiles of the difference in average ribosomal occlusion between the two windows identified within a transcript are shown, with q1 representing the smallest differences and q4 the largest. A score of 1 indicates the putative retarding feature is more present within the more occluded intra-transcript window; −1, less present; 0, present in both windows in equal amounts. (A) The ability of charge to explain slowing (charge score of 1) cannot be explained by concomitant use of rare pairs. A charge score of 1, if anything, tends to pair with a rare pair score that cannot explain slowing (rare pair score of −1). (B) These rare pair scores are drawn from transcripts for which both intra-transcript windows have the same number of charges (charge score = 0) and hence such comparisons should be controlled for the effect of positive charge on ribosomal speed. Different rare pair scores are equally distributed among quantiles, indicating the inability of rare pairs to predict ribosomal slowing. Additionally, as the difference in the degree of ribosomal slowing increases (i.e., moving from q1 to q4), the number of rare pairs found in the higher occupancy window decreases (χ^2^ test), demonstrating rare pairs cannot predict the magnitude of slowing even in the absence of an effect of charge on ribosomal speed. (C) Rare pairs do not systematically account for slowing in windows for which increased charge pairs with the faster window.
(PDF)

Table S12 The relationship of PARS score (double strandedness) to charge score. Quantiles of the difference in average ribosomal occlusion between the two windows identified within a transcript are shown, with q1 representing the smallest differences and q4 the largest. A score of 1 indicates the putative retarding feature is more present within the more occluded intra-transcript window; −1, less present; 0, present in both windows in equal amounts.
(PDF)

Table S13 Table 1 done again, allowing the lower occupancy window to have a ribosomal occupancy of 0.
(PDF)

Table S14 Table 1 done again on the non-redundant footprint location set.
(PDF)

## Abbreviations

PARS: parallel analysis of RNA structure
tAI: tRNA adaptation index

## References

[1] Randall LL, Josefsson LG, Hardy SJ (1980) Novel intermediates in the synthesis of maltose-binding protein in Escherichia coli. Eur J Biochem 107: 375–379. 6995119 10.1111/j.1432-1033.1980.tb06039.x

[2] Siller E, DeZwaan DC, Anderson JF, Freeman BC, Barral JM (2010) Slowing bacterial translation speed enhances eukaryotic protein folding efficiency. J Mol Biol 396: 1310–1318. 20043920 10.1016/j.jmb.2009.12.042

[3] Doma MK, Parker R (2006) Endonucleolytic cleavage of eukaryotic mRNAs with stalls in translation elongation. Nature 440: 561–564. 16554824 10.1038/nature04530 PMC1839849

[4] Yanofsky C (1981) Attenuation in the control of expression of bacterial operons. Nature 289: 751–758. 7007895 10.1038/289751a0

[5] Chartrand P, Meng XH, Huttelmaier S, Donato D, Singer RH (2002) Asymmetric sorting of ash1p in yeast results from inhibition of translation by localization elements in the mRNA. Mol Cell 10: 1319–1330. 12504008 10.1016/s1097-2765(02)00694-9

[6] Mariappan M, Li X, Stefanovic S, Sharma A, Mateja A, et al (2010) A ribosome-associating factor chaperones tail-anchored membrane proteins. Nature 466: 1120–1124. 20676083 10.1038/nature09296 PMC2928861

[7] Ikemura T (1981) Correlation between the abundance of Escherichia coli transfer RNAs and the occurrence of the respective codons in its protein genes: a proposal for a synonymous codon choice that is optimal for the E. coli translational system. J Mol Biol 151: 389–409. 6175758 10.1016/0022-2836(81)90003-6

[8] Kimchi-Sarfaty C, Oh JM, Kim IW, Sauna ZE, Calcagno AM, et al (2007) A “silent” polymorphism in the MDR1 gene changes substrate specificity. Science 315: 525–528. 17185560 10.1126/science.1135308

[9] Anderson WF (1969) The effect of tRNA concentration on the rate of protein synthesis. Proc Natl Acad Sci U S A 62: 566–573. 4894331 10.1073/pnas.62.2.566 PMC277843

[10] Gouy M, Gautier C (1982) Codon usage in bacteria—correlation with gene expressivity. Nucleic Acids Res 10: 7055–7074. 6760125 10.1093/nar/10.22.7055 PMC326988

[11] Thanaraj TA, Argos P (1996) Ribosome-mediated translational pause and protein domain organization. Protein Sci 5: 1594–1612. 8844849 10.1002/pro.5560050814 PMC2143486

[12] Cortazzo P, Cervenansky C, Marin M, Reiss C, Ehrlich R, et al (2002) Silent mutations affect in vivo protein folding in Escherichia coli. Biochem Biophys Res Commun 293: 537–541. 12054634 10.1016/S0006-291X(02)00226-7

[13] Grantham R, Gautier C, Gouy M, Jacobzone M, Mercier R (1981) Codon catalog usage is a genome strategy modulated for gene expressivity. Nucleic Acids Res 9: r43–r74. 7208352 10.1093/nar/9.1.213-b PMC326682

[14] Bennetzen JL, Hall BD (1982) Codon selection in yeast. J Biol Chem 257: 3026–3031. 7037777

[15] Ingolia NT, Lareau LF, Weissman JS (2011) Ribosome profiling of mouse embryonic stem cells reveals the complexity and dynamics of mammalian proteomes. Cell 147: 789–802. 22056041 10.1016/j.cell.2011.10.002 PMC3225288

[16] Qian W, Yang J-R, Pearson NM, Maclean C, Zhang J (2012) Balanced codon usage optimizes eukaryotic translational efficiency. PLoS Genet 8: e1002603 doi:10.1371/journal.pgen.1002603. 22479199 10.1371/journal.pgen.1002603 PMC3315465

[17] Li GW, Oh E, Weissman JS (2012) The anti-Shine-Dalgarno sequence drives translational pausing and codon choice in bacteria. Nature 484: 538–541. 22456704 10.1038/nature10965 PMC3338875

[18] Akashi H (1994) Synonymous codon usage in *Drosophila melanogaster*: natural selection and translational accuracy. Genetics 136: 927–935. 8005445 10.1093/genetics/136.3.927 PMC1205897

[19] Stoletzki N, Eyre-Walker A (2007) Synonymous codon usage in Escherichia coli: selection for translational accuracy. Mol Biol Evol 24: 374–381. 17101719 10.1093/molbev/msl166

[20] Precup J, Parker J (1987) Missense misreading of asparagine codons as a function of codon identity and context. J Biol Chem 262: 11351–11355. 3112158

[21] Warnecke T, Hurst LD (2010) GroEL dependency affects codon usage-support for a critical role of misfolding in gene evolution. Molecular Systems Biology 6: 340. 20087338 10.1038/msb.2009.94 PMC2824523

[22] Wen JD, Lancaster L, Hodges C, Zeri AC, Yoshimura SH, et al (2008) Following translation by single ribosomes one codon at a time. Nature 452: 598–603. 18327250 10.1038/nature06716 PMC2556548

[23] Somogyi P, Jenner AJ, Brierley I, Inglis SC (1993) Ribosomal pausing during translation of an RNA pseudoknot. Mol Cell Biol 13: 6931–6940. 8413285 10.1128/mcb.13.11.6931-6940.1993 PMC364755

[24] Kozak M (1986) Influences of mRNA secondary structure on initiation by eukaryotic ribosomes. Proc Natl Acad Sci U S A 83: 2850–2854. 3458245 10.1073/pnas.83.9.2850 PMC323404

[25] Lu J, Kobertz WR, Deutsch C (2007) Mapping the electrostatic potential within the ribosomal exit tunnel. J Mol Biol 371: 1378–1391. 17631312 10.1016/j.jmb.2007.06.038

[26] Lu J, Deutsch C (2008) Electrostatics in the ribosomal tunnel modulate chain elongation rates. J Mol Biol 384: 73–86. 18822297 10.1016/j.jmb.2008.08.089 PMC2655213

[27] Tuller T, Carmi A, Vestsigian K, Navon S, Dorfan Y, et al (2010) An evolutionarily conserved mechanism for controlling the efficiency of protein translation. Cell 141: 344–354. 20403328 10.1016/j.cell.2010.03.031

[28] Tuller T, Veksler-Lublinsky I, Gazit N, Kupiec M, Ruppin E, et al (2011) Composite effects of gene determinants on the translation speed and density of ribosomes. Genome Biol 12: R110. 22050731 10.1186/gb-2011-12-11-r110 PMC3334596

[29] Ingolia NT, Ghaemmaghami S, Newman JR, Weissman JS (2009) Genome-wide analysis in vivo of translation with nucleotide resolution using ribosome profiling. Science 324: 218–223. 19213877 10.1126/science.1168978 PMC2746483

[30] Tuller T, Waldman YY, Kupiec M, Ruppin E (2010) Translation efficiency is determined by both codon bias and folding energy. Proc Natl Acad Sci U S A 107: 3645–3650. 20133581 10.1073/pnas.0909910107 PMC2840511

[31] Bulmer M (1991) The selection-mutation-drift theory of synonymous codon usage. Genetics 129: 897–907. 1752426 10.1093/genetics/129.3.897 PMC1204756

[32] dos Reis M, Savva R, Wernisch L (2004) Solving the riddle of codon usage preferences: a test for translational selection. Nucleic Acids Research 32: 5036–5044. 15448185 10.1093/nar/gkh834 PMC521650

[33] Kane JF (1995) Effects of rare codon clusters on high-level expression of heterologous proteins in Escherichia coli. Curr Opin Biotechnol 6: 494–500. 7579660 10.1016/0958-1669(95)80082-4

[34] Varenne S, Baty D, Verheij H, Shire D, Lazdunski C (1989) The maximum rate of gene expression is dependent on the downstream context of unfavourable codons. Biochimie 71: 1221–1229. 2517483 10.1016/0300-9084(89)90027-8

[35] Frenkel-Morgenstern M, Danon T, Christian T, Igarashi T, Cohen L, et al (2012) Genes adopt non-optimal codon usage to generate cell cycle-dependent oscillations in protein levels. Mol Syst Biol 8: 572. 22373820 10.1038/msb.2012.3 PMC3293633

[36] Brackley CA, Romano MC, Thiel M (2011) The dynamics of supply and demand in mRNA translation. PLoS Comput Biol 7: e1002203 doi:10.1371/journal.pcbi.1002203. 22022250 10.1371/journal.pcbi.1002203 PMC3192816

[37] Elf J, Nilsson D, Tenson T, Ehrenberg M (2003) Selective charging of tRNA isoacceptors explains patterns of codon usage. Science 300: 1718–1722. 12805541 10.1126/science.1083811

[38] Kertesz M, Wan Y, Mazor E, Rinn JL, Nutter RC, et al (2010) Genome-wide measurement of RNA secondary structure in yeast. Nature 467: 103–107. 20811459 10.1038/nature09322 PMC3847670

[39] Ito-Harashima S, Kuroha K, Tatematsu T, Inada T (2007) Translation of the poly(A) tail plays crucial roles in nonstop mRNA surveillance via translation repression and protein destabilization by proteasome in yeast. Genes Dev 21: 519–524. 17344413 10.1101/gad.1490207 PMC1820893

[40] Ikemura T (1982) Correlation between the abundance of yeast transfer RNAs and the occurrence of the respective codons in protein genes. Differences in synonymous codon choice patterns of yeast and Escherichia coli with reference to the abundance of isoaccepting transfer RNAs. J Mol Biol 158: 573–597. 6750137 10.1016/0022-2836(82)90250-9

[41] Curran JF, Yarus M (1989) Rates of aminoacyl-tRNA selection at 29 sense codons in vivo. J Mol Biol 209: 65–77. 2478714 10.1016/0022-2836(89)90170-8

[42] Nakatogawa H, Ito K (2002) The ribosomal exit tunnel functions as a discriminating gate. Cell 108: 629–636. 11893334 10.1016/s0092-8674(02)00649-9

[43] Bhushan S, Meyer H, Starosta AL, Becker T, Mielke T, et al (2010) Structural basis for translational stalling by human cytomegalovirus and fungal arginine attenuator peptide. Mol Cell 40: 138–146. 20932481 10.1016/j.molcel.2010.09.009

[44] Fang P, Spevak CC, Wu C, Sachs MS (2004) A nascent polypeptide domain that can regulate translation elongation. Proc Natl Acad Sci U S A 101: 4059–4064. 15020769 10.1073/pnas.0400554101 PMC384695

[45] Brown CE, Sachs AB (1998) Poly(A) tail length control in Saccharomyces cerevisiae occurs by message-specific deadenylation. Mol Cell Biol 18: 6548–6559. 9774670 10.1128/mcb.18.11.6548 PMC109240

[46] Meaux S, Van Hoof A (2006) Yeast transcripts cleaved by an internal ribozyme provide new insight into the role of the cap and poly(A) tail in translation and mRNA decay. RNA 12: 1323–1337. 16714281 10.1261/rna.46306 PMC1484436

[47] Inada T, Aiba H (2005) Translation of aberrant mRNAs lacking a termination codon or with a shortened 3′-UTR is repressed after initiation in yeast. EMBO J 24: 1584–1595. 15933721 10.1038/sj.emboj.7600636 PMC1142571

[48] Akimitsu N, Tanaka J, Pelletier J (2007) Translation of nonSTOP mRNA is repressed post-initiation in mammalian cells. EMBO J 26: 2327–2338. 17446866 10.1038/sj.emboj.7601679 PMC1864977

[49] Dimitrova LN, Kuroha K, Tatematsu T, Inada T (2009) Nascent peptide-dependent translation arrest leads to Not4p-mediated protein degradation by the proteasome. J Biol Chem 284: 10343–10352. 19204001 10.1074/jbc.M808840200 PMC2667721

[50] Gillet R, Felden B (2001) Emerging views on tmRNA-mediated protein tagging and ribosome rescue. Mol Microbiol 42: 879–885. 11737633 10.1046/j.1365-2958.2001.02701.x

[51] Bengtson MH, Joazeiro CA (2010) Role of a ribosome-associated E3 ubiquitin ligase in protein quality control. Nature 467: 470–473. 20835226 10.1038/nature09371 PMC2988496

[52] Percudani R, Pavesi A, Ottonello S (1997) Transfer RNA gene redundancy and translational selection in Saccharomyces cerevisiae. J Mol Biol 268: 322–330. 9159473 10.1006/jmbi.1997.0942

[53] Karolchik D, Hinrichs AS, Furey TS, Roskin KM, Sugnet CW, et al (2004) The UCSC Table Browser data retrieval tool. Nucleic Acids Res 32: D493–D496. 14681465 10.1093/nar/gkh103 PMC308837

[54] R Development Core Team (2005) R: a language and environment for statistical computing. Vienna, Austria: R Foundation for Statistical Computing.

[55] Lu J, Deutsch C (2005) Folding zones inside the ribosomal exit tunnel. Nat Struct Mol Biol 12: 1123–1129. 16299515 10.1038/nsmb1021

[56] Yonath A, Leonard KR, Wittmann HG (1987) A tunnel in the large ribosomal subunit revealed by three-dimensional image reconstruction. Science 236: 813–816. 3576200 10.1126/science.3576200

[57] Cavener DR, Ray SC (1991) Eukaryotic start and stop translation sites. Nucleic Acids Res 19: 3185–3192. 1905801 10.1093/nar/19.12.3185 PMC328309

