# Supplementary material for: Positively Charged Residues Are the Major Determinants of Ribosomal Velocity
Source: PLoS Biol. 2013 Mar 12;11(3):e1001508. doi: 10.1371/journal.pbio.1001508 (PMC3595205; doi:10.1371/journal.pbio.1001508)
Supplement: Figure S1 — Figure 2 redone using rare codons defined according to genomic frequency shows rare codons do not slow ribosomes. In the main text, we investigate whether nonoptimal codons—that is, those with low tAI scores—might slow codons and find that they do not. To ensure that our finding that these “rare” codons do not slow ribosomes does not simply hinge on our definition of “rare,” we have repeated the analysis using an alternative definition. Here, we define “rare” codons according to their actual frequency in the genome as measured from our set of filtered genes. This rare set, of equal size to the rare tAI set, comprises the following codons: CGG, CGC, CGA, TGC, CCG, CTC, GGG, GCG, CGT, CCC, CAC, TGT, ACG, TCG, and AGG. We find that rare codons, where rare means genomically rare, do not slow ribosomes when in clusters (single rare codons; two rare codons anywhere within a five-codon stretch; three rare codons within eight codons; four or five within 10; and six or more within 16). Note slowing should be observed over, not after, the rare codon(s). (A) All genes with rare codon clusters. Regression of area under curve∼number of rare codons in cluster, slope = −0.79, p = 0.080. Regressions were performed as detailed in the main text (see Figure 1 for a description of the calculation of area under the curve). We note even if p were significant, the slope would be negative, whereas if rare codons did slow ribosomes, we should expect to see a positive slope. (B) Genes with rare codon clusters that have 0 or 1 positive charge coded for in the last 30 codon positions plotted. These plots represent the net effect of tAI on ribosomal density, with the bulk of the effect of positive charge removed. (C) Genes with rare codon clusters that have two or more positive charges in the last 30 codon positions plotted. (PDF) [file pbio.1001508.s001.pdf]

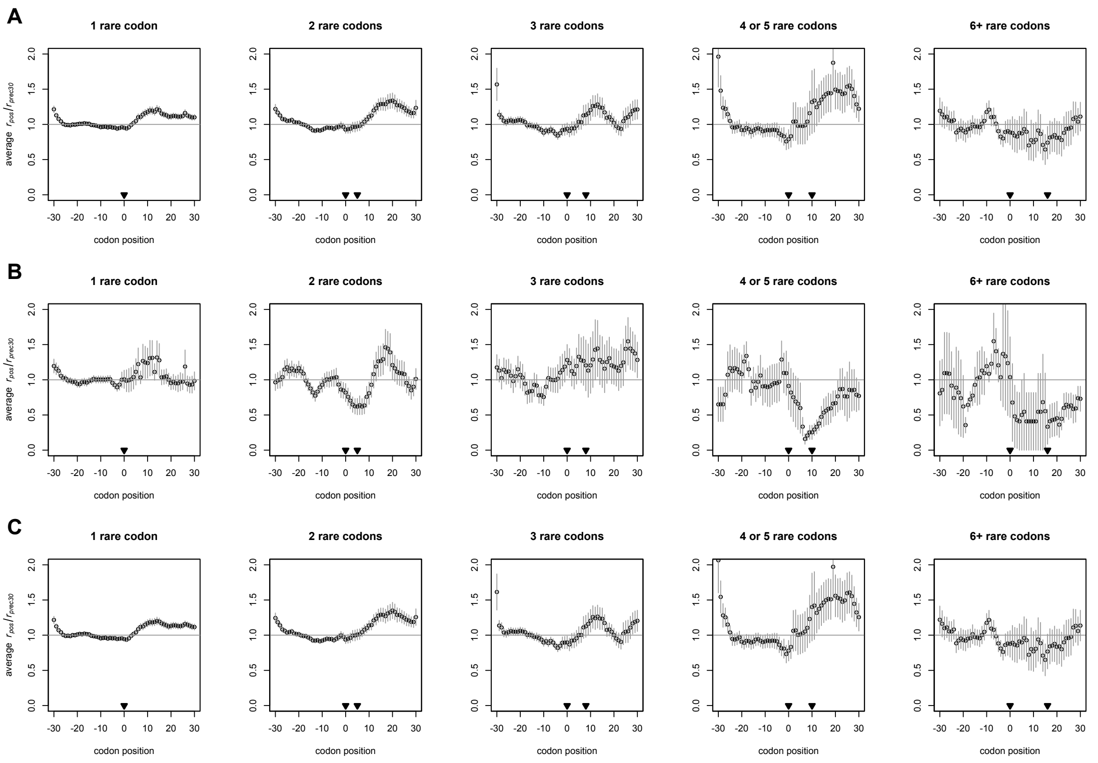

Figure S1. Figure 2 redone using rare codons defined according to genomic frequency shows rare codons do not slow ribosomes. In the main text we investigate whether non-optimal codons, i.e. those with low tAI scores, might slow codons and find that they do not. To ensure that our finding that these 'rare' codons do not slow ribosomes does not simply hinge on our definition of 'rare', we have repeated the analysis using an alternative definition. Here, we define 'rare' codons according to their actual frequency in the genome as measured from our set of filtered genes. This rare set, of equal size to the rare tAI set, comprises the following codons: CGG, CGC, CGA, TGC, CCG, CTC, GGG, GCG, CGT, CCC, CAC, TGT, ACG, TCG, AGG. We find that rare codons, where rare means genomically rare, do not slow ribosomes when in clusters (single rare codons; two rare codons anywhere within a 5-codon stretch; 3 rare codons within 8 codons; 4 or 5 within 10, and 6 or more within 16). Note slowing should be observed over, not after, the rare codon(s). **A)** All genes with rare codon clusters. Regression of *area under curve ~ number of rare codons in cluster*, slope = -0.79, P = 0.080. Regressions were performed as detailed in the main text (see Figure 1 for a description of the calculation of area under the curve). We note even if P were significant, the slope would be negative, whereas if rare codons did slow ribosomes we should expect to see a positive slope. **B)** Genes with rare codon clusters which have 0 or 1 positive charges coded for in the last 30 codon positions plotted. These plots represent the net effect of tAI on ribosomal density with the bulk of the effect of positive charge removed. **C)** Genes with rare codon clusters which have 2 or more positive charges in the last 30 codon positions plotted.
