# Supplementary material for: Positively Charged Residues Are the Major Determinants of Ribosomal Velocity
Source: PLoS Biol. 2013 Mar 12;11(3):e1001508. doi: 10.1371/journal.pbio.1001508 (PMC3595205; doi:10.1371/journal.pbio.1001508)
Supplement: Figure S2 — Consecutive rare codons, where rare is with reference to genomic frequency, do not slow ribosomes. In the main text, we investigate whether nonoptimal codons—that is, those with low tAI scores—might slow codons and find that they do not. To ensure that our finding that these “rare” codons do not slow ribosomes does not simply hinge on our definition of “rare,” we have repeated the analysis using an alternative definition. Here, we define “rare” codons according to their actual frequency in the genome as measured from our set of filtered genes. This rare set, of equal size to the rare tAI set, comprises the following codons: CGG, CGC, CGA, TGC, CCG, CTC, GGG, GCG, CGT, CCC, CAC, TGT, ACG, TCG, and AGG. The consecutive rare codons in considered codons are present between the first and second arrowheads. See Figure 1 for a description of the calculation of the area under the curve. (A) All genes with rare codon clusters. Regression of area under curve∼number of rare codons in cluster, slope = −9.8, p = 0.32. (B) Genes with rare codon clusters that have 0 or 1 positive charge coded for in the last 30 codon positions plotted. These plots represent the net effect of tAI on ribosomal density, with the bulk of the effect of positive charge removed. (C) Genes with rare codon clusters that have two or more positive charges in the last 30 plotted codon positions. (PDF) [file pbio.1001508.s002.pdf]

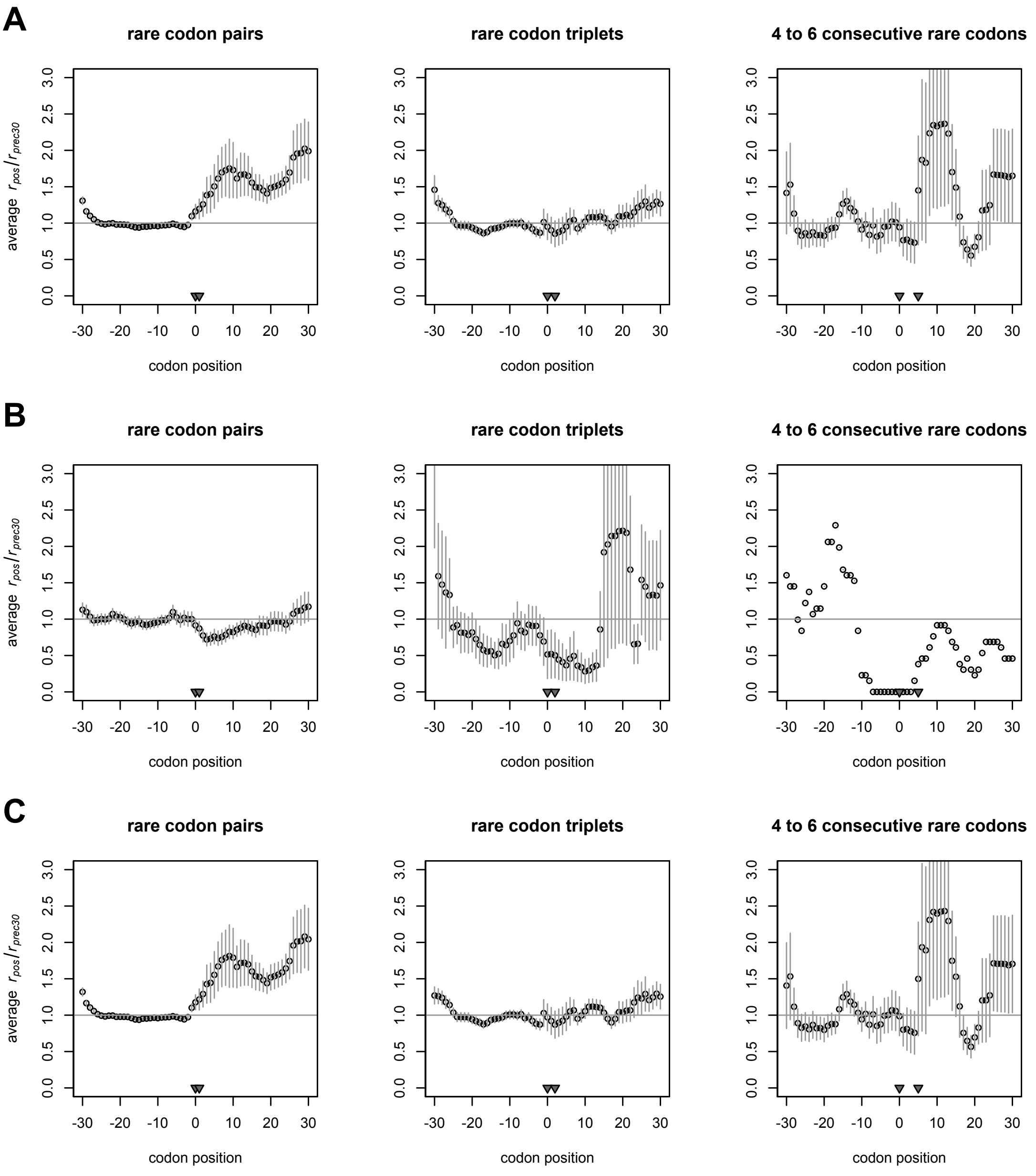

Figure S2. Consecutive rare codons, where rare is with reference to genomic frequency, do not slow ribosomes. In the main text we investigate whether non-optimal codons, i.e. those with low tAI scores, might slow codons and find that they do not. To ensure that our finding that these ‘rare’ codons do not slow ribosomes does not simply hinge on our definition of ‘rare’, we have repeated the analysis using an alternative definition. Here, we define ‘rare’ codons according to their actual frequency in the genome as measured from our set of filtered genes. This rare set, of equal size to the rare tAI set, comprises the following codons: CGG, CGC, CGA, TGC, CCG, CTC, GGG, GCG, CGT, CCC, CAC, TGT, ACG, TCG, AGG. The consecutive rare codons in considered codons are present between the first and second arrowheads. See Figure 1 for a description of the calculation of the area under the curve. **A)** All genes with rare codon clusters. Regression of *area under curve ~ number of rare codons in cluster*, slope = -9.8, P = 0.32. **B)** Genes with rare codon clusters which have 0 or 1 positive charges coded for in the last 30 codon positions plotted. These plots represent the net effect of tAI on ribosomal density with the bulk

of the effect of positive charge removed. **C)** Genes with rare codon clusters which have 2 or more positive charges in the last 30 plotted codon positions.
