# Supplementary material for: Positively Charged Residues Are the Major Determinants of Ribosomal Velocity
Source: PLoS Biol. 2013 Mar 12;11(3):e1001508. doi: 10.1371/journal.pbio.1001508 (PMC3595205; doi:10.1371/journal.pbio.1001508)
Supplement: Figure S3 — Codons that are overused in high-ribosomal occupancy windows are not “rare” according to genomic frequency. In some supplemental analyses, we examine whether “rare” codons slow ribosomes and define “rare” as the quartile of those most infrequent codons in the genome. To ensure there is not a problem with this definition, we have examined the difference in trends of codon usage at large between the two windows. (A) Tallies of all the codons used among the high-occupancy and low-occupancy windows within each gene (including the preceding five codons before each window) were kept separately. We plotted the counts for each codon in the high ribosomal occupancy window versus the counts in the low occupancy window and have color-coded the codons according to their frequency (see also Figure S6 for rare codons defined according to their tAI). If all codons are used equally among the slowly translated and quickly translated windows, then the regression should give a slope of 1, with all data points falling precisely upon the regression line. Since we have no prior expectation as to which variable should be on the x- versus y-axis—we are simply testing for a slope of 1—we used standardized major axis regression using the “smatr” package in R. We performed standardized major axis regressions of usage count(codon), high occupancy windows∼usage count(codon), low occupancy windows along with package tests that the slope of the line is 1 and that the intercept falls through 0. When we consider only those codons within the lowest quartile of frequency values, we find that the resulting regression has a slope not significantly different from 1 (p = 0.51) and an intercept not significantly different from 0 (p = 0.68), indicating that on the whole the rarest (tAI) quartile of codons are used equally between the slow and quickly translated windows. Considering all codons, however, gives a regression with both a slope different from 1 (p = 2.9e-04) and an intercept different from 0 (p [file pbio.1001508.s003.pdf]

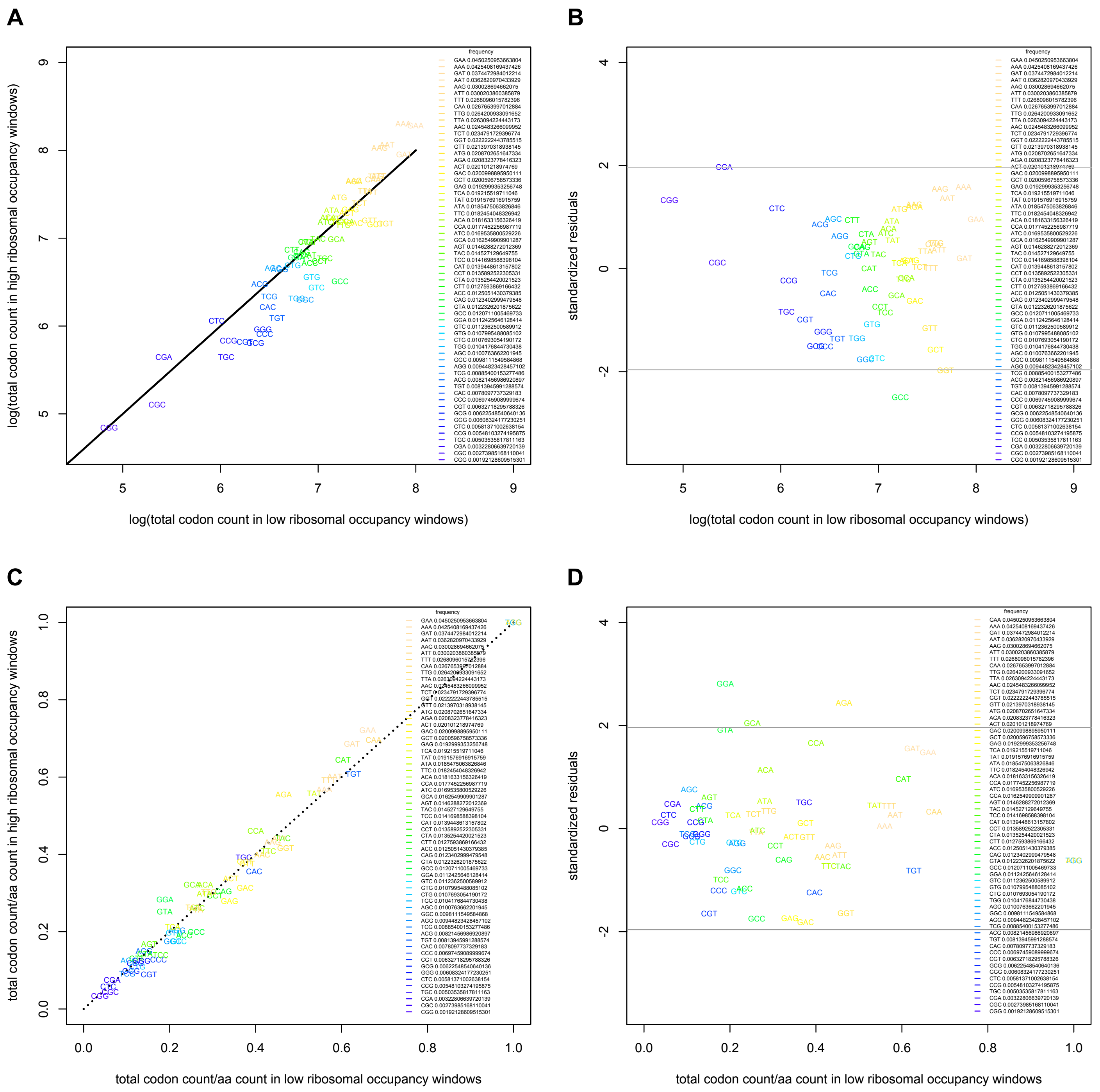

Figure S3. Codons which are overused in high-ribosomal occupancy windows are not ‘rare’ according to genomic frequency. In some supplemental analyses we examine whether ‘rare’ codons slow ribosomes, and define ‘rare’ as the quartile of those most infrequent codons in the genome. To ensure there is not a problem with this definition, we have examined the difference in trends of codon usage at large between the two windows. **A.** Tallies of all the codons used among the high-occupancy and low-occupancy windows within each gene (including the preceding 5 codons before each window) were kept separately. We plotted the counts for each codon in the high ribosomal occupancy window versus the counts in the low occupancy window, and have color-coded the codons according to their frequency (see also Figure S6 for rare codons defined according to their tAI). If all codons are used equally among the slowly-translated and quickly-translated windows then the regression should give a slope of 1, with all datapoints falling precisely upon the regression line. Since we have no prior expectation as to which variable should be on the *x*- vs. *y*-axis—we are simply testing for a slope of 1—we used standardized major axis regression using the ‘smatr’ package in R. We performed standardized major axis regressions of *usage count(codon), high occupancy windows ~ usage count(codon), low occupancy windows* along with package tests that the slope of the line is 1 and that the intercept falls through 0. When we consider only those codons within the lowest quartile of frequency values, we find that the resulting regression has a slope not significantly different from one (P = 0.51) and an intercept not significantly different from 0 (P

= 0.68), indicating that on the whole the rarest (tAI) quartile of codons are used equally between the slow and quickly-translated windows. Considering all codons, however, gives a regression with both a slope different from 1 (P = 2.9e-04) and an intercept different from 0 (P = 4.4e-04), corroborating that not rarer but more common codons are used more in the high-occupancy windows. The line $x = y$ is plotted just as a visual aid. **B.** An examination of the residuals from part A. Those codons which lie more than ~2 standard deviations away from the regression line are not from the rare end of the frequency spectrum but do tend to encode positively charged residues. Horizontals at $y = -1.96, +1.96$ are plotted. **C.** Given that there will of course be constraints on amino acid sequence, we also desire to investigate the differences in codon usage between the two windows given the protein-coding composition of each. All of the total codon counts for each the low-occupancy window (as described above) were divided by the total amino acid count encoded by that codon for the low-occupancy window. The same normalization was performed for the high-occupancy windows, and the normalized codon counts were then plotted against one another. Performing a standard major axis regression on the amino acid-adjusted codon counts shows that codons, given the protein coding sequence, are on the whole used proportionally between the quickly and slowly-translated windows. When we consider only those codons within the lowest quartile of frequency values, we find that the resulting regression has a slope not significantly different from one (P = 0.74) and an intercept not significantly different from 0 (P = 0.25), indicating that on the whole the rarest (frequency) quartile of codons are used equally between the slow and quickly-translated windows. Considering all codons, we find a slope significantly different from, but very close to, 1 (P =0.049; slope 95% CI of 1.00, 1.08) and an intercept not different from 0 (P = 0.10). The line $x = y$ is plotted as a visual aid. **D.** The finding in part C that codons, on the whole, are not used significantly differently between the slowly and quickly translated windows (given their respective amino acid compositions) is confirmed by an analysis of the residuals. The one codon which is possibly significantly over-used is does not have a low genomic frequency. Horizontals at $y = -1.96, +1.96$ are plotted.
