# Supplementary material for: Positively Charged Residues Are the Major Determinants of Ribosomal Velocity
Source: PLoS Biol. 2013 Mar 12;11(3):e1001508. doi: 10.1371/journal.pbio.1001508 (PMC3595205; doi:10.1371/journal.pbio.1001508)
Supplement: Figure S4 — Shifting the “preceding 30 codons” window 4 codons upstream to accommodate the “back” of the ribosome still shows rare codons do not slow ribosomes. Imagining ribosomes did stop at rare (tAI) codons, the A-site would still be ∼10–12 nucleotides from the end of the ribosomal footprint. To make sure we are not in fact improperly normalizing footprint counts around rare clusters by a “preceding 30” sequence that contains part of the footprints, we moved the “preceding 30 codons” window upstream by four codons (i.e., 12 nt). We achieve very similar results to those presented in the main text (see Figure 2). (A) All genes with rare codon clusters. (B) Genes with rare codon clusters that have 0 or 1 positive charge coded for in the last 30 codon positions plotted. These plots represent the net effect of tAI on ribosomal density, with the bulk of the effect of positive charge removed. (C) Genes with rare codon clusters that have two or more positive charges in the last 30 codon positions plotted. (PDF) [file pbio.1001508.s004.pdf]

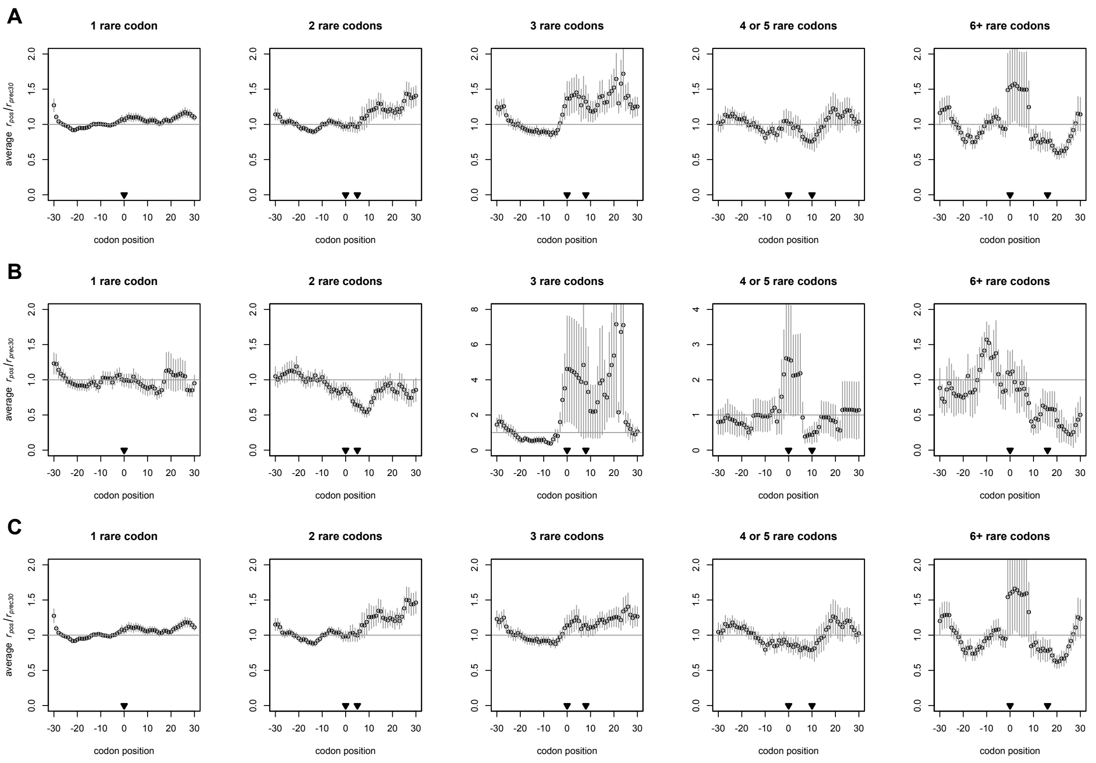

Figure S4. Shifting the 'preceding 30 codons' window 4 codons upstream to accommodate the 'back' of the ribosome still shows rare codons do not slow ribosomes. Imagining ribosomes did stop at rare (tAI) codons, the A-site would still be ~10-12 nucleotides from the end of the ribosomal footprint. To make sure we are not in fact improperly normalizing footprint counts around rare clusters by a 'preceding 30' sequence which contains part of the footprints, we moved the 'preceding 30 codons' window upstream by 4 codons (i.e. 12 nt). We achieve very similar results to those presented in the main text (see Figure 2). **A)** All genes with rare codon clusters. **B)** Genes with rare codon clusters which have 0 or 1 positive charges coded for in the last 30 codon positions plotted. These plots represent the net effect of tAI on ribosomal density with the bulk of the effect of positive charge removed. **C)** Genes with rare codon clusters which have 2 or more positive charges in the last 30 codon positions plotted.
