# Supplementary material for: Positively Charged Residues Are the Major Determinants of Ribosomal Velocity
Source: PLoS Biol. 2013 Mar 12;11(3):e1001508. doi: 10.1371/journal.pbio.1001508 (PMC3595205; doi:10.1371/journal.pbio.1001508)
Supplement: Figure S5 — Pairs, triplets, etc. of rare (low tAI) codons do not tend to slow ribosomes. The consecutive rare codons in considered codons are present between the first and second arrowheads. The mean rpos/rprec30, or relative change in ribosomal occupancy, at each position across aligned transcripts ± s.e.m. is plotted. The horizontal at y = 1 represents the null expectation that positive charges do not alter ribosomal speed—that is, that ribosomes are, on average, as frequently present before the rare codon cluster as after it. (A) All genes with rare codon clusters. (B) Genes with rare codon clusters that have 0 or 1 positive charge coded for in the last 30 codon positions plotted. These plots represent the net effect of tAI on ribosomal density, with the bulk of the effect of positive charges removed. (C) Genes with rare codon clusters that have two or more positive charges in the last 30 plotted codon positions. (PDF) [file pbio.1001508.s005.pdf]

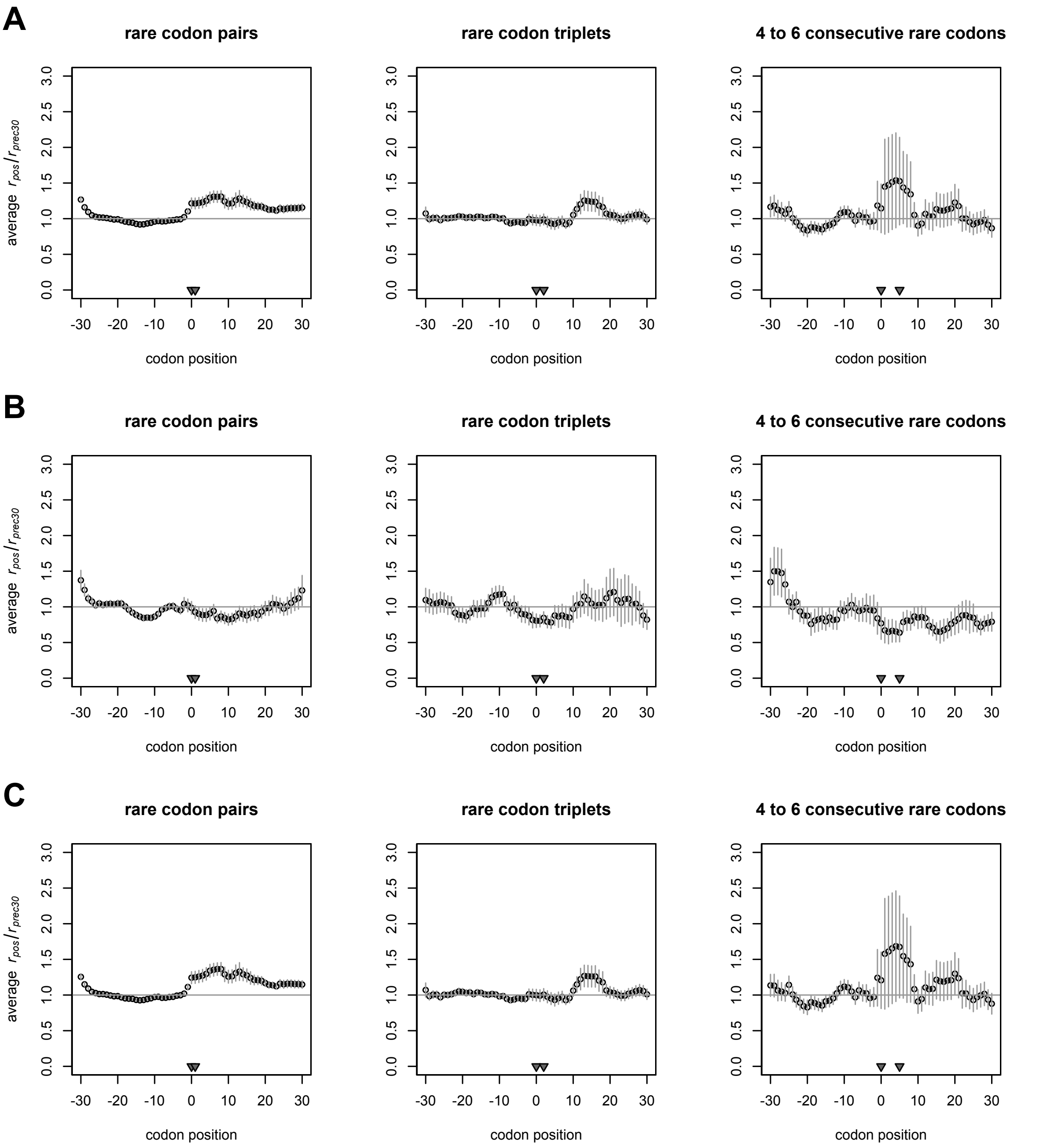

Figure S5. Pairs, triplets etc. of rare (low tAI) codons do not tend to slow ribosomes. The consecutive rare codons in considered codons are present between the first and second arrowheads. The mean r_{pos}/r_{prec30}, or relative change in ribosomal occupancy, at each position across aligned transcripts ± s.e.m. is plotted. The horizontal at y = 1 represents the null expectation that positive charges do not alter ribosomal speed, i.e. that ribosomes are, on average, as frequently present before the rare codon cluster as after it. **A)** All genes with rare codon clusters. **B)** Genes with rare codon clusters which have 0 or 1 positive charges coded for in the last 30 codon positions plotted. These plots represent the net effect of tAI on ribosomal density with the bulk of the effect of positive charge removed. **C)** Genes with rare codon clusters which have 2 or more positive charges in the last 30 plotted codon positions.
