# Supplementary material for: Positively Charged Residues Are the Major Determinants of Ribosomal Velocity
Source: PLoS Biol. 2013 Mar 12;11(3):e1001508. doi: 10.1371/journal.pbio.1001508 (PMC3595205; doi:10.1371/journal.pbio.1001508)
Supplement: Figure S7 — Similarity to Kozak sequence is not the primary cause of ribosomal slowing. Given that transcript similarity to the Shine-Dalgarno sequence has been shown to slow ribosomes in bacteria due to interactions of the sequence with components of the ribosomal RNA [17], we wondered whether translation speed in yeast might not be modulated by codon usage per se but by the ability of ribosomes to bind to transcript sequence that mirrors the eukaryotic Kozak sequence. Specifically, we wanted to determine whether codons that are in high-ribosomal occupancy windows within a gene might be more likely to correspond to the Kozak sequence (as compared to codons in low-occupancy windows within the same genes) and hence bind ribosomes, slowing translation. We first determined which codons were enriched in the Kozak sequence relative to the codon frequencies seen throughout the yeast genome at large using a simple randomization. Nucleotide frequencies at each position of the Kozak sequence in yeast were taken from Cavener and Ray 1991 [57]. To determine the frequencies of all the possible “codons” among the Kozak sequence space, we randomly created 20,000 possible Kozak sequences from the delineated nucleotide frequencies at each site in the consensus sequence. We then counted all possible triplet “codons” within each sequence, regardless of reading frame (since we assume that as the ribosome traverses RNA, it may bind the Kozak sequence regardless of the surrounding reading frame). The counts of all possible RNA triplets that we observe within our simulated sequences are the observed “codons” within the Kozak sequence. In order to determine whether or not certain codons are over- or underused in the Kozak sequence, we compare them to the counts of codons observed (again in any reading frame) across 20,000 randomized sequences derived from the basal codon frequencies in the S. cerevisiae genome and of the same length as the Kozak sequence. We calculate Z, a measure of the over- or und [file pbio.1001508.s007.pdf]

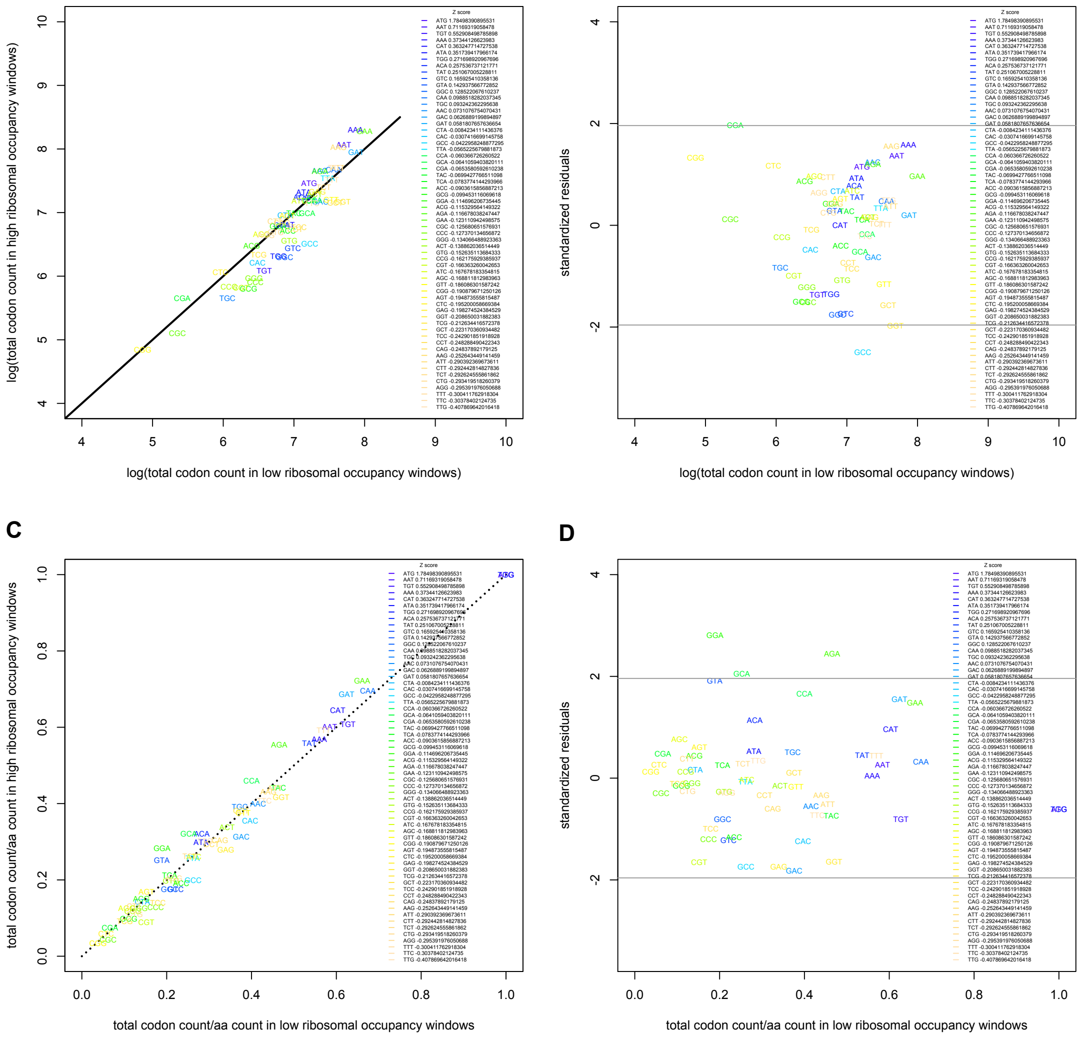

Figure S7. Similarity to Kozak sequence is not the primary cause of ribosomal slowing. Given that transcript similarity to the Shine-Dalgarno sequence has been shown to slow ribosomes in bacteria due to interactions of the sequence with components of the ribosomal RNA [17], we wondered whether translation speed in yeast might not be modulated by codon usage per se but by the ability of ribosomes to bind to transcript sequence which mirrors the eukaryotic Kozak sequence. Specifically, we wanted to determine whether codons which are in high-ribosomal occupancy windows within a gene might be more likely to correspond to the Kozak sequence (as compared to codons in low-occupancy windows within the same genes) and hence bind ribosomes, slowing translation. We first determined which codons were enriched in the Kozak sequence relative to the codon frequencies seen throughout the yeast genome at large using a simple randomization. Nucleotide frequencies at each position of the Kozak sequence in yeast were taken from Cavener and Ray 1991 [57]. To determine the frequencies of all the possible ‘codons’ among the Kozak sequence space, we randomly created 20000 possible Kozak sequences from the delineated nucleotide frequencies at each site in the consensus sequence. We then counted all possible triplet ‘codons’ within each sequence, regardless of reading frame (since we assume that as the ribosome traverses RNA, it may bind the Kozak sequence regardless of the surrounding reading frame). The counts of all possible

RNA triplets that we observe within our simulated sequences are the observed 'codons' within the Kozak sequence. In order to determine whether or not certain codons are over- or under-used in the Kozak sequence, we compare them to the counts of codons observed (again in any reading frame) across 20000 randomized sequences derived from the basal codon frequencies in the S. cerevisiae genome and of the same length as the Kozak sequence. We calculate Z, a measure of the over- or under-usage of a particular codon within the Kozak sequence (as compared to the rest of the genome) as Z_{codon} = [Observed codon count (in Kozak sequence) – Expected count (from genome frequencies)] / Expected SD of codon. We can then examine which codons are over-used (i.e. with a positive Z-score) in slowly-translated windows relative to quickly-translated windows in the same genes and ask if these codons are overrepresented among the Kozak sequence(s). If so, this would suggest that RNA sequence may be slowing ribosomes not through codon:anticodon interactions but by Kozak-similar sequences binding the ribosome. **A.** Tallies of all the codons used among the high-occupancy and low-occupancy windows were kept separately. We then performed a regression of count(codon) in high occupancy windows ~ count(codon) in low occupancy windows. The line y = x is plotted as a visual aid. **B.** Standardized residuals from the analysis in part A are plotted against the original x values in A. No codons which are over-represented in the Kozak sequence (i.e. have positive Z-scores) have standardized residuals greater than +1.96, implying they may be overused. The high-Z codon AAA comes close to the + 1.96 mark, however we note that AAA encodes a positively charged amino acid, lysine, as do AAG and CGA which also fall near the +1.96 mark and are not overused in the Kozak sequence. Horizontals are plotted at y = 1.96, +1.96. **C.** Here the codon counts used in part A were normalized by the usage of the corresponding amino acid to investigate fluctuations in synonymous codon choice given the amino acid in the protein. We then performed a regression of count(codon) / count(corresponding amino acid) in high occupancy windows ~ count(codon) / count(corresponding amino acid) in low occupancy windows. The line y = x is plotted as a visual aid. **D.** Standardized residuals from C are plotted against the original x values. We observe that those codons which are significantly over-represented (i.e. over +1.96 standard deviations) in the high occupancy windows (given the amino acid content) are in fact under-represented in the Kozak sequence (with a negative Z-score) compared to the genome at large. Even the AAA codon, above the +1.96 standard deviation mark in part B, is not over-used when factoring in amino acid choice as shown here. We consider this confirmation of our inference that the AAA codon has a high residual in part B on account of the amino acid it encodes, and not merely because of its similarity to Kozak sequence. For these reasons, although we cannot rule out a potential contribution to slowing, we consider that transcript similarity to the Kozak sequence cannot explain the bulk of ribosomal pausing in yeast.
