# Supplementary material for: Positively Charged Residues Are the Major Determinants of Ribosomal Velocity
Source: PLoS Biol. 2013 Mar 12;11(3):e1001508. doi: 10.1371/journal.pbio.1001508 (PMC3595205; doi:10.1371/journal.pbio.1001508)
Supplement: Figure S8 — Ribosomal slowing after positive charge clusters in the ribosomal footprint set taken from amino acid-starved yeast [29]. (PDF) [file pbio.1001508.s008.pdf]

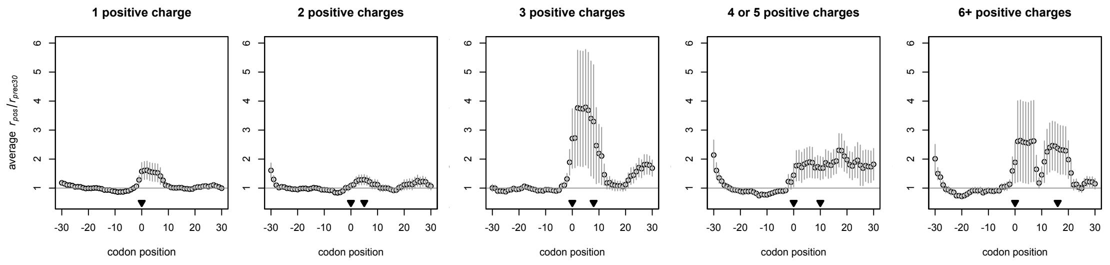

Figure S8. Ribosomal slowing after positive charge clusters in the ribosomal footprint set taken from amino acid-starved yeast [29]**.**
