# Supplementary material for: Positively Charged Residues Are the Major Determinants of Ribosomal Velocity
Source: PLoS Biol. 2013 Mar 12;11(3):e1001508. doi: 10.1371/journal.pbio.1001508 (PMC3595205; doi:10.1371/journal.pbio.1001508)
Supplement: Figure S9 — Changes in relative translation rates after rare codon clusters calculated from amino-acid-starved data [29]. Three rare codon clusters are plotted with outlier axes. (A) All genes with rare codon clusters. (B) Genes with rare codon clusters that have 0 or 1 positive charge coded for in the last 30 codon positions plotted. These plots represent the net effect of tAI on ribosomal density with the bulk of the effect of positive charge removed. (C) Genes with rare codon clusters that have two or more positive charges in the last 30 codon positions plotted. (PDF) [file pbio.1001508.s009.pdf]

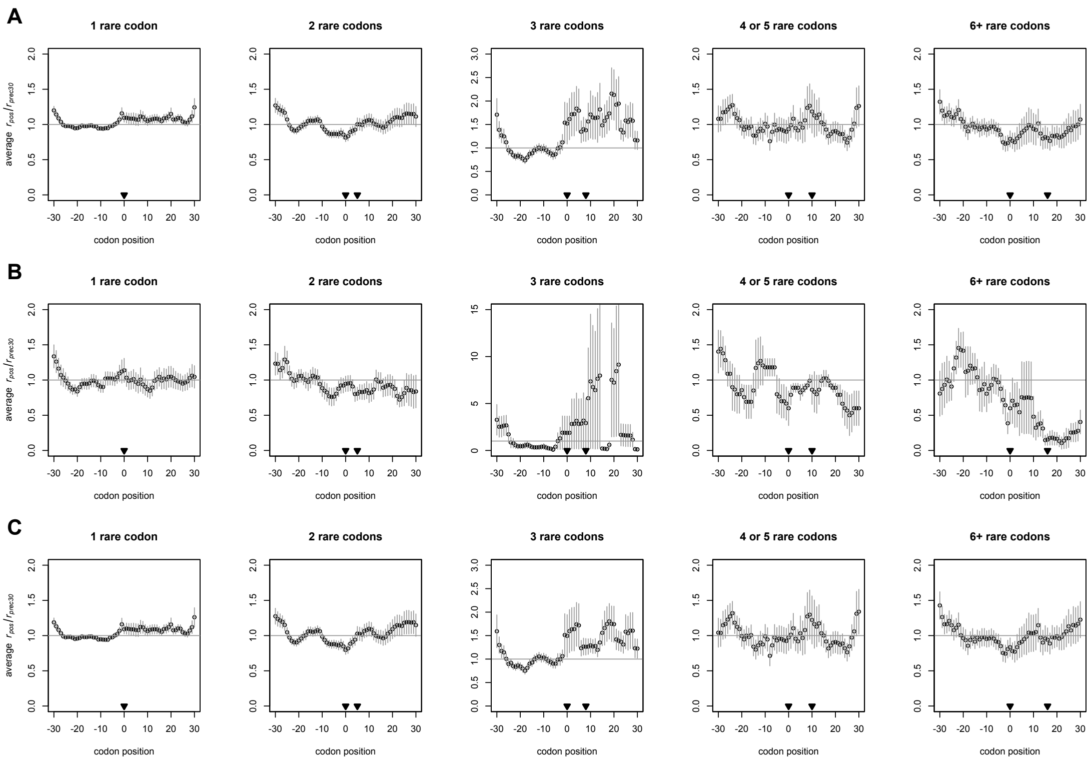

Figure S9. Changes in relative translation rates after rare codon clusters calculated from amino acid-starved data [29]**.** 3 rare codon clusters are plotted with outlier axes. **A)** All genes with rare codon clusters. **B)** Genes with rare codon clusters which have 0 or 1 positive charges coded for in the last 30 codon positions plotted. These plots represent the net effect of tAI on ribosomal density with the bulk of the effect of positive charge removed. **C)** Genes with rare codon clusters which have 2 or more positive charges in the last 30 codon positions plotted.
