# Supplementary material for: Positively Charged Residues Are the Major Determinants of Ribosomal Velocity
Source: PLoS Biol. 2013 Mar 12;11(3):e1001508. doi: 10.1371/journal.pbio.1001508 (PMC3595205; doi:10.1371/journal.pbio.1001508)
Supplement: Figure S10 — Positive charges show an additive (linear) trend in slowing ribosomes in the amino-acid-starved dataset [29], but rare codons do not. The degree of slowing is a function of both the magnitude of ribosomal density and the length of transcript the slowing covers. Therefore to measure any trend in the ability of either positive charges or codon clusters to slowing, the area between the curves depicting the average relative change in ribosomal density (rpos/rprec30) and the y = 1 null in Figures S8 and S9, whether positive or negative, was summed between x = 0 (the beginning of the cluster) and the point where the plotted values intersect with y = 1 again (see Figure 1). A positive value for the area under the curve indicates ribosomal slowing, while a negative value reflects faster movement. (A) Regression of area under curve∼size of cluster +0 gives a slope of 5.15 (p = 0.0122, r 2 = 0.7815). A linear model (not shown) that does not force the regression through the origin gives an insignificant intercept (p = 0.64). (B–D) Regression of area under curve∼size of cluster + 0, slope p = 0.56, 0.93, and 0.55, respectively. (PDF) [file pbio.1001508.s010.pdf]

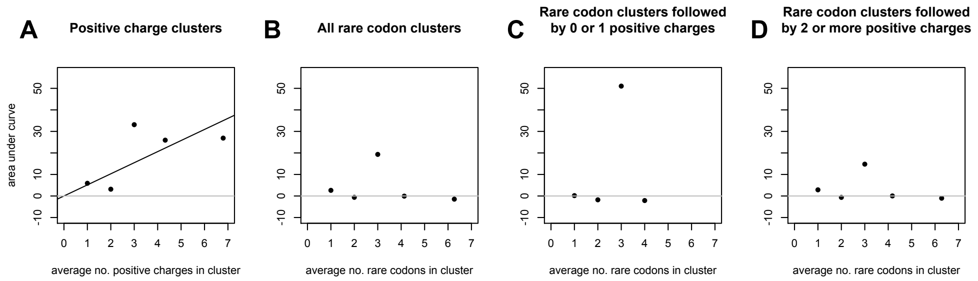

Figure S10. Positive charges show an additive (linear) trend in slowing ribosomes in the amino acid-starved dataset [29]**, but rare codons do not.** The degree of slowing is a function of both the magnitude of ribosomal density and the length of transcript the slowing covers. Therefore to measure any trend in the ability of either positive charges or codon clusters to slowing, the area between the curves depicting the average relative change in ribosomal density (r_{pos}/r_{prec30}) and the y=1 null in Figures S8 and S9, whether positive or negative, was summed between x=0 (the beginning of the cluster) and the point where the plotted values intersect with y=1 again (see Figure 1). A positive value for the area under the curve indicates ribosomal slowing, while a negative value reflects faster movement. **A)** Regression of *area under curve ~ size of cluster +0* gives a slope of 5.15 (P = 0.0122, r^2=0. 0.7815). A linear model (not shown) that does not force the regression through the origin gives an insignificant intercept (P=0.64). **B, C, D)** Regression of *area under curve ~ size of cluster + 0,* slope P = 0.56, 0.93, 0.55, respectively.
