# Supplementary material for: Positively Charged Residues Are the Major Determinants of Ribosomal Velocity
Source: PLoS Biol. 2013 Mar 12;11(3):e1001508. doi: 10.1371/journal.pbio.1001508 (PMC3595205; doi:10.1371/journal.pbio.1001508)
Supplement: Figure S11 — Positive charges encoded by A/G- and C-rich codons both slow ribosomes. If positive charges indeed slow codons, we should detect slowing regardless of the codon encoding the charge. Since we are now considering specific subgroups among the positive charge clusters depending on the corresponding codon composition, sample size quickly becomes an issue. The 1-positive charge clusters give not only the best sample size, but also the fairest comparison since the composition of the “cluster” must be binary (either A/G- or C-rich) and not mixed. Our results show that positive charge slows ribosomes regardless of the nature of the codon encoding the charge. The C-rich codons (encoding Arg and His) may slow translation slightly less than the A-rich codons (Lys and Arg). This is to be expected, as histidine has a lesser tendency to be charged at physiological pH (see also Results). (PDF) [file pbio.1001508.s011.pdf]

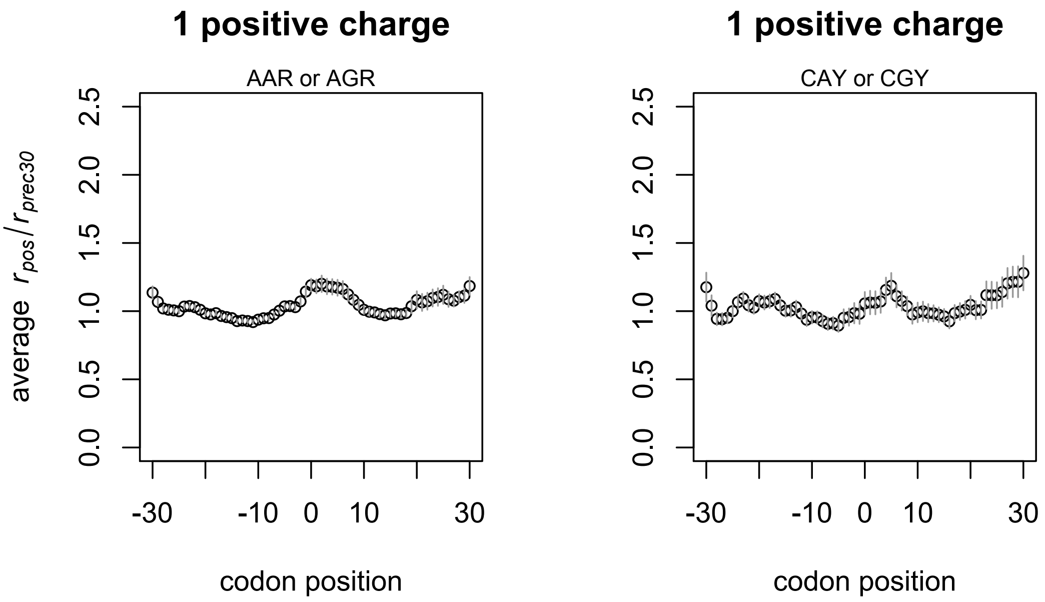

Figure S11. Positive charges encoded by A/G- and C-rich codons both slow ribosomes. If positive charges indeed slow codons, we should detect slowing regardless of the codon encoding the charge. Since we are now considering specific subgroups among the positive charge clusters depending on the corresponding codon composition, sample size quickly becomes an issue. The 1-positive charge clusters give not only the best sample size but also the fairest comparison since the composition of the 'cluster' must be binary (either A/G- or C-rich) and not mixed. Our results show that positive charge slows ribosomes regardless of the nature of the codon encoding the charge. The C-rich codons (encoding Arg and His) may slow translation slightly less than the A-rich codons (Lys and Arg). This is to be expected as histidine has a lesser tendency to be charged at physiological pH (see also Results).
