# Supplementary material for: Positively Charged Residues Are the Major Determinants of Ribosomal Velocity
Source: PLoS Biol. 2013 Mar 12;11(3):e1001508. doi: 10.1371/journal.pbio.1001508 (PMC3595205; doi:10.1371/journal.pbio.1001508)
Supplement: Figure S12 — Histidine-enriched clusters slow less than histidine-free clusters. As we note in the main text, histidine is less likely to be charged at physiological pH than lysine or arginine. Here we divide positive charge clusters according to whether or not they contain a minimal number of histidine residues versus no histidines at all and observe that greater slowing is observed after histidine-free clusters, in line with expectations if charge does slow ribosomes. (PDF) [file pbio.1001508.s012.pdf]

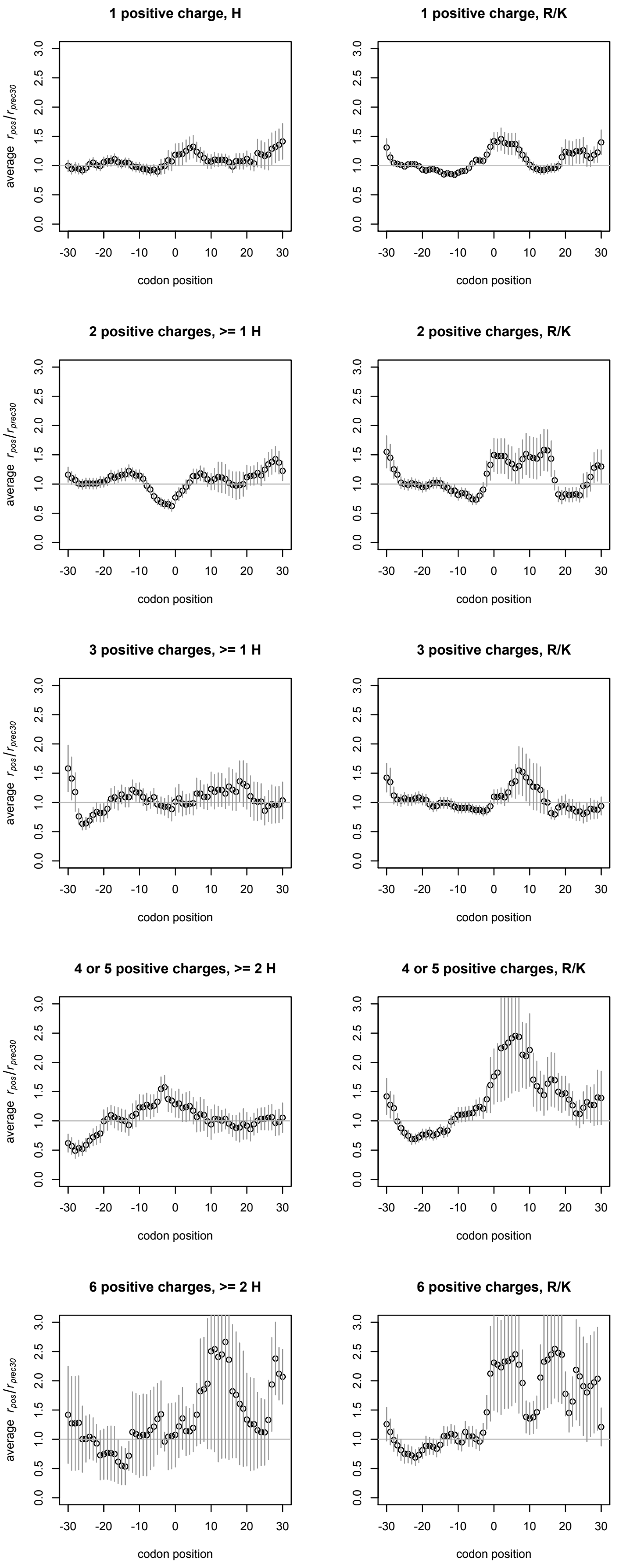

Figure S12. Histidine-enriched clusters slow less than histidine-free clusters. As we note in the main text, histidine is less likely to be charged at physiological pH than

lysine or arginine. Here we divide positive charge clusters according to whether or not they contain a minimal number of histidine residues versus no histidines at all and observe that greater slowing is observed after histidine-free clusters, in line with expectations if charge does slow ribosomes.
