# Supplementary material for: Positively Charged Residues Are the Major Determinants of Ribosomal Velocity
Source: PLoS Biol. 2013 Mar 12;11(3):e1001508. doi: 10.1371/journal.pbio.1001508 (PMC3595205; doi:10.1371/journal.pbio.1001508)
Supplement: Figure S13 — The only significantly overused amino acid in the high-ribosomal occupancy windows across genes (relative to the amino acid content in the paired low-occupancy windows in the same genes) is lysine, which is positively charged. In our main analysis we identified amino acids we expect to slow ribosomes (e.g., basic amino acids) and then examining the change in ribosomal occupancy upon their addition to the peptide chain. An alternative approach is to ask which amino acids are statistically overrepresented within the most slowly translated (i.e., most footprint-dense) regions within a gene. As different genes have their own expression levels, nucleotide contents, and functions, we would ideally like to control for these differences among genes when examining which amino acids are overused on the whole. For this reason, we re-employed a two-window analysis in which the highest ribosomal occupancy window and the lowest occupancy window (each of 10 codons) were identified in every gene for which we had ribosomal occupancy data. Tallies of all the amino acids used among the high-occupancy and low-occupancy windows (and including the preceding five codons before each window, as these amino acids may have just entered the tunnel when slowing occurs) were kept separately. We then performed a regression of usage count(aa), high occupancy windows∼usage count(aa), low occupancy windows: if all amino acids are used equally among the slowly translated and quickly translated windows, then the regression should give a slope of 1, with all data points falling precisely upon the regression line. We plotted the residuals of this regression against the low window count, such that amino acids that are significantly overused in the high-occupancy window will have standardized residuals of greater than +1.96. Only a positively charged amino acid (lysine) is significantly overused in the higher ribosomal occupancy window. (PDF) [file pbio.1001508.s013.pdf]

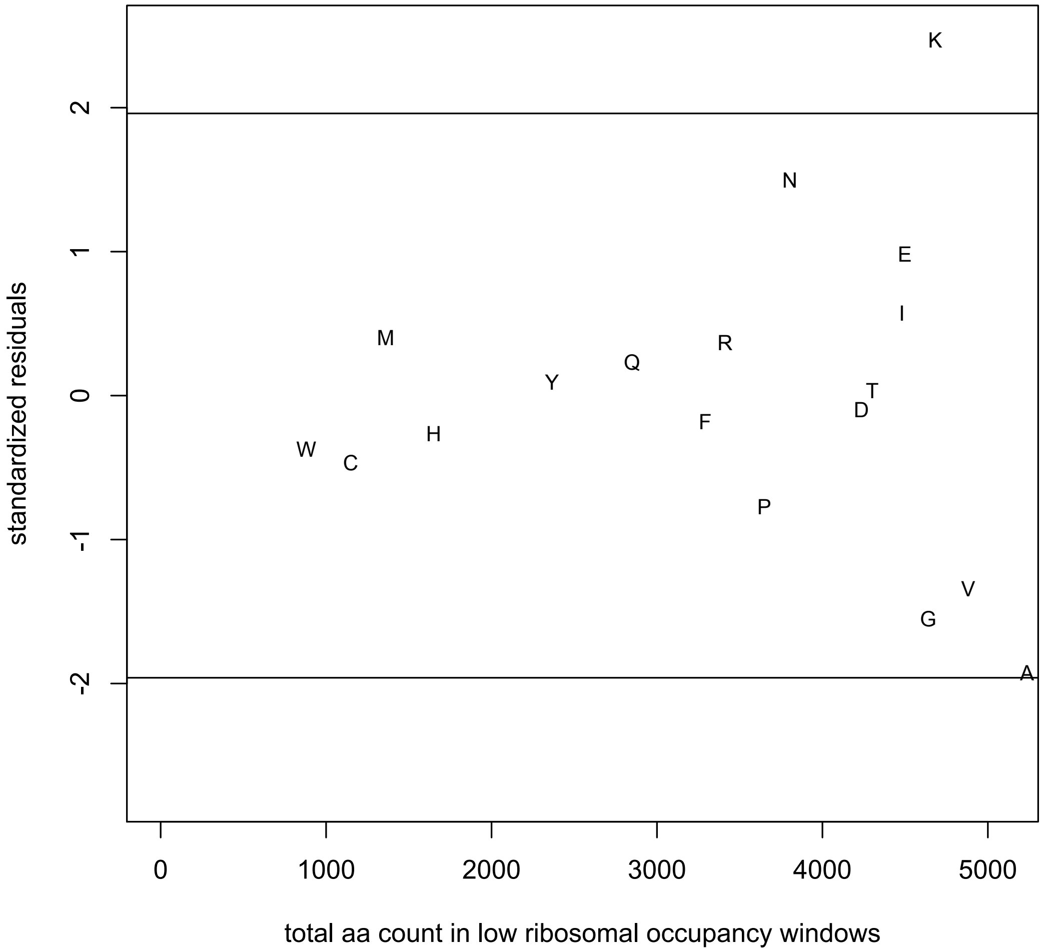

Figure S13. The only significantly overused amino acid in the high-ribosomal occupancy windows across genes (relative to the amino acid content in the paired low-occupancy windows in the same genes) is lysine, which is positively charged. In our main analysis we identified amino acids we expect to slow ribosomes (e.g. basic amino acids) and then examining the change in ribosomal occupancy upon their addition to the peptide chain. An alternative approach is to ask which amino acids are statistically overrepresented within the most slowly translated (i.e. most footprint-dense) regions within a gene. As different genes have their own expression levels, nucleotide contents, and functions, we would ideally like to control for these differences among genes when examining which amino acids are overused on the whole. For this reason we re-employed a two-window analysis in which the highest ribosomal occupancy window and the lowest-occupancy window (each of 10 codons) were identified in every gene for which we had ribosomal occupancy data. Tallies of all the amino acids used among the high-occupancy and low-occupancy windows (and including the preceding 5 codons before each window, as these amino acids may have just entered the tunnel when slowing occurs) were kept separately. We then performed a regression of *usage count(aa), high occupancy windows ~ usage count(aa), low occupancy windows*: if all amino acids are used equally among the slowly-translated and quickly-translated windows then the regression should give a slope of 1, with all datapoints falling precisely upon the regression line. We plotted

the residuals of this regression against the low window count, such that amino acids which are significantly overused in the high-occupancy window will have standardized residuals of greater than +1.96. Only a positively-charged amino acid (lysine) is significantly overused in the higher ribosomal occupancy window.
