# Supplementary material for: Positively Charged Residues Are the Major Determinants of Ribosomal Velocity
Source: PLoS Biol. 2013 Mar 12;11(3):e1001508. doi: 10.1371/journal.pbio.1001508 (PMC3595205; doi:10.1371/journal.pbio.1001508)
Supplement: Figure S14 — The effect of positive charge is not explained by covariance with codon usage or mRNA folding. In order to determine if global patterns of codon usage or mRNA secondary structure might in fact be contributing to patterns in ribosomal slowing we observe after clusters of positive charges, we also examined the relative changes in tAI and PARS values after the clusters. Within a given transcript, the relative increase or decrease in codon optimality at each position surrounding the charged cluster was calculated by dividing the measured ribosomal density at some codon position (tAIpos) (i.e., at some position before/after the charged residue is added) by the average tAI of the 30 codons preceding the first coded-for charge in the cluster within that transcript (tAIprec30). The mean relative change in tAI after a cluster positive charges was then calculated by aligning all transcripts with a given cluster size by the first charge in each cluster and calculating the average ratio (tAIpos/tAIprec30) in each codon site surrounding the cluster. We similarly calculated the relative increase or decrease in propensity for double-stranded structure, as quantified by PARS values, at each position surrounding the charged cluster. As PARS values as originally published [38] are logged ratios, we first took the antilog of all PARS values (making all of them positive) in order to be able to calculate relative increases or decreases in the values along transcripts by dividing the antilogged PARS value at some codon position surrounding the encoded charge cluster (PARSpos) by the average PARS of the 30 codons (all previously antilogged) preceding the first coded-for charge in the cluster within that transcript (PARSprec30). This method is conservative, as taking the antilog will result in PARS values indicating single-strandedness being sandwiched between 0 and 1, but with PARS values indicating double-strandedness spread above 1. Hence increases in double-stranded propensity will be [file pbio.1001508.s014.pdf]

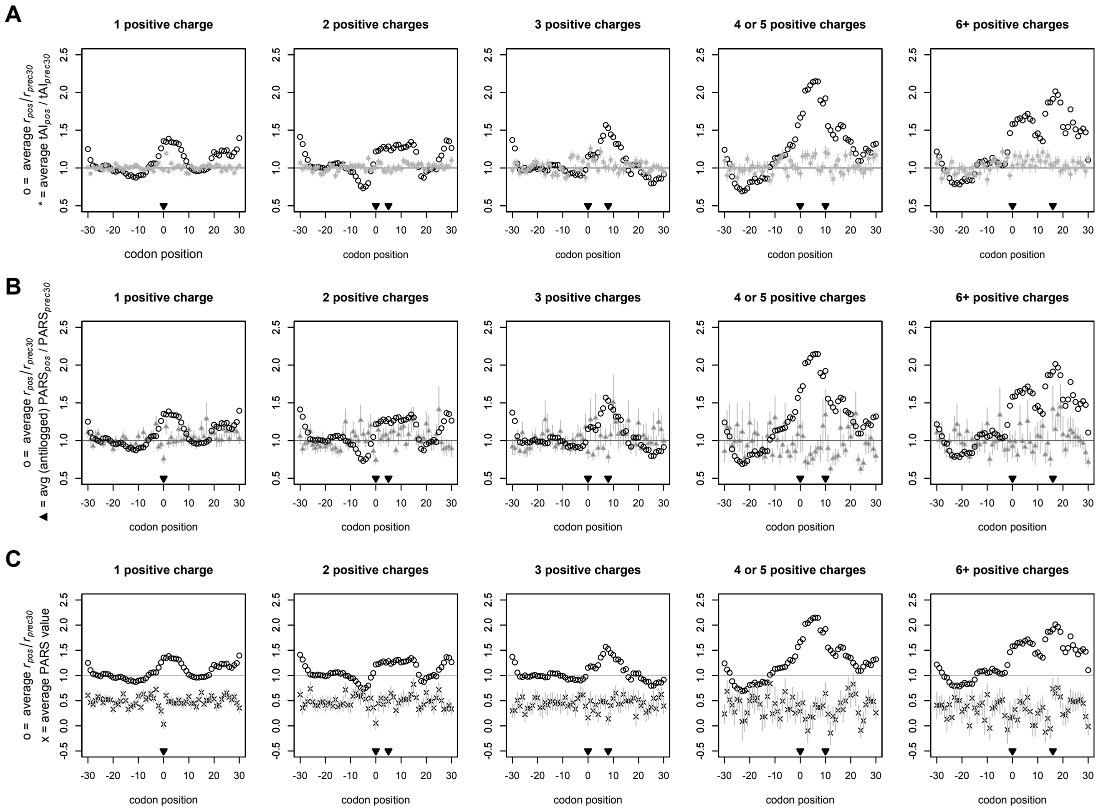

Figure S14. The effect of positive charge is not explained by covariance with codon usage or mRNA folding. In order to determine if global patterns of codon usage or mRNA secondary structure might in fact be contributing to patterns in ribosomal slowing we observe after clusters of positive charges, we also examined the relative changes in tAI and PARS values after the clusters. Within a given transcript, the relative increase or decrease in codon optimality at each position surrounding the charged cluster was calculated by dividing the measured ribosomal density at some codon position (tAI_{pos}) (i.e., at some position before/after the charged residue is added) by the average tAI of the thirty codons preceding the first coded-for charge in the cluster within that transcript (tAI_{prec30}). The mean relative change in tAI after a cluster positive charges was then calculated by aligning all transcripts with a given cluster size by the first charge in each cluster and calculating the average ratio (tAI_{pos}/tAI_{prec30}) in each codon site surrounding the cluster. We similarly calculated the relative increase or decrease in propensity for double-stranded structure, as quantified by PARS values, at each position surrounding the charged cluster. As PARS values as originally published [38] are logged ratios, we first took the antilog of all PARS values (making all of them positive) in order to be able to calculate relative increases or decreases in the values along transcripts by dividing the antilogged PARS value at some codon position surrounding the encoded charge cluster ($PARS_{pos}$) by the average PARS of the thirty codons (all previously antilogged) preceding the first coded-for charge in the cluster within that transcript ($PARS_{prec30}$). This method is conservative as taking the antilog will result in PARS values indicating single-strandedness being sandwiched between 0 and 1, but with PARS values indicating double-strandedness spread above 1. Hence increases in double-stranded propensity will be exaggerated. The average relative change in either tAI or PARS (mean tAI_{pos}/tAI_{prec30} or $PARS_{pos}/PARS_{prec30}$) at a given position after a cluster was then calculated by aligning all identified regions of a given cluster size

according to the first charge present in each cluster and calculating the average ratio in positions increasingly distant from the first positive charge of the aligned clusters. Positive charges in a cluster may be coded for anywhere between the two downturned triangles. An average r_{pos}/r_{prec30} above one indicates a relative local increase in ribosomal density in that position across transcripts (as in Fig. 1). **A.** An average tAI_{pos}/tAI_{prec30} below one indicates the codons in that position across transcripts tend to decrease in optimality on average relative to the average tAI of the preceding 30 codons across transcripts, while a ratio above one signifies an increase in optimality. We find that differential codon use in the vicinity of positive charges cannot explain the charge slowing effect. We observe no correlation between relative changes in ribosomal density and tAI after the first charge in the cluster ($0 \geq x \leq 30$ in this Figure, part A; Spearman P, left to right: 0.93, 0.73, 0.22, 0.17, 0.65). For a more relaxed test we then compared, for each plot in Fig. 5, the relative changes in codon optimality (tAI_{pos}/tAI_{prec30}) seen after the start of each cluster at x=0 until the point where relative change in ribosomal density (r_{pos}/r_{prec30}) drops back to previous levels (y = 1) to the tAI_{pos}/tAI_{prec30} values seen in all other surrounding plotted sites (i.e. those sites lacking charge-induced pausing). If anything, relatively more optimal ($tAI_{pos}/tAI_{prec30} > 1$) codons are coded for during periods of elevated ribosomal occupancy for clusters comprising 6 or more encoded cations, while no difference in optimality is detected in codon usage during elevated ribosomal occupancy compared to surrounding codon usage for other-sized charge clusters (Mann Whitney U-test P values, left to right in this Figure, part A: 0.96, 0.20, 0.07, 0.07, 0.003). Hence we conclude that changes in codon bias are not responsible for the slowing patterns associated with positively charged residues (Fig. 5), as expected if rare codons do not slow ribosomes (Fig 3A,B). **B.** An average relative change in (here antilogged, see Methods) PARS values (i.e. $PARS_{pos}/PARS_{prec30}$) plotted above one indicates a greater likelihood of double-stranded structure in that position on average relative to preceding sequence, while a ratio less than one indicates a decrease in propensity for double-strandedness relative to the preceding 30 codons. We find that the slowing effect of positive charge cannot be explained by mRNA folding in the vicinity of positive charges. There is no correlation between the relative change in PARS values ($PARS_{pos}/PARS_{prec30}$) after the first charge in the cluster (this Figure, part B, $0 \geq x \leq 30$) and relative changes in ribosomal density (Spearman P, left to right: 0.44, 0.68, 0.97, 0.99, 0.15), which we may have expected to observe if RNA structure has a local effect on ribosomal slowing. Likewise, under such a local-slowing hypothesis, we should expect to see a significant difference in the average PARS ratios seen amongst the sequence between $x = 0$ and the point at which elevated ribosomal density curve (r_{pos}/r_{prec30}) drops back to $y = 1$ versus PARS ratios in surrounding plotted sites. Such a difference, however, is seen only in the 2-charge plot (this Figure, part B; Mann Whitney U-test P values, left to right: 0.17, 0.0006, 0.24, 0.08, 0.60). If we instead assume that downstream structure has a pausing effect observable more upstream, a more appropriate test is to compare the PARS ratios from $-30 \geq x < 0$ to those from $0 \geq x \leq 30$. In this case we observe no significant difference in relative propensity for double-strandedness before or after positive charges apart from in the case of a single positive charge alone (this Figure, part B; Mann Whitney U-test, left to right: 0.004, 0.07, 0.12, 0.08 [with the mean $PARS_{pos}/PARS_{prec30}$ decreasing on average after the start of the cluster], 0.60). We note that this version of the test is exceedingly conservative as PARS values had to be antilogged before informative ratios could be calculated. This means that previously negative values (indicating single-strandedness) will now be sandwiched in between 0 and 1, while formerly

positive values (indicating double-strandedness) now span a range of values above one. Hence normalizing the PARS score at a given position by the average PARS value of the preceding 30 codons will exaggerate not only the importance of structured versus free-form RNA, but will also exaggerate small differences in the magnitude of PARS values already denoting double-strandedness. **C.** An alternative calculation showing that RNA structure does not account for the pausing observed near positive charges. Note this Figure does not show the change in PARS values relative to the preceding sequence (as in B), but the average magnitude of the PARS value in that position across aligned transcripts. An average of PARS values plotted above zero indicates a greater likelihood of double-stranded structure in that position on average, while a mean value less than one indicates a propensity for single-strandedness. We find no correlation between the average PARS values after the first charge in the cluster ($0 \geq x \leq 30$) and relative changes in ribosomal density (this Figure, part C; Spearman P, left to right: 0.77, 0.95, 0.87, 0.34, 0.09), as we might have observed if RNA structure has a local effect on ribosomal slowing. Likewise, if structure causes local slowing, we should see a significant difference in the average PARS values between $x = 0$ and the point at which elevated ribosomal density curve (r_{pos}/r_{prec30}) drops back to $y = 1$ versus PARS values in surrounding plotted sites. We do not however detect such a difference (this Figure, part C; Mann Whitney U-test P values, left to right: 0.66, 0.17, 0.30, 0.27, 0.90). Examining whether downstream structure has a pausing effect observable further upstream, we then compare the PARS ratios from $-30 \geq x < 0$ to those from $0 \geq x \leq 30$. In this case we observe no significant difference in relative propensity for double-strandedness before or after positive charges (this Figure, part C; Mann Whitney U-test, left to right: 0.98, 0.98, 0.97, 0.27, 0.90).
