# Supplementary material for: Positively Charged Residues Are the Major Determinants of Ribosomal Velocity
Source: PLoS Biol. 2013 Mar 12;11(3):e1001508. doi: 10.1371/journal.pbio.1001508 (PMC3595205; doi:10.1371/journal.pbio.1001508)
Supplement: Figure S15 — Genes with either high or low footprint coverage both produce consistent slowing patterns after positive charge clusters. To ensure that noise in the location of footprints among genes with fewer overall footprints is not an issue for analysis, we redrew our rpos/rprec30 plots surrounding positive charge clusters using both the bottom half and top half of all genes according to their footprint saturation. Note that in this analysis we do not normalize the footprint counts per codon per gene by mRNA levels. This is because we are not interested in footprint coverage per transcript (as we might be if considering rates or mechanistic issues), but in the statistical power that the total footprint coverage per gene gives us, regardless of the number of transcripts that the footprints were captured from. Areas under the curve were measured as in the main text (see Figure 1). In each case we find similar results to those presented in the main analysis (Figure 5), namely that positive charges additively slow ribosomes. (A) Bottom half of genes: Regression of area under curve∼cluster size, slope = 4.8, r2 = 0.79, p = 0.027. (B) Top half of genes: Regression of area under curve∼cluster size, slope = 0.96, r2 = 0.74, p = 0.039. (PDF) [file pbio.1001508.s015.pdf]

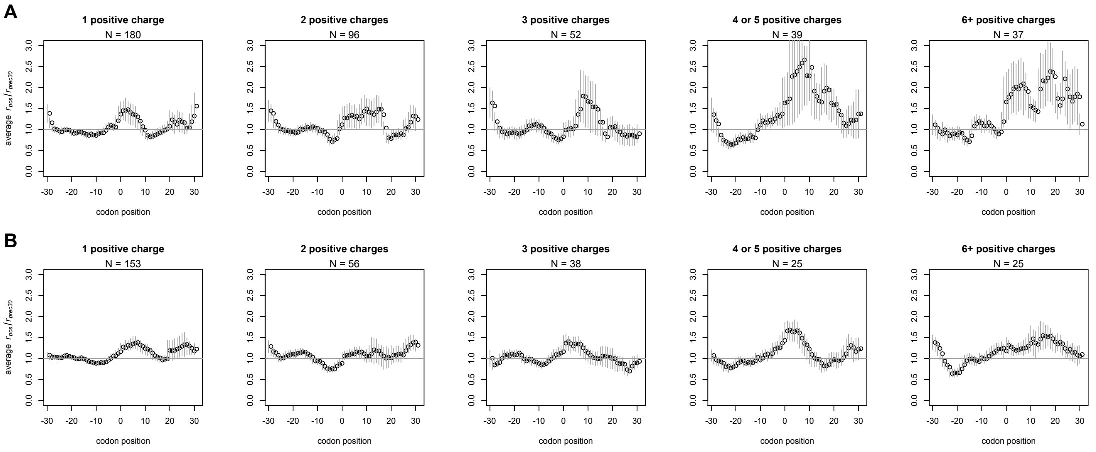

Figure S15. Genes with either high or low footprint coverage both produce consistent slowing patterns after positive charge clusters. To ensure that noise in the location of footprints among genes with fewer overall footprints is not an issue for analysis, we redrew our r_{pos}/r_{prec30} plots surrounding positive charge clusters using both the bottom half and top half of all genes according to their footprint saturation. Note that in this analysis we do not normalize the footprint counts per codon per gene by mRNA levels. This is because we are not interested in footprint coverage per transcript (as we might be if considering rates or mechanistic issues), but in the statistical power that the total footprint coverage per gene gives us, regardless of the number of transcripts that the footprints were captured from. Areas under the curve were measured as in the main text (see Figure 1). In each case we find similar results to those presented in the main analysis (Figure 5), namely that positive charges additively slow ribosomes. **A. Bottom half of genes**: Regression of *area under curve ~ cluster size*, slope = 4.8, r^2 = 0.79, P = 0.027. **B. Top half of genes**: Regression of *area under curve ~ cluster size*, slope = 0.96, r^2 = 0.74, P = 0.039.
