# Supplementary material for: Positively Charged Residues Are the Major Determinants of Ribosomal Velocity
Source: PLoS Biol. 2013 Mar 12;11(3):e1001508. doi: 10.1371/journal.pbio.1001508 (PMC3595205; doi:10.1371/journal.pbio.1001508)
Supplement: Figure S16 — Figure 5 redone on the nonredundant footprint set. We wanted to confirm that the exclusion of footprints that map to two or more potential locations in the genome was not systematically biasing our estimates of ribosomal density. For this reason we replotted the average relative change in ribosomal density within a gene upon translation of encoded positive charge clusters using our nonredundant footprint set (see the end of the Methods section), in effect only considering those locations in the genome to which footprints uniquely map. Considering solely these regions in the transcriptome to which footprints can only ever be mapped unambiguously still shows positive charges additively slow translation. (PDF) [file pbio.1001508.s016.pdf]

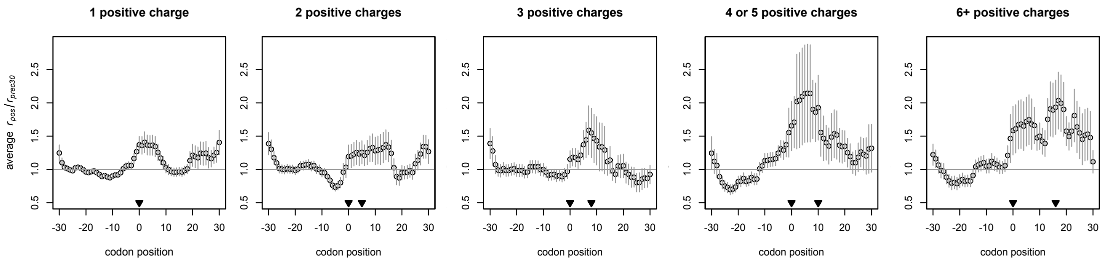

Figure S16. Figure 5 redone on the non-redundant footprint set. We wanted to confirm that the exclusion of footprints which map to two or more potential locations in the genome was not systematically biasing our estimates of ribosomal density. For this reason we replotted the average relative change in ribosomal density within a gene upon translation of encoded positive charge clusters using our non-redundant footprint set (see the end of the Methods section), in effect only considering those locations in the genome to which footprints uniquely map. Considering solely these regions in the transcriptome to which footprints can only ever be mapped unambiguously still shows positive charges additively slow translation.
