# Supplementary material for: Positively Charged Residues Are the Major Determinants of Ribosomal Velocity
Source: PLoS Biol. 2013 Mar 12;11(3):e1001508. doi: 10.1371/journal.pbio.1001508 (PMC3595205; doi:10.1371/journal.pbio.1001508)
Supplement: Note S1 — Codon usage and translation rates: how can codon usage not predict ribosome occupancy but be commonly assumed to be associated with faster translation? (PDF) [file pbio.1001508.s018.pdf]

Note S1
Charneski and Hurst 2013

Codon usage and translation rates: how can codon usage not predict ribosome occupancy but be commonly assumed to be associated with faster translation?

It is has commonly been presumed that a codon specifying a rare tRNA might stall ribosomes. Indeed in some instances, it is simply presumed that codon usage bias must affect ribosome velocity. For example, Higgs and Ran [1] assert that "it is differences in rates [of translation] between alternative cognate codons that are relevant for codon bias. The fact that codon bias occurs in a large number of bacterial genomes means that these rates must indeed differ". While this assertion discounts the possibility that translational accuracy might be important, nonetheless, we can ask how it is that there exists apparent evidence for codon usage altering translation rates but at the same time we see no evidence that codon usage (assayed either with respect to tRNA abundance or with respect to codon usage in the genome as a whole), predicts ribosome occupancy.

There are, we suggest two classes of explanation. First, we note that much evidence is indirect and/or fails to address alternative explanations. Second, and perhaps more interestingly, we argue that it is possible that under normal conditions codon usage should not predict translation rates, but out of normal (equilibrium) conditions codon bias may have a profound effect. This we suggest can explain apparently contradictory evidence.

1. Do experimental results support the view that codon usage modulates translational velocity: problems with predictions and covariates

One possibility is that the codon usage – translation rate assumption is so profoundly held that evidence for the effect is over-interpreted. Indeed, some studies cited as support that codon usage can influence translation rates offer only circumstantial evidence. In such studies the results are possibly consistent with a proposed role for codon usage in modulating ribosomal velocity, but this is not explicitly shown. For example Konigsberg and Godson [2] showed differential codon usage between *dnaG* and a handful of other *Escherichia coli* regulatory genes in comparison with 25 non-regulatory genes and speculated that codon usage may cause differential expression levels of these categories of protein. Another study [3] searched for codon pairs which were overrepresented in lowly expressed and highly expressed genes and denoted them hypothetical attenuating and non-attenuating pairs, respectively, but the authors were unable to use experimental data to further investigate whether such codon pairs might in fact have any effect on ribosomal speed.

Similarly, quite a few experimental studies presuming to show influence of codon bias on gene expression assume that a lack or reduction in protein product reflects slower translation rates [4-7]. However, a heterologous gene transfected into an organism with a different codon usage bias, or a gene engineered to code for the same protein product as the original but with less optimal codons might have unstable mRNA [8-10] or protein products [11]. For instance Coleman et al. [12] claim that substitutions of underrepresented codon pairs into poliovirus coding sequence cause decreased translation rates when such mRNAs are expressed in HeLa cells. Slowing

of translation, however, is not explicitly shown and it is possible that the assays—reduced infectivity or reduced enzymatic activity—could be the result of e.g. structural errors in the proteins which are unrelated to translation speed.

Perhaps more crucially, it is far from clear that changing codon usage bias should greatly change protein titres if translation rate mediated by tRNA abundance is the sole force. After a ribosome has finished processing a transcript for a given gene, the chance that the freed up ribosome will then process a transcript from the same gene is low. The major effect of changing translation speed should thus be changes in cell growth rates not necessarily changes in titre of the protein concerned. More exactly, it has been suggested that changing the translation rate of an mRNA is only likely to directly influence the amount of the focal protein produced if that mRNA can capture a majority of all ribosomes in the cell [13]. In other words, faster translation of an mRNA is not likely to affect the resulting amount of the focal protein in that cell if there is no ribosome readily available to immediately start translating another copy of the same mRNA (provided translation initiation features allow prompt re-initiation). This proposition is supported by transgene studies by Kudla et al. [14] who showed no correspondence between codon usage bias of upregulated versions of GFP, differing only at synonymous sites, and protein titre.

This group also revealed the importance of controlling for translation initiation features at the 5' end of a transcript. A number of studies [4,6,10,15] have examined the effect of synonymous mutations including those near the beginning of transcripts where it is known mRNA structure can influence the frequency of translation initiation. For example Irwin et al. [15] focused on the effect of substitutions of codon pairs at the 5' end, assuming that if they were translated slowly it would prevent re-initiation of *lacZ* by another ribosome and hence reduce the amount of beta-galactosidase activity observable. But their test may have rather interfered with mRNA secondary structure important for translation initiation. It should also be noted that Irwin et al. [15] found that *over*-represented rare pairs of codons in E coli could attenuate ribosomes. Their claim however was later disputed by Cheng and Goldman [16] who could not confirm Irwin's findings. In another case, Goldman et al. [4] observed that insertion of 9 consecutive low-usage leucine codons near the 5' end of a transcript blocked translation, but no similar effects were seen when the 9 consecutive codons were introduced further downstream, suggesting their results may be due to interference with 5' transcript folding required for initiation.

Even when the experiments are robust, further problems with covariates (i.e. alternative explanations) abound. For example the slowing (as inferred by reduced expression level) thought to be due to consecutive rare AGG or AGA codons in *E. coli* [e.g.17,18-24] may be due to tandem codons resembling the Shine Dalgarno sequence and interacting with the translating ribosome [25-27] or indeed in some cases the positive charge on the incorporated arginine residue may slow the ribosome [28] (see also this paper). Similarly, Sørensen et al. [7] inferred average translation rates from the time required for *E. coli* to incorporate radioactive methionine into β-galactosidase containing inserts full of either rare or common codons. Their rare-codon insert, however, contained more (and more clustered) codons encoding positive charges, which may account for the slowing of ribosomes during translation of rare codons that they infer.

A perfect test is indeed very hard to envisage as any change to codon usage is likely also to affect many aspects of the processing of the RNA, not least the RNA structure, for which we found good evidence of an effect on translation rates. For instance it has been reported that an approximately 30bp insert rich in GAA (Glu) codons is translated ~3.4 times faster than a similar-sized insert composed of GAG (Glu) codons [29]. These two inserts must have profound effects on RNA stability, most especially GAG inserts as runs of GAG are likely to form strong stem structures.

While claims that changing the codon usage modulates levels of that protein of the modified gene because of changes in translation rate owing to tRNA availability should be treated with considerable caution, some more direct reports lack robust statistics. As an example, Varenne et al. [30] report that translation rate along mRNA varies with tRNA availability at different codons (although some of the slowing they observe they say cannot be attributed to differential codon usage). They compared the distribution of electrophoretic intermediates to that predicted by assuming that tRNA concentration is the rate-limiting factor in ribosomal translocation, with the aim of investigating how well the prediction matched the observed. However, there are a few problems with the approach. 'Analogous peaks' between the observed and predicted were detected not by a stringent methodology but by attempting to locate matching peaks between noisy curves by eye. Nor was it determined if detected slowing along the ribosome was significant. Additionally although a good correlation between the observed and predicted curves was claimed, a statistical test of a correlation, or any type of statistical test to establish similarity between the two curves, was not performed. We do not wish to assert that the conclusions of Varenne et al. are incorrect, just that they lack normal statistical support.

2. Normal and abnormal conditions and the balance model of codon usage

Above we have suggested that the tendency to suppose a direct link, mediated by codon usage and tRNA abundances, has often led to alternative interpretations not being considered. While the problem of alternative explanations must always be an issue, we don't wish to suppose that there is no evidence that changes in codon usage bias cannot *sometimes* affect translation rates. We note, however, that the best of the evidence finds support for an effect under abnormal conditions. For example, rare Arg codons (AGG) can limit protein synthesis in *E. coli* compared to the same amino acid sequence comprised of non-rare codons (CGT) [20]. This effect, however, was only observed under extreme conditions involving multiple consecutive rare codons and transcription at very high levels. Such runs of consecutive rare codons in highly expressed transcripts are unlikely to be observed in endogenous genes.

Similarly, Pedersen [31] found a less than two-fold difference in the translation rate between rare and common codons upon comparing the speed of translation of ribosomal proteins, but only when they were expressed in high-copy plasmids with an up-promoter mutation, presumably increasing drain on the tRNA pool. Similarly Misra and Reeves [23] reported stalling (as inferred by accumulation of an intermediate peptide) at a rare Arg codon which could be rescued by providing $tRNA_{Arg}$(AGA), but this stalling effect was observed upon transcribing the gene from a multicopy plasmid and may not reflect *in vivo* conditions. Komar et al. [32] showed that substitution of 16 rare for frequent synonymous codons in a 21-codon stretch

resulted in loss of a protein intermediate as visualized by gel electrophoresis, but the cloned transgene was expressed from the high-expression viral T7 promoter. Kudla et al.'s [14] demonstration that codon usage bias predicts growth rates is also consistent with an effect on translation rates for grossly upregulated genes.

It is the extreme abnormality of the conditions needed to show an effect that we think may underpin a correspondence between these experimental results and our results. Let us suppose then that we can show that codon usage of a highly upregulated gene affects the translation rate. How can this observation square with the absence of higher ribosome occupancy with transcripts under normal conditions in domains rich in rare codons?

Let us consider again the balance model proposed by Qian et al. [33]. They note that if highly expressed genes use codons corresponding to the most abundant tRNAs then it doesn't follow that they will be translated any faster than rare transcripts using rare codons. The key parameter is not the absolute tRNA abundance but the tRNA availability. If highly abundant transcripts all require the same tRNA, then this acts as a drain on the availability of that tRNA. The waiting time for a ribosome to find a rare but little in demand tRNA may then be the same (or approximately so) as the waiting time to find a "common" but much in demand tRNA. In one case the pool is small and the demand low (rare codons in lowly expressed transcripts) in the other the tRNA pool is large but the demand also large (a common codon in an abundant transcript). We can then imagine an equilibrium situation in which the ribosome waiting time is the same for all codons as the demand and supply of each are balanced. This is consistent with our observation that, under normal growth conditions, codon usage doesn't predict ribosome occupancy. However, the same model can predict that under abnormal conditions, we might see an effect as the situation has been forced far out of equilibrium. Over-express a transcript rich in rarely used codons and the ribosome should now slow as the demand for the rare tRNAs exceeds supply. Likewise, we expect that gross modification of tRNA pools should have gross effects on translational speed as the system has been shifted away from the demand-supply equilibrium.

This buffering of translation speed (by a correspondence between global codon usage and tRNA pools in the above scenario) is also recapitulated on an enzyme catalytic level when considering the translation of even a single codon. Curran and Yarus [34] published experimental findings that the rates for charged tRNA selection at different codons span a 25-fold range. If codon usage is indeed selected for some codons to be translated quickly and others slowly, then we should expect that codons with intrinsically, mechanistically fast rates of aminoacyl-tRNA selection are enlisted for the former and codons with intrinsically slow rates of aminoacyl-tRNA selection are recruited for the latter. The authors however go on to make the following observation: that most codons whose aminoacyl-tRNAs are selected either intrinsically rapidly or slowly by the ribosome have either low or high tRNA concentrations within the cell, respectively. This would suggest that intrinsic differences in the translation speeds of certain codons are not exploited but rather normalized via their supply lines.

In evolutionary terms we expect a move out of equilibrium might occur whenever selection operates on growth rates. Imagine a slow-growing organism comes under selection to grow faster. Under this circumstance the translational apparatus will be

under selection to work faster (more ribosomes, more tRNAs). But in addition, the more abundant transcripts will come under selection to shift codon usage towards the most abundant tRNAs (whichever they may be) to free up ribosomes. These features have been observed [1,35]. Once the systems returns to a supply-demand equilibrium, however, there is no reason to suppose that rare codons in lowly expressed transcripts will be processed any slower than common codons in abundant transcripts. Thus the supply-demand balance model is consistent both with our observations and with the finding that codon bias is higher in faster growing organisms that also have more tRNAs.

Supplemental References

1. Higgs PG, Ran W (2008) Coevolution of codon usage and tRNA genes leads to alternative stable states of biased codon usage. Mol Biol Evol 25: 2279-2291.
2. Konigsberg W, Godson GN (1983) Evidence for use of rare codons in the dnaG gene and other regulatory genes of Escherichia coli. Proc Natl Acad Sci U S A 80: 687-691.
3. Boycheva S, Chkodrov G, Ivanov I (2003) Codon pairs in the genome of Escherichia coli. Bioinformatics 19: 987-998.
4. Goldman E, Rosenberg AH, Zubay G, Studier FW (1995) Consecutive low-usage leucine codons block translation only when near the 5' end of a message in Escherichia coli. J Mol Biol 245: 467-473.
5. Nakamura M, Sugiura M (2011) Translation efficiencies of synonymous codons for arginine differ dramatically and are not correlated with codon usage in chloroplasts. Gene 472: 50-54.
6. Cannarozzi G, Schraudolph NN, Faty M, von Rohr P, Friberg MT, et al. (2010) A role for codon order in translation dynamics. Cell 141: 355-367.
7. Sørensen MA, Kurland CG, Pedersen S (1989) Codon usage determines translation rate in Escherichia coli. J Mol Biol 207: 365-377.
8. Stanssens P, Remaut E, Fiers W (1986) Inefficient translation initiation causes premature transcription termination in the lacZ gene. Cell 44: 711-718.
9. Petersen C (1987) The functional stability of the lacZ transcript is sensitive towards sequence alterations immediately downstream of the ribosome binding site. Mol Gen Genet 209: 179-187.
10. Hoekema A, Kastelein RA, Vasser M, de Boer HA (1987) Codon replacement in the PGK1 gene of Saccharomyces cerevisiae: experimental approach to study the role of biased codon usage in gene expression. Mol Cell Biol 7: 2914-2924.
11. Kurland CG (1991) Codon bias and gene expression. FEBS Lett 285: 165-169.
12. Coleman JR, Papamichail D, Skiena S, Futcher B, Wimmer E, et al. (2008) Virus attenuation by genome-scale changes in codon pair bias. Science 320: 1784-1787.
13. Andersson SG, Kurland CG (1990) Codon preferences in free-living microorganisms. Microbiol Rev 54: 198-210.
14. Kudla G, Murray AW, Tollervey D, Plotkin JB (2009) Coding-Sequence Determinants of Gene Expression in Escherichia coli. Science 324: 255-258.

15. Irwin B, Heck JD, Hatfield GW (1995) Codon pair utilization biases influence translational elongation step times. J Biol Chem 270: 22801-22806.
16. Cheng L, Goldman E (2001) Absence of effect of varying Thr-Leu codon pairs on protein synthesis in a T7 system. Biochemistry 40: 6102-6106.
17. Hu X, Shi Q, Yang T, Jackowski G (1996) Specific replacement of consecutive AGG codons results in high-level expression of human cardiac troponin T in Escherichia coli. Protein Expr Purif 7: 289-293.
18. Bonekamp F, Jensen KF (1988) The AGG codon is translated slowly in E. coli even at very low expression levels. Nucleic Acids Res 16: 3013-3024.
19. Rosenberg AH, Goldman E, Dunn JJ, Studier FW, Zubay G (1993) Effects of consecutive AGG codons on translation in Escherichia coli, demonstrated with a versatile codon test system. J Bacteriol 175: 716-722.
20. Robinson M, Lilley R, Little S, Emtage JS, Yarranton G, et al. (1984) Codon usage can affect efficiency of translation of genes in Escherichia coli. Nucleic Acids Res 12: 6663-6671.
21. Wang BQ, Lei L, Burton ZF (1994) Importance of codon preference for production of human RAP74 and reconstitution of the RAP30/74 complex. Protein Expr Purif 5: 476-485.
22. Spanjaard RA, Chen K, Walker JR, van Duin J (1990) Frameshift suppression at tandem AGA and AGG codons by cloned tRNA genes: assigning a codon to argU tRNA and T4 tRNA(Arg). Nucleic Acids Res 18: 5031-5036.
23. Misra R, Reeves P (1985) Intermediates in the synthesis of TolC protein include an incomplete peptide stalled at a rare Arg codon. Eur J Biochem 152: 151-155.
24. Varenne S, Lazdunski C (1986) Effect of distribution of unfavourable codons on the maximum rate of gene expression by an heterologous organism. J Theor Biol 120: 99-110.
25. Wen JD, Lancaster L, Hodges C, Zeri AC, Yoshimura SH, et al. (2008) Following translation by single ribosomes one codon at a time. Nature 452: 598-603.
26. Li GW, Oh E, Weissman JS (2012) The anti-Shine-Dalgarno sequence drives translational pausing and codon choice in bacteria. Nature 484: 538-541.
27. Ivanov I, Alexandrova R, Dragulev B, Saraffova A, AbouHaidar MG (1992) Effect of tandemly repeated AGG triplets on the translation of CAT-mRNA in E. coli. FEBS Lett 307: 173-176.
28. Lu J, Deutsch C (2008) Electrostatics in the ribosomal tunnel modulate chain elongation rates. J Mol Biol 384: 73-86.
29. Sørensen MA, Pedersen S (1991) Absolute in vivo translation rates of individual codons in Escherichia coli. The two glutamic acid codons GAA and GAG are translated with a threefold difference in rate. J Mol Biol 222: 265-280.
30. Varenne S, Buc J, Lloubes R, Lazdunski C (1984) Translation is a non-uniform process. Effect of tRNA availability on the rate of elongation of nascent polypeptide chains. J Mol Biol 180: 549-576.
31. Pedersen S (1984) Escherichia coli ribosomes translate in vivo with variable rate. EMBO J 3: 2895-2898.
32. Komar AA, Lesnik T, Reiss C (1999) Synonymous codon substitutions affect ribosome traffic and protein folding during in vitro translation. FEBS Lett 462: 387-391.
33. Qian W, Yang J-R, Pearson NM, Maclean C, Zhang J (2012) Balanced Codon Usage Optimizes Eukaryotic Translational Efficiency. PLoS Genet 8: e1002603.

34. Curran JF, Yarus M (1989) Rates of aminoacyl-tRNA selection at 29 sense codons in vivo. J Mol Biol 209: 65-77.
35. Rocha EPC (2004) Codon usage bias from tRNA's point of view: Redundancy, specialization, and efficient decoding for translation optimization. Genome Research 14: 2279-2286.
