# Supplementary material for: Positively Charged Residues Are the Major Determinants of Ribosomal Velocity
Source: PLoS Biol. 2013 Mar 12;11(3):e1001508. doi: 10.1371/journal.pbio.1001508 (PMC3595205; doi:10.1371/journal.pbio.1001508)
Supplement: Note S2 — Only positive charge is capable of explaining the region of strongest translational pausing within transcripts. (PDF) [file pbio.1001508.s019.pdf]

Note S2

Only positive charge is capable of explaining the region of strongest translational pausing within transcripts

Having identified, within a given transcript, the two 10-codon windows with the largest difference in ribosomal densities, we then determined how often the denser region was more associated with each potentially slowing feature—positive charge, less optimal codons, pairs of rare codons, pairs of rare 6-mers, or a window of mRNA double-stranded structure immediately downstream (see Methods). If positive charge can indeed explain the greatest ribosomal deceleration within that transcript then across mRNAs we should expect that, even though subject to some stochasticity in ribosomal flow, the difference in the number of positive charges between the two windows (number of charges in high occupancy window-number of charges in low occupancy window) positively correlate with the difference in average ribosomal occupancy between the two windows (ribosomal occupancy in high occupancy window-ribosomal occupancy in low occupancy window) such that the excess magnitude of positive charge pairs with an excess magnitude of ribosomal density. Similarly if rare pairs are responsible for slowing, we should observe that the excess magnitude of rare pairs positively correlates with an excess magnitude of ribosomal density across transcripts, implying the more occluded window tends to contain more rare pairs. The same is true for rare 6-mers. However if basal codon optimality is responsible for the extremes in ribosomal occupancy between the two intra-transcript windows then we should expect a negative correlation between the difference in tAI between the two windows, meaning that across transcripts the window with the lower tAI tends to also be the window with more ribosomal footprints.

Of all the putative slowing features we consider, only charge is more often than not associated with the higher occupancy window within each transcript. Comparing each pair of intra-transcript windows across genes, we find that an excess of ribosomal density indeed correlates with an excess of positive charges as expected (Spearman rho 0.08, P = 6.4e-09). We are unable to detect a correlation between an excess of ribosomal density and an excess of rare codon pairs (Spearman P = 0.16), while that between the difference in density and an excess of rare 6-mers, while significant, is slight (Spearman rho = -0.04, P = 0.0066) and goes in the opposite direction expected were rare 6-mers capable of explaining slowing (considering only those genes which have at least one rare pair or rare 6-mer in either window, respectively). The correlation between difference in ribosomal density with difference in tAI between the two windows also goes in the wrong direction to explain pausing (Spearman rho 0.05, P = 0.00056), i.e. more occluded windows in fact tend to have higher (more optimal) tAIs. We do detect a negative correlation between the difference in the number of rare pairs between the two windows and the tAI of the rare pair (defined as the geometric mean of the tAIs of the two individual codons) (Spearman rho -0.28, P = 1.711e-05), implying that the pairs of rare codons in the higher occupancy window do indeed tend to be "less optimal". Nevertheless, the fact that the difference in number of a specific rare pair between windows and the corresponding mean difference in ribosomal density for windows containing that pair type negatively correlate (Spearman rho -0.15, P = 0.027) illustrates that more rare pairs, even if low in tAI, still more commonly associate with faster-travelling ribosomes.
