# Supplementary material for: Positively Charged Residues Are the Major Determinants of Ribosomal Velocity
Source: PLoS Biol. 2013 Mar 12;11(3):e1001508. doi: 10.1371/journal.pbio.1001508 (PMC3595205; doi:10.1371/journal.pbio.1001508)
Supplement: Note S3 — Trend of slowing increasing with charge is not random. (PDF) [file pbio.1001508.s020.pdf]

Note S3

Trend of slowing increasing with charge is not random

To ask whether the trend we show in Figure 3C might result randomly given the specific genes we have used and their corresponding footprints, we performed the following test. We started with lists of all genes used in each positive charge plot in Figure 5. In the case of for example the 1-positive charge clusters, we counted the total number of identified 1-positive charge segments which went into making the 1-positive charge plot in Figure 5. For each iteration we identified the same number of segments (each time from a random gene in that list, and from a random location within that gene) and for each 'pseudo one-positive charge segment' we calculated r_{pos}/r_{prec30}; then after each iteration (out of 1000 total) we calculated the mean r_{pos}/r_{prec30} for that iteration. We then performed the similar randomizations for the other positive charge cluster sizes separately.

We partitioned the r_{pos}/r_{prec30} results at random into 1000 sets for which area under the curve plots can be calculated. For each set, we calculated the regression of $y{\sim}x$, where y is the vector of the area under the curves calculated from the randomization results (in exactly the same way as done in the main text, see Methods and Figure 1), and x is the vector of the average number of positive charges in each cluster used in the original analysis (1, 2, 3, 4.328, 6.823). Our randomization P value is then calculated as P = $(m+1)/(n+1)$, where m is the number of randomized sets for which the regression P value is significant and the slope is greater than or equal to that observed, and n is the sample size (1000). We find that the chance of detecting the trend we show in Figure 3C at random from just those genes used to make the Figure is indeed low (P = 0.011).
