# Supplementary material for: Positively Charged Residues Are the Major Determinants of Ribosomal Velocity
Source: PLoS Biol. 2013 Mar 12;11(3):e1001508. doi: 10.1371/journal.pbio.1001508 (PMC3595205; doi:10.1371/journal.pbio.1001508)
Supplement: Table S1 — Table 1 of the main text redone using rare codons that are defined to occur with genomic infrequency shows rare codons do not slow ribosomes. In the main text, we investigate whether nonoptimal codons—that is, those with low tAI scores—might slow codons and find that they do not. To ensure that our finding that these “rare” codons do not slow ribosomes does not simply hinge on our definition of “rare,” we have repeated the analysis using an alternative definition. Here, we define “rare” codons according to their actual frequency in the genome as measured from our set of filtered genes. This rare set, of equal size to the rare tAI set, comprises the following codons: CGG, CGC, CGA, TGC, CCG, CTC, GGG, GCG, CGT, CCC, CAC, TGT, ACG, TCG, and AGG. Quantiles of the difference in average ribosomal density between the two windows identified within a transcript are shown, with q1 representing the smallest differences and q4 the largest. A score of 1 indicates the putative retarding feature is more present within the more occluded intra-transcript window; −1, less present; 0, present in both windows in equal amounts. Rare (infrequent) codons and codon pairs tend to be found more in the less dense (faster translated) window. Similarly, the presence of rare pairs and rare codons decreases in the slowly translated windows as the difference in degree of ribosomal slowing grows. (PDF) [file pbio.1001508.s021.pdf]

Table S1.

		$q1_{\Delta r}$ (count)	$q2_{\Delta r}$	$q3_{\Delta r}$	$q4_{\Delta r}$	χ^2 test P value (Bonferroni correction)
**A. rare codons score**	**1**	**354**	**345**	**338**	**232**	**8.7e-07 (2.6e-06)**
	**0**	**409**	**397**	**398**	**494**	**0.0015 (0.0045)**
	**-1**	**483**	**503**	**509**	**519**	**0.71**
	**Binomial test on +1 and -1 charge score counts, P value (Bonferroni correction)**	**9.3e-06 (3.7e-05)**	**6.4e-08 (2.6e-07)**	**4.6e-09 (1.8e-08)**	**<2.2e-16 (8.8e-16)**	**-**
**B. rare pair score**	**1**	**60**	**73**	**49**	**25**	**2.5e-05 (7.5e-09)**
	**0**	**1074**	**1057**	**1084**	**1148**	**0.23**
	**-1**	**112**	**115**	**112**	**72**	**0.0063 (0.019)**
	**Binomial test on +1 and -1 tAI score counts, P value (Bonferroni correction)**	**9.0e-05 (0.00036)**	**0.0027 (0.011)**	**7.5e-07 (3.0e-06)**	**1.9e-06 (7.7e-06)**	**-**

Table S1. Table 1 of the main text redone using rare codons which are defined to occur with genomic infrequency shows rare codons do not slow ribosomes. In the main text we investigate whether non-optimal codons, i.e. those with low tAI scores, might slow codons and find that they do not. To ensure that our finding that these 'rare' codons do not slow ribosomes does not simply hinge on our definition of 'rare', we have repeated the analysis using an alternative definition. Here, we define 'rare' codons according to their actual frequency in the genome as measured from our set of filtered genes. This rare set, of equal size to the rare tAI set, comprises the following codons: CGG, CGC, CGA, TGC, CCG, CTC, GGG, GCG, CGT, CCC, CAC, TGT, ACG, TCG, AGG. Quantiles of the difference in average ribosomal density between the two windows identified within a transcript are shown, with q1 representing the smallest differences and q4 the largest. A score of 1 indicates the putative retarding feature is more present within the more occluded intra-transcript window; -1, less present; 0, present in both windows in equal amounts. Rare (infrequent) codons and codon pairs tend to be found more in the less dense (faster translated) window. Similarly, the presence of rare pairs and rare codons decreases in the slowly-translated windows as the difference in degree of ribosomal slowing grows.
