# Supplementary material for: Positively Charged Residues Are the Major Determinants of Ribosomal Velocity
Source: PLoS Biol. 2013 Mar 12;11(3):e1001508. doi: 10.1371/journal.pbio.1001508 (PMC3595205; doi:10.1371/journal.pbio.1001508)
Supplement: Table S2 — Genes with identified rare codon clusters are not disproportionately sampled from lowly expressed genes. Could it be that large changes in ribosomal occupancy are not observed after rare clusters (Figure 2A and Figure 3A) because the clusters we identify are more likely to come from lowly expressed genes—that is, genes that do not have high translation levels and for which it may be less likely that ribosomal footprints will be sampled? We used the average footprint count of a gene (total number of footprints within the coding sequence divided by gene length) as a proxy for protein expression levels. If anything, there are more genes with nonoptimal codon clusters from genes that have more footprint reads (χ2, p<2.2e-16) so we do not consider this an issue. (PDF) [file pbio.1001508.s022.pdf]

Table S2.

**Protein expression quantiles**	**q1** (least expressed)	**q2**	**q3**	**q4** (most expressed)
**Number of genes with identified rare codon cluster**	90	67	99	345

Table S2. **Genes with identified rare codon clusters are not disproportionately sampled from lowly expressed genes.** Could it be that large changes in ribosomal occupancy are not observed after rare clusters (Fig. 2A, Fig. 3A) because the clusters we identify are more likely to come from lowly expressed genes, i.e. genes which do not have high translation levels and for which it may be less likely that ribosomal footprints will be sampled? We used the average footprint count of a gene (total number of footprints within the coding sequence divided by gene length) as a proxy for protein expression levels. If anything, there are more genes with non-optimal codon clusters from genes which have more footprint reads (χ^2, $P < 2.2e-16$) so we do not consider this an issue.
