# Supplementary material for: Positively Charged Residues Are the Major Determinants of Ribosomal Velocity
Source: PLoS Biol. 2013 Mar 12;11(3):e1001508. doi: 10.1371/journal.pbio.1001508 (PMC3595205; doi:10.1371/journal.pbio.1001508)
Supplement: Table S3 — Sequence similarity to the yeast Kozak sequence cannot explain the greatest slowing within transcripts. Given that transcript similarity to the Shine-Dalgarno sequence has been shown to slow ribosomes in bacteria due to interactions of the sequence with components of the ribosomal RNA [17], we wondered whether translation speed in yeast might not be modulated by codon usage per se but by the ability of ribosomes to bind to transcript sequence that mirrors the eukaryotic Kozak sequence. Specifically, we wanted to determine whether codons that are in high-ribosomal occupancy windows within a gene might be more likely to correspond to the Kozak sequence (as compared to codons in low-occupancy windows within the same genes) and hence bind ribosomes, slowing translation. We first determined which codons were enriched in the Kozak sequence relative to the codon frequencies seen throughout the yeast genome at large using a simple randomization. Nucleotide frequencies at each position of the Kozak sequence in yeast were taken from Cavener and Ray 1991 [57]. To determine the frequencies of all the possible “codons” among the Kozak sequence space, we randomly created 20,000 possible Kozak sequences from the delineated nucleotide frequencies at each site in the consensus sequence. We then counted all possible triplet “codons” within each sequence, regardless of reading frame (since we assume that as the ribosome traverses RNA, it may bind the Kozak sequence regardless of the surrounding reading frame). The counts of all possible RNA triplets that we observe within our simulated sequences are the observed “codons” within the Kozak sequence. In order to determine whether or not certain codons are over- or underused in the Kozak sequence, we compare them to the counts of codons observed (again in any reading frame) across 20,000 randomized sequences derived from the basal codon frequencies in the S. cerevisiae genome and of the same length as the Kozak sequence. We calculate Z, a [file pbio.1001508.s023.pdf]

Table S3.

		$q1_{\Delta r}$ (count)	$q2_{\Delta r}$	$q3_{\Delta r}$	$q4_{\Delta r}$	χ^2 test P value (Bonferroni correction)
**A.** ***Z*** **score**	**1**	**483**	**517**	**479**	**522**	**0.39**
	**0**	**337**	**334**	**340**	**347**	**0.96**
	**-1**	**426**	**394**	**426**	**376**	**0.21**
	**Binomial test on +1 and -1 charge score counts, P value (Bonferroni correction)**	**0.063**	**5.2e-05 (0.00021)**	**0.084**	**1.2e-06 (5.0e-06)**	**-**
**B.** ***Z*** **score when charge score = 0**	**1**	**94**	**99**	**84**	**87**	**0.68**
	**0**	**71**	**59**	**70**	**57**	**0.48**
	**-1**	**91**	**91**	**88**	**63**	**0.084**
	**Binomial test on +1 and -1 tAI score counts, P value (Bonferroni correction)**	**0.88**	**0.61**	**0.82**	**0.060**	**-**
**C.** ***Z*** **score adjusted for amino acid usage**	**1**	**323**<br>*358*	**340**<br>*340*	**314**<br>*276*	**251**<br>*265*	**0.0020 (0.0060)**<br>*0.00013 (0.00039)*
	**0**	**373**<br>*412*	**393**<br>*424*	**381**<br>*435*	**382**<br>*436*	**0.91**<br>*0.83*
	**-1**	**330**<br>*256*	**285**<br>*254*	**283**<br>*267*	**267**<br>*199*	**0.056**<br>*0.0095 (0.028)*
	**Binomial test on +1 and -1 rare pair score counts, P value (Bonferroni correction)**	**0.81**<br>*4.4e-05 (0.00018)*	**0.031 (0.12)**<br>*0.00048 (0.0019)*	**0.22**<br>*0.73*	**0.51**<br>*0.0025 (0.010)*	**-**

Table S3. Sequence similarity to the yeast Kozak sequence cannot explain the greatest slowing within transcripts. Given that transcript similarity to the Shine-Dalgarno sequence has been shown to slow ribosomes in bacteria due to interactions of the sequence with components of the ribosomal RNA [17], we wondered whether translation speed in yeast might not be modulated by codon usage per se but by the ability of ribosomes to bind to transcript sequence which mirrors the eukaryotic Kozak sequence. Specifically, we wanted to determine whether codons which are in high-ribosomal occupancy windows within a gene might be more likely to correspond to the Kozak sequence (as compared to codons in low-occupancy windows within the same genes) and hence bind ribosomes, slowing translation. We first determined which codons were enriched in the Kozak sequence relative to the codon frequencies seen throughout the yeast genome at large using a simple randomization. Nucleotide frequencies at each position of the Kozak sequence in yeast were taken from Cavener

and Ray 1991 [57]. To determine the frequencies of all the possible 'codons' among the Kozak sequence space, we randomly created 20000 possible Kozak sequences from the delineated nucleotide frequencies at each site in the consensus sequence. We then counted all possible triplet 'codons' within each sequence, regardless of reading frame (since we assume that as the ribosome traverses RNA, it may bind the Kozak sequence regardless of the surrounding reading frame). The counts of all possible RNA triplets that we observe within our simulated sequences are the observed 'codons' within the Kozak sequence. In order to determine whether or not certain codons are over- or under-used in the Kozak sequence, we compare them to the counts of codons observed (again in any reading frame) across 20000 randomized sequences derived from the basal codon frequencies in the S. cerevisiae genome and of the same length as the Kozak sequence. We calculate *Z*, a measure of the over- or under-usage of a particular codon within the Kozak sequence (as compared to the rest of the genome) as Z_{codon} = [Observed codon count (in Kozak sequence) – Expected count (from genome frequencies)] / Expected SD of codon. We can then perform a test similar to the one in Methods V, but where we consider possible slowing codons to be those with a positive *Z* (GAT GAC AAC TGC CAA GGC GTA GTC TAT ACA TGG ATA CAT AAA TGT AAT ATG). A score of 1 indicates there are more codons with positive *Z* within the more occluded intra-transcript window; -1, less present; 0, present in both windows in equal amounts. A. Similarity to Kozak sequence cannot explain slowing in several quantiles (binomial tests), nor can it explain increased slowing (χ^2 tests). B. Even when the number of positive charges is the same between the two windows, we do not detect a significant contribution of similarity to Kozak sequence to slowing. C. Controlling for amino acid usage in two different ways, we detect no contribution of similarity to Kozak sequence to slowing; in fact, as the degree of slowing increases, the ability of Kozak similarity to explain slowing decreases (χ^2 tests). Method one (in bold): a gene is scored '1' if the slow window contains more codons with positive *Z*, '-1' if it contains fewer. Method two (in italics): the magnitude of all the positive *Z* values is averaged in each window, and the gene is scored '1' if the slower window has a higher average *Z*, '-1' if its average *Z* is lower.
