# Supplementary material for: Positively Charged Residues Are the Major Determinants of Ribosomal Velocity
Source: PLoS Biol. 2013 Mar 12;11(3):e1001508. doi: 10.1371/journal.pbio.1001508 (PMC3595205; doi:10.1371/journal.pbio.1001508)
Supplement: Table S4 — Table 1 tAI score tests controlled for amino acid content. Could differences in amino acid usage between the two windows be biasing our result that neither codon usage nor rare pairs slow ribosomes (Table 1)? It could be that certain amino acids only have relatively high or low tAIs, and a preponderance of such amino acids in one window over the other could cause an apparent preference for (non-)optimal codons, which is in fact a preference for a certain amino acid. For this reason we tested whether differences in amino acid usage between the high and low ribosomal occupancy windows within a transcript systematically alter the tAI scores (and hence the resulting interpretation of the contribution of codon usage to ribosomal density) in our window comparison analysis. To do this, we identified the same high and low ribosomal occupancy windows within a transcript as above. This time, however, we considered only amino acids that are coded for at least once within each window. Within each intra-transcript window, we identified all codons that code for amino acid x and quantified the contribution of tAI to ribosomal occupancy using two approaches: (Method 1) The average tAI of all the codons coding for amino acid (aa) x was calculated for each window, and that amino acid was assigned an aa-tAIscore of 1, 0, or −1, depending on whether the tAI in the higher ribosomal occupancy window was lower (and hence capable of explaining the increased ribosomal density), the same, or higher than that in the other window, respectively. All of the aa-tAI scores for a given gene were counted independently—in other words, for a given gene, it was possible to calculate more than one aa-tAI score, and all these aa-tAI scores contributed to the final matrix. (Method 2) The average tAI of all the codons coding for amino acid x in each window was calculated, similarly to Method 1, but a tAI score is not yet assigned. Instead, the average tAI is first determined for each amino acid present in [file pbio.1001508.s024.pdf]

Table S4.

		q1$_{\Delta r}$ (count)	q2$_{\Delta r}$	q3$_{\Delta r}$	q4$_{\Delta r}$	χ^2 test P value
**A.**<br>**aatAI score**	**1**	**18**<br>*634*	**20**<br>*649*	**24**<br>*637*	**28**<br>*613*	**0.45**<br>*0.79*
	**0**	**255**<br>*5*	**220**<br>*5*	**238**<br>*2*	**279**<br>*7*	**0.054**<br>*0.57*
	**-1**	**24**<br>*606*	**24**<br>*590*	**15**<br>*606*	**22**<br>*626*	**0.46**<br>*0.78*
	**Binomial test on +1 and -1 charge score counts, P value**	**0.44**<br>*0.44*	**0.65**<br>*0.10*	**0.20**<br>*0.39*	**0.48**<br>*0.73*	**-**
**B.**<br>**aatAI score (charge score = 0, original Δr quantiles)**	**1**	**1**<br>*133*	**3**<br>*129*	**2**<br>*129*	**5**<br>*97*	**0.44**<br>*0.075*
	**0**	**42**<br>*1*	**36**<br>*0*	**31**<br>*0*	**46**<br>*1*	**0.34**<br>*1*
	**-1**	**6**<br>*124*	**4**<br>*129*	**3**<br>*107*	**1**<br>*109*	**0.29**<br>*0.39*
	**Binomial test on +1 and -1 tAI score counts, P value**	**0.125**<br>*0.62*	**1.0**<br>*1.0*	**1.0**<br>*0.17*	**0.22**<br>*0.44*	**-**
**C.**<br>**aatAI score (charge score = 0, recalculated Δr quantiles)**	**1**	**1**<br>*123*	**3**<br>*119*	**2**<br>*137*	**5**<br>*109*	**045**<br>*0.35*
	**0**	**38**<br>*0*	**33**<br>*1*	**33**<br>*0*	**51**<br>*1*	**0.13**<br>*1*
	**-1**	**4**<br>*117*	**3**<br>*119*	**5**<br>*103*	**2**<br>*130*	**0.81**<br>*0.37*
	**Binomial test on +1 and -1 rare pair score counts, P value**	**0.38**<br>*0.75*	**1.0**<br>*1.0*	**0.45**<br>*0.033*	**0.45**<br>*0.20*	**-**

Table S4. Table 1 tAI score tests controlled for amino acid content. Could differences in amino acid usage between the two windows be biasing our result that neither codon usage nor rare pairs slow ribosomes (Table 1)? It could be that certain amino acids only have relatively high or low tAIs, and a preponderance of such amino acids in one window over the other could cause an apparent preference for (non-)optimal codons which is in fact a preference for a certain amino acid. For this reason we tested whether differences in amino acid usage between the high and low ribosomal occupancy windows within a transcript systematically alter the tAI scores (and hence the resulting interpretation of the contribution of codon usage to ribosomal density) in our window comparison analysis. To do this we identified the same high and low ribosomal occupancy windows within a transcript as above. This time, however, we considered only amino acids which are coded for at least once within

each window. Within each intra-transcript window, we identified all codons that code for amino acid *x* and quantified the contribution of tAI to ribosomal occupancy using two approaches: **Method 1)** The average tAI of all the codons coding for amino acid (aa) *x* was calculated for each window, and that amino acid was assigned an aa-tAIscore of 1, 0, or -1, depending on whether the tAI in the higher ribosomal occupancy window was lower (and hence capable of explaining the increased ribosomal density), the same, or higher than that in the other window, respectively. All of the aa-tAI scores for a given gene were counted independently—in other words, for a given gene it was possible to calculate more than one aa-tAI score, and all these aa-tAI scores contributed to the final matrix. **Method 2)** The average tAI of all the codons coding for amino acid *x* in each window was calculated, similarly to Method 1, but a tAI score is not yet assigned. Instead, the average tAI is first determined for each amino acid present in both windows, and then average tAIs (each the average for a particular amino acid) are themselves averaged to come up with a single aa-tAI for each window. Then, a single tAI score is assigned to that gene by comparing the average aa-tAIs in each window similarly to above. Bold = method 1, italic = method 2. Original Δr quantiles means the same quantile boundaries used in the main analysis were used, whereas recalculated Δr quantiles are drawn from only those genes considered in this amino-acid adjusted analysis. The P value for χ^2 tests with fewer than 5 observations in any square was calculated by resampling the observations without replacement and noting how many times (r) the χ^2 value of the resampled set was greater than or equal to the observed. P was then calculated as $(r+1)/(n+1)$, where n is the number of iterations performed (1000). **A.** Upon controlling for differential amino acid content in the two windows as detailed above, the result that tAI cannot explain patterns of slowing is still robust. Additionally we no longer detect a decrease in the ability of tAI to explain pausing in the upper quantiles as observed in Table 1A. **B** and **C** show the effect of tAI (adjusted for amino acid use) in only those pairs of intra-transcript windows which have the same number of positive charges between them.
