# Supplementary material for: Positively Charged Residues Are the Major Determinants of Ribosomal Velocity
Source: PLoS Biol. 2013 Mar 12;11(3):e1001508. doi: 10.1371/journal.pbio.1001508 (PMC3595205; doi:10.1371/journal.pbio.1001508)
Supplement: Table S5 — Table 1 done again on the amino-acid-starved footprint set [29]. Only positive charge is systematically capable of explaining ribosomal slowing, including the severest slowing. Quantiles of the difference in average ribosomal density between the two windows identified within a transcript are shown, with q1 representing the smallest differences and q4 the largest. A score of 1 indicates the putative retarding feature is more present within the more occluded intra-transcript window; −1, less present; 0, present in both windows in equal amounts. A low codon optimality, if anything, tends to pair more with the less dense (faster translated) window. Similarly, not only do rare pairs and rare 6-mers tend to be found more often in the faster-translated window, but their presence decreases as the difference in degree of ribosomal slowing grows. Additionally, a greater likelihood of transcript secondary structure at or just before the identified window is associated not with the more occluded windows, but with the less dense (faster translated) ones, and the presence of secondary structure in fact decreases as the difference in ribosomal slowing between the windows increases. Positive charge, however, is consistently associated with the higher density (more slowly translated) window. (PDF) [file pbio.1001508.s025.pdf]

Table S5.

		$q1_{\Delta r}$ (count)	$q2_{\Delta r}$	$q3_{\Delta r}$	$q4_{\Delta r}$	χ^2 test P value (Bonferroni correction)
**A.**<br>**charge score**	**1**	**552**	**569**	**587**	**610**	**0.36**
	**0**	**249**	**241**	**266**	**250**	**0.73**
	**-1**	**471**	**462**	**419**	**412**	**0.11**
	**Binomial test on +1 and -1 charge score counts, P value (Bonferroni correction)**	**0.012 (0.048)**	**0.00095 (0.0038)**	**1.3e-07 (5.2e-07)**	**6.4e-10 (2.6e-09)**	**-**
**B.**<br>**tAI score**	**1**	**632**	**615**	**607**	**577**	**0.46**
	**0**	**0**	**0**	**0**	**0**	-
	**-1**	**640**	**657**	**665**	**695**	**0.50**
	**Binomial test on +1 and -1 tAI score counts, P value (Bonferroni correction)**	**0.84**	**0.25**	**0.11**	**0.0010 (0.0040)**	**-**
**C.**<br>**rare pair score**<br>*rare 6-mer score*	**1**	**176**<br>*267*	**175**<br>*256*	**179**<br>*234*	**116**<br>*156*	**0.00066 (0.0020)**<br>*3.3e-07 (9.9e-07)*
	**0**	**909**<br>*712*	**875**<br>*705*	**910**<br>*730*	**1022**<br>*773*	**0.0041 (0.012)**<br>*0.28*
	**-1**	**187**<br>*293*	**222**<br>*311*	**183**<br>*308*	**134**<br>*343*	**7.7e-05 (0.00023)**<br>*0.24*
	**Binomial test on +1 and -1 rare pair score counts, P value (Bonferroni correction)**	**0.60**<br>*0.29*	**0.021 (0.082)**<br>*0.023 (0.092)*	**0.87**<br>*0.0017 (0.0068)*	**0.28**<br>*< 2.2e-16 (8.8e-16)*	**-**
**C.**<br>**PARS score**<br>*conservative PARS score*	**1**	**103**<br>*335*	**84**<br>*297*	**79**<br>*297*	**58**<br>*300*	**0.0054 (0.022)**<br>*0.34*
	**0**	**499**<br>*0*	**492**<br>*0*	**519**<br>*0*	**543**<br>*0*	**0.38**<br>*-*
	**-1**	**107**<br>*374*	**133**<br>*412*	**111**<br>*412*	**108**<br>*409*	**0.27**<br>*0.46*
	**Binomial test on +1 and -1 rare pair score counts, P value (Bonferroni correction)**	**0.84**<br>*0.15*	**0.0011 (0.0044)**<br>*1.8e-05 (7.1e-05)*	**0.024 (0.096)**<br>*1.8e-05 (7.1e-05)*	**0.00013 (0.00051)**<br>*4.8e-05 (1.6e-05)*	**-**

Table S5. Table 1 done again on the amino acid-starved footprint set [29]**.** Only positive charge is systematically capable of explaining ribosomal slowing, including the severest slowing**.** Quantiles of the difference in average ribosomal density between the two windows identified within a transcript are shown, with q1 representing the smallest differences and q4 the largest. A score of 1 indicates the putative retarding feature is more present within the more occluded intra-transcript

window; -1, less present; 0, present in both windows in equal amounts. A low codon optimality, if anything, tends to pair more with the less dense (faster translated) window. Similarly, not only do rare pairs and rare 6-mers tend to be found more often in the faster-translated window, but their presence decreases as the difference in degree of ribosomal slowing grows. Additionally, a greater likelihood of transcript secondary structure at or just before the identified window is associated not with the more occluded windows, but with the less dense (faster translated) ones, and the presence of secondary structure in fact decreases as the difference in ribosomal slowing between the windows increases. Positive charge however is consistently associated with the higher density (more slowly-translated) window.
