# Supplementary material for: Positively Charged Residues Are the Major Determinants of Ribosomal Velocity
Source: PLoS Biol. 2013 Mar 12;11(3):e1001508. doi: 10.1371/journal.pbio.1001508 (PMC3595205; doi:10.1371/journal.pbio.1001508)
Supplement: Table S6 — Positive charge best explains the slowest translated regions within transcripts compared to other physiochemical properties of amino acids. While we find that positive charges slow ribosomes, we wanted to control for the effects of other physiochemical properties of amino acids, specifically hydropathy (Phe, Val, Leu, Ile, Met), polarity (Asn, Gln, Ser, Thr, Cys, Tyr), and negative charge (Asp, Glu). These groups of amino acids, however, do not lend themselves to the rpos/rprec30 analysis we carry out in the main text (see Figures 1–5) in the same way that positive charge does. The rpos/rprec30 plotting analysis is suited to positive charges because they cluster in a way that gives us reasonable sample sizes given our constraints—that is, the number of positive charges we require in the cluster and the additional requirement that there be no surrounding positive charges outside of the cluster. In the case of the other amino acid groups, there are either too many constituent members of the group and which are used too frequently (e.g., hydropathy) to define isolated “clusters” for investigation, or the amino acids are used too rarely as clusters away from positive charges, and are of insufficient cluster sizes to establish any slowing trends (e.g., negative charges). We therefore compared the effects of these other physiochemical properties of amino acids by comparing the amino acids encoded by the highest ribosomally occupied versus lowest occupied windows within genes. The analysis was carried out similarly to the way Table 1 was created in the main text, only this time counting different amino acids depending on the physiochemical property being investigated. We find that, on the whole, only positive charge can robustly explain the slowing patterns we observe. Quantiles of the difference in average ribosomal density between the two windows identified within a transcript are shown, with q1 representing the smallest differences and q4 the largest. A score of 1 indic [file pbio.1001508.s026.pdf]

Table S6.

		$q1_{\Delta r}$ (count)	$q2_{\Delta r}$	$q3_{\Delta r}$	$q4_{\Delta r}$	χ^2 test P value (Bonferroni correction)
A. hydropathy score	1	490	517	530	535	0.50
	0	215	191	216	214	0.56
	-1	541	537	499	496	0.34
	Binomial test on +1 and -1 charge score counts, P value (Bonferroni correction)	0.12	0.56	0.35	0.24	-
B. polarity score	1	557	576	580	627	0.21
	0	206	168	158	179	0.065
	-1	483	501	507	439	0.12
	Binomial test on +1 and -1 tAI score counts, P value (Bonferroni correction)	0.024 (0.096)	0.024 (0.096)	0.029 (0.12)	9.4e-09 (3.7e-08)	-
C. negative charge score	1	547	539	507	584	0.14
	0	270	271	239	273	0.39
	-1	429	435	499	388	0.0024 (0.0072)
	Binomial test on +1 and -1 rare pair score counts, P value (Bonferroni correction)	0.00018 (0.00072)	0.0010 (0.0040)	0.83	3.5e-10 (1.4e-9)	-
D. positive charge score	1	573	586	637	717	0.00014 (0.00043)
	0	258	259	236	207	0.0589 (0.18)
	-1	415	401	372	322	0.0038 (0.011)
	Binomial test on +1 and -1 rare pair score counts, P value (Bonferroni correction)	5.6e-07 (2.2e-06)	4.3e-09 (1.7e-08)	<2.2e-16 (8.8e-16)	<2.2e-16 (8.8e-16)	-

Table S6. Positive charge best explains the slowest translated regions within transcripts compared to other physiochemical properties of amino acids. While we find that positive charges slow ribosomes, we wanted to control for the effects of other physiochemical properties of amino acids, specifically hydropathy (Phe, Val, Leu, Ile, Met), polarity (Asn, Gln, Ser, Thr, Cys, Tyr) and negative charge (Asp, Glu). These groups of amino acids, however, do not lend themselves to the r_{pos}/r_{prec30} analysis we carry out in the main text (See Figures 1-5) in the same way that positive charge does. The r_{pos}/r_{prec30} analysis is suited to positive charges because they cluster

in a way that gives us reasonable sample sizes given our constraints, i.e. the number of positive charges we require in the cluster and the additional requirement that there be no surrounding positive charges outside of the cluster. In the case of the other amino acid groups, there are either too many constituent members of the group and which are used too frequently (e.g. hydropathy) to define isolated 'clusters' for investigation, or the amino acids are used too rarely as clusters away from positive charges, and are of insufficient cluster sizes to establish any slowing trends (e.g. negative charges). We therefore compared the effects of these other physiochemical properties of amino acids by comparing the amino acids encoded by the highest-ribosomally occupied vs. lowest-occupied windows within genes. The analysis was carried out similarly to the way Table 1 was created in the main text, only this time counting different amino acids depending on the physiochemical property being investigated. We find that, on the whole, only positive charge can robustly explain the slowing patterns we observe. Quantiles of the difference in average ribosomal density between the two windows identified within a transcript are shown, with q1 representing the smallest differences and q4 the largest. A score of 1 indicates the putative retarding feature is more present within the more occluded intra-transcript window; -1, less present; 0, present in both windows in equal amounts. **A.** Hydrophobic residues (Phe, Val, Leu, Ile, Met) cannot explain increased slowing as the difference in translation speed between the two windows increases (χ^2 P = 0.98). Additionally the proportion of genes which pass the hydrophobicity test compared to failing it is only significant in the fourth quantile (q4) (binomial P = 0.023). **B.** Polar residues (Asn, Gln, Ser, Thr, Cys, Tyr) cannot explain increased slowing as the difference in translation speed between the two windows increases (χ^2 P = 0.21). Additionally the proportion of genes which pass the polarity test compared to failing it is only significant in the fourth quantile (q4) (binomial P = 3.7e-08). **C.** Negative charges (Asp, Glu) cannot explain increased slowing as the difference in translation speed between the two windows increases (χ^2 P = 0.14). Additionally the number of genes which pass or fail the negative charge score test in the third quantile (q3) is not significantly different (binomial P = 0.83). **D.** Positive charge score, from Table 1, is shown for purposes of comparison.
