# Supplementary material for: Positively Charged Residues Are the Major Determinants of Ribosomal Velocity
Source: PLoS Biol. 2013 Mar 12;11(3):e1001508. doi: 10.1371/journal.pbio.1001508 (PMC3595205; doi:10.1371/journal.pbio.1001508)
Supplement: Table S7 — Positive charge explains slowing better than amino acid hydrophobicity. Quantiles of the difference in average ribosomal density between the two windows identified within a transcript are shown, with q1 representing the smallest differences and q4 the largest. A score of 1 indicates the putative retarding feature is more present within the more occluded intra-transcript window; −1, less present; 0, present in both windows in equal amounts. (A–C) In those genes that fail the positive charge test (charge score = 0 or −1), we find that hydrophobicity cannot explain the increased slowing in these windows either (this table, χ2 tests). For this reason we consider that while amino acids with hydrophobic side chains may be used more often in the vicinity of positive charge (this table, binomial tests), perhaps for certain structural motifs or because of the types of genes under consideration, they cannot be responsible for the major slowing effect. (D–F) Positive charge can explain the slowing in genes where hydrophobicity cannot. (PDF) [file pbio.1001508.s027.pdf]

Table S7.

		$q1_{\Delta r}$ (count)	$q2_{\Delta r}$	$q3_{\Delta r}$	$q4_{\Delta r}$	χ^2 test P value (Bonferroni correction)
A. hydropathy score when charge score = 0	1	101	107	103	104	0.98
	0	54	36	50	36	0.11
	-1	101	106	89	67	0.019 (0.056)
	Binomial test on +1 and -1 charge score counts, P value (Bonferroni correction)	1	1	0.35	0.0057 (0.023)	-
B. hydropathy score when charge score = -1	1	199	207	191	186	0.73
	0	70	52	60	53	0.32
	-1	149	149	113	84	2.5e-05 (7.5e-05)
	Binomial test on +1 and -1 tAI score counts, P value (Bonferroni correction)	0.0085 (0.034)	0.0025 (0.010)	9.0e-06 (3.6e-05)	5.0e-10 (2.0e-09)	-
C. hydropathy score when charge score = 0 or -1	1	300	314	294	290	0.78
	0	124	88	110	89	0.031 (0.093)
	-1	250	255	202	151	3.1e-07 (9.3e-07)
	Binomial test on +1 and -1 rare pair score counts, P value (Bonferroni correction)	0.037 (0.15)	0.015 (0.060)	4.2e-05 (0.00017)	3.5e-11 (1.4e-10)	-
D. charge score when hydropathy score = 0	1	91	103	106	125	0.13
	0	54	36	50	36	0.11
	-1	70	52	60	53	0.32
	Binomial test on +1 and -1 charge score counts, P value (Bonferroni correction)	0.11	5.1e-05 (0.00020)	0.00044 (0.0018)	6.9e-08 (2.8e-07)	-

**E. charge score when hydropathy score = -1**	**1**	**291**	**282**	**297**	**345**	**0.049 (0.15)**
	**0**	**101**	**106**	**89**	**67**	**0.019 (0.057)**
	**-1**	**149**	**149**	**113**	**84**	**2.5e-05 (7.5e-05)**
	**Binomial test on +1 and -1 tAI score counts, P value (Bonferroni correction)**	**1.2e-11 (4.8e-11)**	**1.5e-10 (6.0e-10)**	**2.2e-16 (8.8e-16)**	**2.2e-16 (8.8e-16)**	**-**
**F. charge score when hydropathy score = 0 or -1**	**1**	**382**	**385**	**403**	**470**	**0.0063 (0.019)**
	**0**	**155**	**142**	**139**	**103**	**0.011 (0.033)**
	**-1**	**219**	**201**	**173**	**137**	**0.00010 (0.00030)**
	**Binomial test on +1 and -1 rare pair score counts, P value (Bonferroni correction)**	**3.0e-11 (1.2e-10)**	**2.5e-14 (1.0e-13)**	**2.2e-16 (8.8e-16)**	**2.2e-16 (8.8e-16)**	**-**

Table S7. Positive charge explains slowing better than amino acid hydrophobicity. Quantiles of the difference in average ribosomal density between the two windows identified within a transcript are shown, with q1 representing the smallest differences and q4 the largest. A score of 1 indicates the putative retarding feature is more present within the more occluded intra-transcript window; -1, less present; 0, present in both windows in equal amounts. **A – C.** In those genes which fail the positive charge test (charge score = 0 or -1), we find that hydrophobicity cannot explain the increased slowing in these windows either (this table, χ^2 tests). For this reason we consider that while amino acids with hydrophobic side chains may be used more often in the vicinity of positive charge (this table, binomial tests), perhaps for certain structural motifs or because of the types of genes under consideration, they cannot responsible for the major slowing effect. **D – F.** Positive charge can explain the slowing in genes where hydrophobicity cannot.
