# Supplementary material for: Positively Charged Residues Are the Major Determinants of Ribosomal Velocity
Source: PLoS Biol. 2013 Mar 12;11(3):e1001508. doi: 10.1371/journal.pbio.1001508 (PMC3595205; doi:10.1371/journal.pbio.1001508)
Supplement: Table S8 — Positive charge explains slowing better than amino acid polarity. Quantiles of the difference in average ribosomal density between the two windows identified within a transcript are shown, with q1 representing the smallest differences and q4 the largest. A score of 1 indicates the putative retarding feature is more present within the more occluded intra-transcript window; −1, less present; 0, present in both windows in equal amounts. (A–C) In those genes that fail the positive charge test (charge score = 0 or −1), we find that polarity cannot explain the increased slowing in these windows either (this table, χ2 tests). For this reason we consider that while amino acids with polar side chains may be used more often in the vicinity of positive charge (this table, binomial tests), perhaps for certain structural motifs or because of the types of genes under consideration, they cannot be responsible for the major slowing effect. (D–F) Positive charge can explain the slowing in some genes where polarity cannot. (PDF) [file pbio.1001508.s028.pdf]

**Table S8.**

| | | $q1_{\Delta r}$ (count) | $q2_{\Delta r}$ | $q3_{\Delta r}$ | $q4_{\Delta r}$ | $\chi^2$ test P value (Bonferroni correction) |
|---|---|---|---|---|---|---|
| A. polarity score when positive charge score = 0 | 1 | 112 | 114 | 115 | 107 | 0.95 |
| | 0 | 51 | 39 | 43 | 35 | 0.34 |
| | -1 | 93 | 96 | 84 | 65 | 0.074 |
| | Binomial test on +1 and -1 charge score counts, P value (Bonferroni correction) | 0.21 | 0.24 | 0.033 (0.132) | 0.0017 (0.0068) | - |
| B. polarity score when positive charge score = -1 | 1 | 253 | 246 | 216 | 211 | 0.12 |
| | 0 | 71 | 58 | 45 | 33 | 0.0014 (0.0042) |
| | -1 | 94 | 104 | 103 | 79 | 0.24 |
| | Binomial test on +1 and -1 tAI score counts, P value (Bonferroni correction) | 2.2e-16 (8.8e-16) | 2.1e-14 (8.5e-14) | 2.4e-10 (9.4e-10) | 4.8e-15 (1.9e-14) | - |
| C. polarity score when positive charge score = 0 or -1 | 1 | 365 | 360 | 331 | 318 | 0.21 |
| | 0 | 122 | 97 | 88 | 68 | 0.0011 (0.0033) |
| | -1 | 187 | 200 | 187 | 144 | 0.019 (0.057) |
| | Binomial test on +1 and -1 rare pair score counts, P value (Bonferroni correction) | 3.0e-14 (1.2e-13) | 1.3e-11 (5.4e-11) | 2.6e-10 (1.0e-09) | 3.8e-16 (1.5e-15) | - |
| D. positive charge score when polarity score = 0 | 1 | 84 | 71 | 70 | 111 | 0.0046 (0.014) |
| | 0 | 51 | 39 | 43 | 35 | 0.34 |
| | -1 | 71 | 58 | 45 | 33 | 0.0014 (0.0041) |
| | Binomial test on +1 and -1 charge score counts, P value | 0.34 | 0.29 | 0.025 (0.1) | 4.6e-11 (1.8e-10) | - |

| | | | | | | |
|---|---|---|---|---|---|---|
| | (Bonferroni correction) | | | | | |
| E. positive charge score when polarity score = -1 | 1 | 296 | 301 | 320 | 295 | 0.79 |
| | 0 | 93 | 96 | 84 | 65 | 0.074 |
| | -1 | 94 | 104 | 103 | 79 | 0.24 |
| | Binomial test on +1 and -1 tAI score counts, P value (Bonferroni correction) | 2.2e-16 (8.8e-16) | 2.2e-16 (8.8e-16) | 2.2e-16 (8.8e-16) | 2.2e-16 (8.8e-16) | - |
| F. positive charge score when polarity score = 0 or -1 | 1 | 380 | 372 | 390 | 406 | 0.65 |
| | 0 | 144 | 135 | 127 | 100 | 0.036 (0.11) |
| | -1 | 165 | 162 | 148 | 112 | 0.0071 (0.021) |
| | Binomial test on +1 and -1 rare pair score counts, P value (Bonferroni correction) | 2.2e-16 (8.8e-16) | 2.2e-16 (8.8e-16) | 2.2e-16 (8.8e-16) | 2.2e-16 (8.8e-16) | - |

**Table S8. Positive charge explains slowing better than amino acid polarity.** Quantiles of the difference in average ribosomal density between the two windows identified within a transcript are shown, with q1 representing the smallest differences and q4 the largest. A score of 1 indicates the putative retarding feature is more present within the more occluded intra-transcript window; -1, less present; 0, present in both windows in equal amounts. **A – C.** In those genes which fail the positive charge test (charge score = 0 or -1), we find that polarity cannot explain the increased slowing in these windows either (this table, $\chi^2$ tests). For this reason we consider that while amino acids with polar side chains may be used more often in the vicinity of positive charge (this table, binomial tests), perhaps for certain structural motifs or because of the types of genes under consideration, they cannot responsible for the major slowing effect. **D – F.** Positive charge can explain the slowing in some genes where polarity cannot.
