# Supplementary material for: Positively Charged Residues Are the Major Determinants of Ribosomal Velocity
Source: PLoS Biol. 2013 Mar 12;11(3):e1001508. doi: 10.1371/journal.pbio.1001508 (PMC3595205; doi:10.1371/journal.pbio.1001508)
Supplement: Table S9 — Positive charge explains slowing better than negative charge. Quantiles of the difference in average ribosomal density between the two windows identified within a transcript are shown, with q1 representing the smallest differences and q4 the largest. A score of 1 indicates the putative retarding feature is more present within the more occluded intra-transcript window; −1, less present; 0, present in both windows in equal amounts. (A–C) In those genes that fail the positive charge test (charge score = 0 or −1), we find that negatively charged amino acids cannot explain the increased slowing in these windows either (this table, χ2 tests). For this reason we consider that while amino acids with negatively charged side chains may be used more often in the vicinity of positive charge (this table, binomial tests), perhaps for certain structural motifs or because of the types of genes under consideration, they cannot be responsible for the major slowing effect. (D–F) Positive charge can explain the slowing in genes where negative charge cannot. (PDF) [file pbio.1001508.s029.pdf]

Table S9.

		$q1_{\Delta r}$ (count)	$q2_{\Delta r}$	$q3_{\Delta r}$	$q4_{\Delta r}$	χ^2 test P value (Bonferroni correction)
A. negative charge score when positive charge score = 0	1	106	114	105	100	0.81
	0	52	52	49	42	0.71
	-1	98	83	88	65	0.076
	Binomial test on +1 and -1 charge score counts, P value (Bonferroni correction)	0.62	0.032 (0.13)	0.25	0.0079 (0.032)	-
B. negative charge score when positive charge score = -1	1	213	208	176	176	0.10
	0	89	76	64	66	0.15
	-1	116	124	124	81	0.010 (0.030)
	Binomial test on +1 and -1 tAI score counts, P value (Bonferroni correction)	9.8e-08 (3.9e-07)	4.7e-06 (1.9e-05)	0.0032 (0.013)	3.0e-09 (1.2e-08)	-
C. negative charge score when positive charge score = 0 or -1	1	319	322	281	276	0.11
	0	141	128	113	108	0.14
	-1	214	207	212	146	0.0010 (0.0030)
	Binomial test on +1 and -1 rare pair score counts, P value (Bonferroni correction)	6.2e-06 (2.5e-05)	6.5e-07 (2.6e-06)	0.0022 (0.0088)	2.5e-10 (9.9e-10)	-
D. positive charge score when negative charge score = 0	1	129	143	126	165	0.081
	0	52	52	49	42	0.71
	-1	89	76	64	66	0.15

	**Binomial test on +1 and -1 charge score counts, P value (Bonferroni correction)**	**0.0081 (0.032)**	**7.0e-06 (2.8e-05)**	**8.1e-06 (3.2e-05)**	**5.8e-11 (2.3e-10)**	**-**
**E. positive charge score when negative charge score = -1**	**1**	**215**	**228**	**287**	**242**	**0.0070 (0.021)**
	**0**	**98**	**83**	**88**	**65**	**0.076**
	**-1**	**116**	**124**	**124**	**81**	**0.010 (0.030)**
	**Binomial test on +1 and -1 tAI score counts, P value (Bonferroni correction)**	**5.8e-08 (2.3e-07)**	**3.2e-08 (1.3e-07)**	**5.5e-16 (2.2e-15)**	**2.2e-16 (8.8e-16)**	**-**
**F. positive charge score when negative charge score = 0 or -1**	**1**	**344**	**371**	**413**	**407**	**0.042 (0.13)**
	**0**	**150**	**135**	**137**	**107**	**0.059**
	**-1**	**205**	**200**	**188**	**147**	**0.011 (0.033)**
	**Binomial test on +1 and -1 rare pair score counts, P value (Bonferroni correction)**	**3.2e-09 (1.3e-08)**	**7.6e-13 (3.1e-12)**	**2.2e-16 (8.8e-16)**	**2.2e-16 (8.8e-16)**	**-**

Table S9. Positive charge explains slowing better than negative charge. Quantiles of the difference in average ribosomal density between the two windows identified within a transcript are shown, with q1 representing the smallest differences and q4 the largest. A score of 1 indicates the putative retarding feature is more present within the more occluded intra-transcript window; -1, less present; 0, present in both windows in equal amounts. **A – C.** In those genes which fail the positive charge test (charge score = 0 or -1), we find that negatively charged amino acids cannot explain the increased slowing in these windows either (this table, χ^2 tests). For this reason we consider that while amino acids with negatively charged side chains may be used more often in the vicinity of positive charge (this table, binomial tests), perhaps for certain structural motifs or because of the types of genes under consideration, they cannot responsible for the major slowing effect. **D – F.** Positive charge can explain the slowing in genes where negative charge cannot.
