# Supplementary material for: Positively Charged Residues Are the Major Determinants of Ribosomal Velocity
Source: PLoS Biol. 2013 Mar 12;11(3):e1001508. doi: 10.1371/journal.pbio.1001508 (PMC3595205; doi:10.1371/journal.pbio.1001508)
Supplement: Table S10 — The relationship of charge score to tAI score. Quantiles of the difference in average ribosomal occlusion between the two windows identified within a transcript are shown, with q1 representing the smallest differences and q4 the largest. A score of 1 indicates the putative retarding feature is more present within the more occluded intra-transcript window; −1, less present; 0, present in both windows in equal amounts. (A) The ability of charge to explain slowing (charge score of 1) cannot be explained by concomitant use of suboptimal codons. A charge score of 1 more commonly pairs with a tAI score, which cannot explain slowing (tAI score of −1), and increasingly so as the difference in ribosomal speeds between the two windows grows. (B) These tAI scores are drawn from transcripts for which both intra-transcript windows have the same number of charges (charge score = 0) and hence such comparisons should be controlled for the effect of positive charge on ribosomal speed. Different tAI scores are equally distributed among quantiles, indicating the inability of tAI to predict either ribosomal slowing or the degree of ribosomal slowing even in the absence of an effect of charge on ribosomal speed. (C) tAI does not systematically account for slowing in windows for which increased charge pairs with the faster window. (PDF) [file pbio.1001508.s030.pdf]

Table S10.

		$q1_{\Delta r}$ (count)	$q2_{\Delta r}$	$q3_{\Delta r}$	$q4_{\Delta r}$	χ^2 test P value (Bonferroni correction)
**A.**	**charge score 1**<br>**tAI score 1**	271	272	281	284	0.93
	**charge score 1**<br>**tAI score -1**	302	314	356	433	1.4e-06<br>(2.8e-06)
	**Binomial test P value (Bonferroni correction)**	0.21	0.090	0.0033<br>(0.013)	2.9e-08<br>(1.2e-07)	-
**B.**	**charge score 0**<br>**tAI score 1**	116	130	111	101	0.28<br>(0.56)
	**charge score 0**<br>**tAI score -1**	142	129	125	106	0.15<br>(0.30)
	**Binomial test P value (Bonferroni correction)**	0.12	1.0	0.40	0.78	-
**C.**	**charge score -1**<br>**tAI score 1**	203	195	171	140	0.0036<br>(0.0072)
	**charge score -1**<br>**tAI score -1**	212	206	201	182	0.47
	**Binomial test P value (Bonferroni correction)**	0.69	0.62	0.13	0.022<br>(0.09)	-

Table S10. The relationship of charge score to tAI score. Quantiles of the difference in average ribosomal occlusion between the two windows identified within a transcript are shown, with q1 representing the smallest differences and q4 the largest. A score of 1 indicates the putative retarding feature is more present within the more occluded intra-transcript window; -1, less present; 0, present in both windows in equal amounts. **A.** The ability of charge to explain slowing (charge score of 1) cannot be explained by concomitant use of suboptimal codons. A charge score of 1 more commonly pairs with a tAI score which cannot explain slowing (tAI score of -1), and increasingly so as the difference in ribosomal speeds between the two windows grows. **B**. These tAI scores are drawn from transcripts for which both intra-transcript windows have the same number of charges (charge score = 0) and hence such comparisons should be controlled for the effect of positive charge on ribosomal speed. Different tAI scores are equally distributed among quantiles, indicating the inability of tAI to predict either ribosomal slowing or the degree of ribosomal slowing even in the absence of an effect of charge on ribosomal speed. **C**. tAI does not systematically account for slowing in windows for which increased charge pairs with the faster window.
