# Supplementary material for: Positively Charged Residues Are the Major Determinants of Ribosomal Velocity
Source: PLoS Biol. 2013 Mar 12;11(3):e1001508. doi: 10.1371/journal.pbio.1001508 (PMC3595205; doi:10.1371/journal.pbio.1001508)
Supplement: Table S11 — The relationship of rare pair score to charge score. Quantiles of the difference in average ribosomal occlusion between the two windows identified within a transcript are shown, with q1 representing the smallest differences and q4 the largest. A score of 1 indicates the putative retarding feature is more present within the more occluded intra-transcript window; −1, less present; 0, present in both windows in equal amounts. (A) The ability of charge to explain slowing (charge score of 1) cannot be explained by concomitant use of rare pairs. A charge score of 1, if anything, tends to pair with a rare pair score that cannot explain slowing (rare pair score of −1). (B) These rare pair scores are drawn from transcripts for which both intra-transcript windows have the same number of charges (charge score = 0) and hence such comparisons should be controlled for the effect of positive charge on ribosomal speed. Different rare pair scores are equally distributed among quantiles, indicating the inability of rare pairs to predict ribosomal slowing. Additionally, as the difference in the degree of ribosomal slowing increases (i.e., moving from q1 to q4), the number of rare pairs found in the higher occupancy window decreases (χ2 test), demonstrating rare pairs cannot predict the magnitude of slowing even in the absence of an effect of charge on ribosomal speed. (C) Rare pairs do not systematically account for slowing in windows for which increased charge pairs with the faster window. (PDF) [file pbio.1001508.s031.pdf]

Table S11.

		$q1_{\Delta r}$ (count)	$q2_{\Delta r}$	$q3_{\Delta r}$	$q4_{\Delta r}$	χ^2 test P value (Bonferroni correction)
**A.**	**charge score 1 rare pair score 1**	70	81	64	48	0.034 (0.068)
	**charge score 1 rare pair score -1**	92	89	98	79	0.55
	**Binomial test P value (Bonferroni correction)**	0.10	0.59	0.0093 (0.03)	0.0075 (0.03)	
**B.**	**charge score 0 rare pair score 1**	34	36	31	14	0.015 (0.030)
	**charge score 0 rare pair score -1**	46	41	42	20	0.012 (0.024)
	**Binomial test P value (Bonferroni correction)**	0.22	0.65	0.24	0.39	
**C.**	**charge score -1 rare pair score 1**	71	62	49	24	2.1e-05 (4.2e-05)
	**charge score -1 rare pair score -1**	75	52	56	24	1.2e-05 (2.4e-05)
	**Binomial test P value (Bonferroni correction)**	0.80	0.40	0.56	1.0	

Table S11. The relationship of rare pair score to charge score. Quantiles of the difference in average ribosomal occlusion between the two windows identified within a transcript are shown, with q1 representing the smallest differences and q4 the largest. A score of 1 indicates the putative retarding feature is more present within the more occluded intra-transcript window; -1, less present; 0, present in both windows in equal amounts. **A.** The ability of charge to explain slowing (charge score of 1) cannot be explained by concomitant use of rare pairs. A charge score of 1, if anything, tends to pair with a rare pair score which cannot explain slowing (rare pair score of -1). **B**. These rare pair scores are drawn from transcripts for which both intra-transcript windows have the same number of charges (charge score = 0) and hence such comparisons should be controlled for the effect of positive charge on ribosomal speed. Different rare pair scores are equally distributed among quantiles, indicating the inability of rare pairs to predict ribosomal slowing. Additionally, as the difference in the degree of ribosomal slowing increases (i.e. moving from q1 to q4), the number of rare pairs found in the higher occupancy window decreases (χ^2 test), demonstrating rare pairs cannot predict the magnitude of slowing even in the absence of an effect of charge on ribosomal speed. **C**. Rare pairs do not systematically account for slowing in windows for which increased charge pairs with the faster window.
