# Supplementary material for: Positively Charged Residues Are the Major Determinants of Ribosomal Velocity
Source: PLoS Biol. 2013 Mar 12;11(3):e1001508. doi: 10.1371/journal.pbio.1001508 (PMC3595205; doi:10.1371/journal.pbio.1001508)
Supplement: Table S12 — The relationship of PARS score (double strandedness) to charge score. Quantiles of the difference in average ribosomal occlusion between the two windows identified within a transcript are shown, with q1 representing the smallest differences and q4 the largest. A score of 1 indicates the putative retarding feature is more present within the more occluded intra-transcript window; −1, less present; 0, present in both windows in equal amounts. (PDF) [file pbio.1001508.s032.pdf]

Table S12.

		$q1_{\Delta r}$ (count)	$q2_{\Delta r}$	$q3_{\Delta r}$	$q4_{\Delta r}$	χ^2 test P value (Bonferroni correction)
**A.**	**charge score 1 PARS score 1**	40	32	48	29	0.12
	**charge score 1 PARS score -1**	78	67	82	73	0.64
	**Binomial test P value (Bonferroni correction)**	0.00060 (0.0024)	0.00056 (0.0022)	0.0036 (0.014)	1.6e-05 (6.4e-05)	-
**B.**	**charge score 0 PARS score 1**	24	13	12	11	0.062
	**charge score 0 PARS score -1**	86	77	84	61	0.17
	**Binomial test P value (Bonferroni correction)**	2.2e-09 (8.9e-09)	3.2e-12 (1.3e-11)	1.8e-14 (7.3e-14)	1.5e-09 (6.2e-09)	-
**C.**	**charge score -1 PARS score 1**	22	27	21	15	0.33
	**charge score -1 PARS score -1**	44	37	21	23	0.008 (0.016)
	**Binomial test P value (Bonferroni correction)**	0.0092 (0.037)	0.26	1	0.26	-
**D.**	**charge score 1 conservative PARS score 1**	140	126	163	155	0.14
	**charge score 1 conservative PARS score -1**	200	248	233	246	0.095
	**Binomial test P value (Bonferroni correction)**	0.0013 (0.0052)	2.8e-10 (1.1e-09)	0.00051 (0.0020)	6.4e-06 (2.6e-05)	-
**E.**	**charge score 0 conservative PARS score 1**	72	52	52	54	0.18
	**charge score 0 conservative PARS score -1**	86	77	84	61	0.17
	**Binomial test P value (Bonferroni correction)**	0.30	0.034 (0.14)	0.0076 (0.030)	0.58	-
**F.**	**charge score -1 conservative PARS score 1**	90	94	75	85	0.50
	**charge score -1 conservative PARS score -1**	121	111	101	108	0.60
	**Binomial test P value (Bonferroni correction)**	0.039 (0.16)	0.26	0.060	0.11	-

Table S12. The relationship of PARS score (double strandedness) to charge score. Quantiles of the difference in average ribosomal occlusion between the two windows identified within a transcript are shown, with q1 representing the smallest differences and q4 the largest. A score of 1 indicates the putative retarding feature is more present within the more occluded intra-transcript window; -1, less present; 0, present in both windows in equal amounts.
