# Supplementary material for: Positively Charged Residues Are the Major Determinants of Ribosomal Velocity
Source: PLoS Biol. 2013 Mar 12;11(3):e1001508. doi: 10.1371/journal.pbio.1001508 (PMC3595205; doi:10.1371/journal.pbio.1001508)
Supplement: Table S13 — Table 1 done again, allowing the lower occupancy window to have a ribosomal occupancy of 0. (PDF) [file pbio.1001508.s033.pdf]

# Table S13.

| | | $q1_{\Delta r}$ (count) | $q2_{\Delta r}$ | $q3_{\Delta r}$ | $q4_{\Delta r}$ | $\chi^2$ test P value (Bonferroni correction) |
|---|---|---|---|---|---|---|
| **A.**<br>**charge score** | **1** | **578** | **630** | **700** | **766** | **1.3e-06 (3.8e-06)** |
| | **0** | **270** | **256** | **255** | **242** | **0.67** |
| | **-1** | **496** | **457** | **388** | **335** | **5.2e-08 (1.6e-07)** |
| | **Binomial test on +1 and -1 charge score counts, P value (Bonferroni correction)** | **0.013 (0.052)** | **1.7e-07 (6.8e-07)** | **< 2.2e-16 (8.8e-16)** | **< 2.2e-16 (8.8e-16)** | **-** |
| **B.**<br>**tAI score** | **1** | **576** | **600** | **601** | **551** | **0.41** |
| | **0** | **0** | **0** | **0** | **0** | **-** |
| | **-1** | **768** | **743** | **742** | **792** | **0.53** |
| | **Binomial test on +1 and -1 tAI score counts, P value (Bonferroni correction)** | **1.8e-07 (7.2e-07)** | **0.00011 (0.00042)** | **0.00013 (0.00053)** | **5.2e-11 (2.1e-10)** | **-** |
| **C.**<br>**rare pair score**<br>*rare 6-mer score* | **1** | **180**<br>*257* | **184**<br>*205* | **160**<br>*177* | **92**<br>*115* | **9.9e-08 (3.0e-07)**<br>*4.8e-12 (1.4e-11)* |
| | **0** | **904**<br>*712* | **907**<br>*711* | **958**<br>*730* | **1101**<br>*868* | **7.6e-06 (2.3e-05)**<br>*4.6e-05 (0.00014)* |
| | **-1** | **260**<br>*375* | **252**<br>*427* | **225**<br>*436* | **150**<br>*360* | **2.0e-07 (6.0e-07)**<br>*0.014 (0.042)* |
| | **Binomial test on +1 and -1 rare pair score counts, P value (Bonferroni correction)** | **0.00016 (0.00064)**<br>*3.1e-06 (1.2e-05)* | **0.0013 (0.0052)**<br>*<2.2e-16 (8.8e-16)* | **0.0011 (0.0044)**<br>*<2.2e-16 (8.8e-16)* | **0.00023 (0.00092)**<br>*<2.2e-16 (8.8e-16)* | **-** |
| **C.**<br>**PARS score**<br>*conservative PARS score* | **1** | **88**<br>*301* | **88**<br>*269* | **88**<br>*289* | **59**<br>*304* | **0.05**<br>*0.46* |
| | **0** | **491**<br>*0* | **510**<br>*0* | **516**<br>*0* | **554**<br>*0* | **0.26**<br>*-* |
| | **-1** | **155**<br>*433* | **136**<br>*465* | **130**<br>*445* | **121**<br>*430* | **0.21**<br>*0.64* |
| | **Binomial test on +1 and -1 rare pair score counts, P value (Bonferroni correction)** | **2.1e-05 (8.2e-05)**<br>*1.2e-06 (5.0e-05)* | **0.0016 (0.0065)**<br>*4.5e-13 (1.8e-12)* | **0.0054 (0.022)**<br>*9.4e-09 (4.4e-06)* | **4.4e-06 (1.8e-05)**<br>*3.8e-06 (1.5e-05)* | **-** |

**Table S13. Table 1 done again, allowing the lower-occupancy window to have a ribosomal occupancy of 0.**
