# Supplementary material for: Positively Charged Residues Are the Major Determinants of Ribosomal Velocity
Source: PLoS Biol. 2013 Mar 12;11(3):e1001508. doi: 10.1371/journal.pbio.1001508 (PMC3595205; doi:10.1371/journal.pbio.1001508)
Supplement: Table S14 — Table 1 done again on the non-redundant footprint location set. (PDF) [file pbio.1001508.s034.pdf]

Table S14.

		$q1_{\Delta r}$ (count)	$q2_{\Delta r}$	$q3_{\Delta r}$	$q4_{\Delta r}$	χ^2 test P value (Bonferroni correction)
**A.**<br>**charge score**	**1**	**571**	**591**	**637**	**708**	**0.00051 (0.0015)**
	**0**	**267**	**241**	**256**	**213**	**0.08**
	**-1**	**419**	**425**	**363**	**336**	**0.0022 (0.0065)**
	**Binomial test on +1 and -1 charge score counts, P value (Bonferroni correction)**	**1.5e-06 (6.0e-06)**	**2.1e-07 (8.4e-07)**	**< 2.2e-16 (8.8e-16)**	**< 2.2e-16 (8.8e-16)**	**-**
**B.**<br>**tAI score**	**1**	**595**	**589**	**573**	**539**	**0.35**
	**0**	**0**	**0**	**0**	**0**	**-**
	**-1**	**662**	**668**	**683**	**718**	**0.45**
	**Binomial test on +1 and -1 tAI score counts, P value (Bonferroni correction)**	**0.06 (0.24)**	**0.03 (0.12)**	**0.0021 (8.4e-03)**	**4.9e-07 (2.0e-06)**	**-**
**C.**<br>**rare pair score**<br>*rare 6-mer score*	**1**	**181**<br>*126*	**162**<br>*107*	**152**<br>*85*	**87**<br>*46*	**1.7e-07 (5.0e-07)**<br>*1.8e-08 (5.4e-08)*
	**0**	**858**<br>*382*	**912**<br>*401*	**913**<br>*425*	**1048**<br>*499*	**1.0e-06 (3.0e-04)**<br>*0.00035 (1.1e-03)*
	**-1**	**218**<br>*199*	**183**<br>*198*	**191**<br>*196*	**122**<br>*161*	**4.4e-06 (1.3e-05)**<br>*0.15*
	**Binomial test on +1 and -1 rare pair score counts, P value (Bonferroni correction)**	**0.07**<br>*6.1e-05 (2.4e-04)*	**0.28**<br>*2.1e-07 (8.4e-07)*	**0.040 (0.16)**<br>*3.0e-11 (1.2e-10)*	**0.018 (0.072)**<br>*3.8e-16 (1.5e-15)*	**-**
**C.**<br>**PARS score**<br>*conservative PARS score*	**1**	**86**<br>*301*	**72**<br>*271*	**80**<br>*290*	**57**<br>*292*	**0.093**<br>*0.065*
	**0**	**466**<br>*0*	**509**<br>*0*	**500**<br>*0*	**543**<br>*0*	**0.11**<br>*-*
	**-1**	**154**<br>*405*	**125**<br>*435*	**126**<br>*416*	**107**<br>*415*	**0.032 (0.096)**<br>*0.77*
	**Binomial test on +1 and -1 rare pair score counts, P value (Bonferroni correction)**	**1.3e-05 (5.2e-05)**<br>*0.00010 (0.00040)*	**0.00020 (0.00080)**<br>*7.2e-10 (2.9e-09)*	**0.0017 (0.0068)**<br>*2.4e-06 (9.6e-06)*	**0.00012 (0.00048)**<br>*4.2e-06 (1.7e-05)*	**-**

Table S14. Table 1 done again on the non-redundant footprint location set.
